# CHARMM at 45: Enhancements in Accessibility, Functionality, and Speed

**DOI:** 10.1021/acs.jpcb.4c04100

**Published:** 2024-09-20

**Authors:** Wonmuk Hwang, Steven L. Austin, Arnaud Blondel, Eric D. Boittier, Stefan Boresch, Matthias Buck, Joshua Buckner, Amedeo Caflisch, Hao-Ting Chang, Xi Cheng, Yeol Kyo Choi, Jhih-Wei Chu, Michael F. Crowley, Qiang Cui, Ana Damjanovic, Yuqing Deng, Mike Devereux, Xinqiang Ding, Michael F. Feig, Jiali Gao, David R. Glowacki, James E. Gonzales, Mehdi Bagerhi Hamaneh, Edward D. Harder, Ryan L. Hayes, Jing Huang, Yandong Huang, Phillip S. Hudson, Wonpil Im, Shahidul M. Islam, Wei Jiang, Michael R. Jones, Silvan Käser, Fiona L. Kearns, Nathan R. Kern, Jeffery B. Klauda, Themis Lazaridis, Jinhyuk Lee, Justin A. Lemkul, Xiaorong Liu, Yun Luo, Alexander D. MacKerell, Dan T. Major, Markus Meuwly, Kwangho Nam, Lennart Nilsson, Victor Ovchinnikov, Emanuele Paci, Soohyung Park, Richard W. Pastor, Amanda R. Pittman, Carol Beth Post, Samarjeet Prasad, Jingzhi Pu, Yifei Qi, Thenmalarchelvi Rathinavelan, Daniel R. Roe, Benoit Roux, Christopher N. Rowley, Jana Shen, Andrew C. Simmonett, Alexander J. Sodt, Kai Töpfer, Meenu Upadhyay, Arjan van der Vaart, Luis Itza Vazquez-Salazar, Richard M. Venable, Luke C. Warrensford, H. Lee Woodcock, Yujin Wu, Charles L. Brooks, Bernard R. Brooks, Martin Karplus

**Affiliations:** 1 Department of Biomedical Engineering, Texas A&M University, College Station, Texas 77843, United States; 2 Department of Materials Science and Engineering, Texas A&M University, College Station, Texas 77843, United States; 3 Department of Physics and Astronomy, Texas A&M University, College Station, Texas 77843, United States; 4 Center for AI and Natural Sciences, Korea Institute for Advanced Study, Seoul 02455, Republic of Korea; 5 Department of Chemistry, University of South Florida, Tampa, Florida 33620, United States; 6 Institut Pasteur, Université Paris Cité, CNRS UMR3825, Structural Bioinformatics Unit, 28 rue du Dr. Roux F-75015 Paris, France; 7 Department of Chemistry, University of Basel, Klingelbergstrasse 80, CH-4056 Basel, Switzerland; 8 Faculty of Chemistry, Department of Computational Biological Chemistry, University of Vienna, Wahringerstrasse 17, 1090 Vienna, Austria; 9 Department of Physiology and Biophysics, Case Western Reserve University, School of Medicine, Cleveland, Ohio 44106, United States; 10 Department of Chemistry, University of Michigan, Ann Arbor, Michigan 48109, United States; 11 Department of Biochemistry, University of Zürich, CH-8057 Zürich, Switzerland; 12 Institute of Bioinformatics and Systems Biology, National Yang Ming Chiao Tung University, Hsinchu 30010, Taiwan, ROC; 13 Shanghai Institute of Materia Medica, Chinese Academy of Sciences, Shanghai 201203, China; 14 Department of Biological Sciences, Lehigh University, Bethlehem, Pennsylvania 18015, United States; 15 Institute of Bioinformatics and Systems Biology, Department of Biological Science and Technology, Institute of Molecular Medicine and Bioengineering, and Center for Intelligent Drug Systems and Smart Bio-devices (IDS^2^B), National Yang Ming Chiao Tung University, Hsinchu 30010, Taiwan, ROC; 16 Renewable Resources and Enabling Sciences Center, National Renewable Energy Laboratory, Golden, Colorado 80401, United States; 17 Department of Chemistry, Boston University, 590 Commonwealth Avenue, Boston, Massachusetts 02215, United States; 18 Department of Physics, Boston University, 590 Commonwealth Avenue, Boston, Massachusetts 02215, United States; 19 Department of Biomedical Engineering, Boston University, 44 Cummington Mall, Boston, Massachusetts 02215, United States; 20 Department of Biophysics, Johns Hopkins University, Baltimore, Maryland 21218, United States; 21 Department of Physics and Astronomy, Johns Hopkins University, Baltimore, Maryland 21218, United States; 22 Laboratory of Computational Biology, National Heart Lung and Blood Institute, National Institutes of Health, Bethesda, Maryland 20892, United States; 23 Shanghai R&D Center, DP Technology, Ltd., Shanghai 201210, China; 24 Department of Chemistry, Tufts University, Medford, Massachusetts 02155, United States; 25 Department of Biochemistry and Molecular Biology, Michigan State University, East Lansing, Michigan 48824, United States; 26 School of Chemical Biology & Biotechnology, Peking University Shenzhen Graduate School, Shenzhen, Guangdong 518055, China; 27 Institute of Systems and Physical Biology, Shenzhen Bay Laboratory, Shenzhen, Guangdong 518055, China; 28 Department of Chemistry and Supercomputing Institute, University of Minnesota, Minneapolis, Minnesota 55455, United States; 29 CiTIUS Centro Singular de Investigación en Tecnoloxías Intelixentes da USC, 15705 Santiago de Compostela, Spain; 30 Schrodinger, Inc., New York, New York 10036, United States; 31 Department of Chemical and Biomolecular Engineering, University of California, Irvine, Irvine, California 92697, United States; 32 Department of Pharmaceutical Sciences, University of California, Irvine, Irvine, California 92697, United States; 33 Key Laboratory of Structural Biology of Zhejiang Province, School of Life Sciences, Westlake University, Hangzhou, Zhejiang 310024, China; 34 College of Computer Engineering, Jimei University, Xiamen 361021, China; 35 Medicine Design, Pfizer Inc., Cambridge, Massachusetts 02139, United States; 36 Department of Chemistry, Delaware State University, Dover, Delaware 19901, United States; 37 Computational Science Division, Argonne National Laboratory, Argonne, Illinois 60439, United States; 38 Department of Chemical and Biomolecular Engineering, Institute for Physical Science and Technology, Biophysics Program, University of Maryland, College Park, Maryland 20742, United States; 39 Department of Chemistry, City College of New York, New York, New York 10031, United States; 40 Disease Target Structure Research Center, Korea Research Institute of Bioscience and Biotechnology, Daejeon 34141, Republic of Korea; 41 Department of Bioinformatics, KRIBB School of Bioscience, University of Science and Technology, Daejeon 34141, Republic of Korea; 42 Department of Biochemistry, Virginia Polytechnic Institute and State University, Blacksburg, Virginia 24061, United States; 43 Department of Biotechnology and Pharmaceutical Sciences, College of Pharmacy, Western University of Health Sciences, Pomona, California 91766, United States; 44 Department of Pharmaceutical Sciences, University of Maryland School of Pharmacy, Baltimore, Maryland 21201, United States; 45 Department of Chemistry and Institute for Nanotechnology & Advanced Materials, Bar-Ilan University, Ramat-Gan 52900, Israel; 46 Department of Chemistry, Brown University, Providence, Rhode Island 02912, United States; 47 Department of Chemistry and Biochemistry, University of Texas at Arlington, Arlington, Texas 76019, United States; 48 Karolinska Institutet, Department of Biosciences and Nutrition, SE-14183 Huddinge, Sweden; 49 Harvard University, Department of Chemistry and Chemical Biology, Cambridge, Massachusetts 02138, United States; 50 Dipartimento di Fisica e Astronomia, Universitá di Bologna, Bologna 40127, Italy; 51 Borch Department of Medicinal Chemistry and Molecular Pharmacology, Purdue University, West Lafayette, Indiana 47907, United States; 52 Department of Chemistry and Chemical Biology, Indiana University Indianapolis, Indianapolis, Indiana 46202, United States; 53 School of Pharmacy, Fudan University, Shanghai 201203, China; 54 Department of Biotechnology, Indian Institute of Technology Hyderabad, Kandi, Telangana State, 502284, India; 55 Department of Chemistry, University of Chicago, Chicago, Illinois 60637, United States; 56 Department of Chemistry, Carleton University, Ottawa, Ontario K1S 5B6, Canada; 57 Eunice Kennedy Shriver National Institute of Child Health and Human Development, National Institutes of Health, Bethesda, Maryland 20892, United States; 58 Laboratoire de Chimie Biophysique, ISIS, Université de Strasbourg, 67000 Strasbourg, France

## Abstract

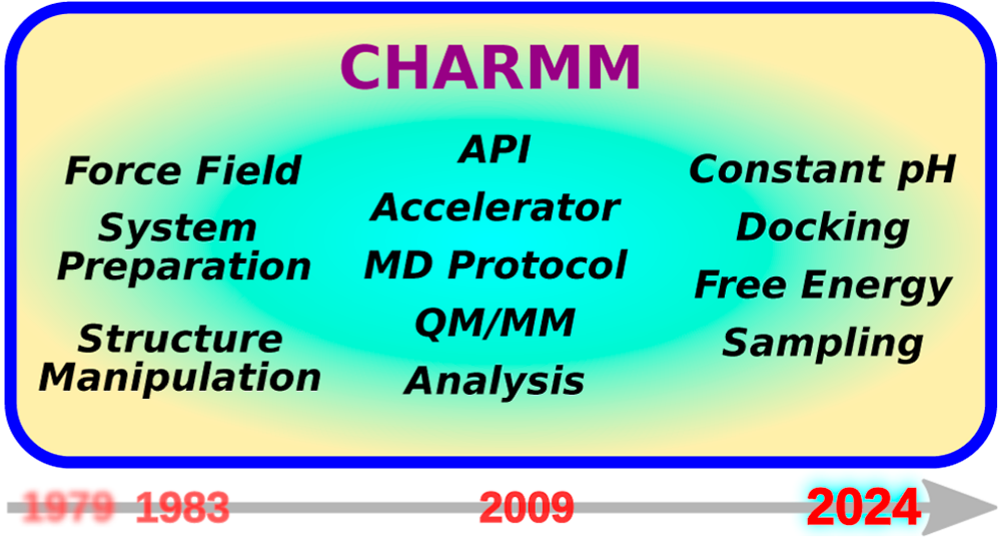

Since its inception nearly a half century ago, CHARMM has been playing a central role in computational biochemistry and biophysics. Commensurate with the developments in experimental research and advances in computer hardware, the range of methods and applicability of CHARMM have also grown. This review summarizes major developments that occurred after 2009 when the last review of CHARMM was published. They include the following: new faster simulation engines, accessible user interfaces for convenient workflows, and a vast array of simulation and analysis methods that encompass quantum mechanical, atomistic, and coarse-grained levels, as well as extensive coverage of force fields. In addition to providing the current snapshot of the CHARMM development, this review may serve as a starting point for exploring relevant theories and computational methods for tackling contemporary and emerging problems in biomolecular systems. CHARMM is freely available for academic and nonprofit research at https://academiccharmm.org/program.

## 1 Introduction

CHARMM, the program for simulation and modeling of biomolecular systems, is now more than 40 years in continuous development and use. It is freely available to academic and government laboratory users, and is fast, as we describe below due to recent advances in GPU acceleration. From its earliest incarnations as Gandalf, renamed to HARMM (Harvard Macromolecular Mechanics), and finally CHARMM (Chemistry at Harvard Macromolecular Mechanics), it has provided a working framework for the exploration of biomolecular structure–function–dynamics relationships, beginning with the first molecular dynamics (MD) simulation of the small protein pancreatic trypsin inhibitor.^1^ For an interesting perspective on the evolution of simulation methods and the applications of statistical mechanics to the study of biological molecules, we point readers to a recent review.^2^

CHARMM provides many practical and functional features that distinguish it from other programs that have evolved for similar purposes. Probably the most significant is that CHARMM was created as, and has remained, a repository for many of the trend-setting methods and models. It now comprises over 1,170,000 lines of code (modular Fortran 90, C, C++, CUDA, OpenCL, and Python) encapsulating its extensive functionality. An equally important and distinctive feature that has been integral to the software since its earliest days, is an interpreted language as its command parser, enabling “programs” to be written in “CHARMM-language” in contrast to other programs in this area whereby one prepares an input “script” describing the particular (one-pass) calculation one wishes to run. This feature greatly facilitates testing and prototyping of many of the statistical mechanical methods and techniques that have been integrated into CHARMM. While much of this has been described in earlier works and will not be further elaborated here, it is important to recognize this fundamental differentiator of CHARMM and other programs utilized in the field.

In this review, we provide an update to the developments that have occurred in CHARMM since it was last described in 2009.^3^ We refer readers to the previous two papers describing CHARMM for basic organization of the program and other functionality incorporated prior to 2009.^3,4^ Among the key new developments during the past 15 years, the most significant are the establishment of GPU-accelerated kernels to perform many of the unique calculations available in CHARMM. We describe the three published CHARMM accelerator engines in Sections 2.1 (CHARMM/OpenMM API), 2.2 (CHARMM/DOMDEC), 2.3 (CHARMM/BLaDE), and 2.4 (apoCHARMM). Each of the former three platforms are fully integrated with CHARMM and support a significant range of CHARMM functionality, and thereby provide powerful platforms for establishing complex simulation workflows utilizing CHARMM scripting language. apoCHARMM is a new GPU accelerator that is currently being developed. Except for DOMDEC, these engines provide performance comparable to any existing GPU-based biomolecular simulation code. Thus, CHARMM performance is on par with other codes while the accessibility of methods is typically richer.

Beyond the advanced simulation engines, significant efforts to integrate CHARMM into modern workflows have advanced, as described in Sections 3.1 (pyCHARMM), 3.2 (crimm), and 3.3 (CHARMM-GUI). CHARMM-GUI continues to mature and is a vital service to the community through its system and simulation setup facilities, providing input scripts for simulations for a number of current biomolecular MD simulation packages, including CHARMM, OpenMM, Amber, and GROMACS (Section 3.3). Section 3.1 on pyCHARMM describes recent efforts to use CHARMM functionality within the context of Python language. This enables the straightforward integration of CHARMM and the immense base of Python modules. Finally, crimm is a Python-based package that integrates many simulation preparation tasks, e.g., building of missing residues and loops, choosing protonation states appropriate for a given pH, patching to represent disulfide and other system modifications and solvation. These tools and methods of CHARMM provide an essential platform for modern simulation and modeling workflows.

New docking methods and procedures are described in Section 4, followed by an update of the free energy (Section 5) and constant pH (Section 6) methods. We then discuss the new enhanced sampling and transition path methods (Section 7). CHARMM has supported a range of reactive, implicit and coarse-grained models for simulation of biorelated systems throughout its history and new developments are discussed in Section 8. System-specific and specialized restraint methods that have been developed and implemented in CHARMM recently are in Section 9.

CHARMM’s force fields (FFs) are corner posts of molecular simulations throughout the field. Section 10 gives updates to the CHARMM fixed-charge and polarizable FFs. Following is a discussion of the recent approaches and methods for quantum mechanics and molecular mechanics (QM/MM) simulations that are integrated into CHARMM (see Section 11). Lastly, we describe new methods, procedures and analysis tools that have been integrated into CHARMM in Section 12.

Through this review, we hope to convey the immense base of methods and models that are available and supported in CHARMM. Additionally, we hope that readers will use this review as an entry to the growing online repositories for tutorials, advanced simulation methods, and templates.

## 2 CHARMM Accelerator Engines

Since the previous review of CHARMM functionality,^3^ there has been a significant growth in utilizing new generation of processors, especially GPUs, to accelerate MD and related modeling tasks within biomolecular simulation methods. The CHARMM development community has embraced this effort by developing specialized GPU accelerator and highly parallel kernels, or adaptor APIs that support a range of the extensive functionality available in CHARMM, from free energy methods and constant pH simulation techniques, to implicit solvent models such as generalized Born (GB) models and a host of others. These interfaces provide a straightforward means to set up systems, manipulate, patch or otherwise prepare them for simulation and then simulate using GPU or parallel CPU execution without creating extraneous intermediate files. This seamless interface also enables straightforward analysis and visualization of results from within the same pyCHARMM/CHARMM script, thereby providing an integrated framework for modeling, dynamics, and analysis. We present below in chronological order, the accelerated performance platforms that are integrated into CHARMM. As indicated in Table 1, the performance of CHARMM through the GPU-accelerated APIs compares well with those observed in other GPU-accelerated packages such as pmemd.cuda,^5^ GROMACS,^6^ and NAMD.^7^ CHARMM/pyCHARMM (Section 3.1) also offers a range of developed and developing interfaces to meet needs across a breadth of the methodological application areas.

**Table 1:** CHARMM Accelerated Engine Benchmarks on GPUs (ns/day; avg ± sd over 5 runs)^a^

	System	DHFR	APOA1	DMPG	T4L (*N*_*s*_ = 6)	HSP90 (*N*_*s*_ = 11)
GPU	Atoms	23558	92224	291168	37723	26685
Ada RTX6000	BLaDE	601.6 ± 2.4	255.6 ± 1.2	82.1 ± 0.4	-	-
	OpenMM	1052.0 ± 3.5	257.8 ± 2.8	78.0 ± 0.9	-	-
	apoCHARMM	423.5 ± 1.3	289.2 ± 1.4	95.3 ± 2.5	-	-
Ada RTX5000	BLaDE	514.3 ± 6.0	191.7 ± 0.6	63.6 ± 0.2	-	-
	OpenMM	897.3 ± 1.1	197.7 ± 2.3	62.7 ± 1.3	-	-
	apoCHARMM	323.6 ± 11.5	218.3 ± 0.7	72.3 ± 0.3	-	-
Ada RTX4500	BLaDE	436.7 ± 0.45	142.9 ± 0.2	45.6 ± 0.04	-	-
	OpenMM	730.0 ± 0.3	138.9 ± 1.2	43.6 ± 0.6	-	-
	apoCHARMM	388.6 ± 0.7	152.2 ± 0.7	51.3 ± 0.2	-	-
RTX A6000	BLaDE	355.9 ± 2.7	124.5 ± 0.2	40.3 ± 0.4	-	-
	OpenMM	656.0 ± 6.5	129.8 ± 1.6	39.5 ± 0.5	-	-
	apoCHARMM	295.8 ± 0.7	133.8 ± 1.1	42.6 ± 0.3	-	-
RTX A5500	BLaDE	333.5 ± 1.0	111.8 ± 4.7	37.9 ± 0.3	-	-
	OpenMM	594.3 ± 2.7	113.4 ± 0.6	31.2 ± 0.1	-	-
	apoCHARMM	277.2 ± 5.6	135.3 ± 2.4	38.5 ± 0.2	-	-
A100	BLaDE	402.9 ± 4.7	149.4 ± 0.9	54.0 ± 0.2	208.8 ± 0.3	246.8 ± 0.4
	DOMDEC	106.3 ± 0.7	-	9.07 ± 0.06	33.5 ± 0.0	46.4 ± 0.3
	OpenMM	558.5 ± 8.6	121.7 ± 2.3	36.2 ± 0.6	-	-
	apoCHARMM	276.1 ± 1.5	172.6 ± 1.3	63.6 ± 0.3	-	-

a DHFR, APOA1, and DMPG are for NVT simulations with 9-Å real-space non-bonded cutoff distance. T4L and HSP90 are for *λ*-dynamics in NPT ensemble and with 10-Å real-space non-bonded cutoff distance. *N*_*s*_ in parentheses is the number of *λ* variables used (*cf.*, Eq. 4). PME was used to account for long-range electrostatic interactions.^24^ In all simulations, the integration time step was 2 fs.

### 2.1 The OpenMM API

The first GPU-accelerated engine coupled to CHARMM comprised a FORTRAN-90 API that takes advantage of the significant developments of OpenMM.^8^ This interface provides direct calls to OpenMM functionality for MD, energy minimization, and free energy methods. A host of restraints existing in CHARMM are also implemented using OpenMM’s custom forces.^8^ This effort, spearheaded by Michael Garrahan and Charles Brooks, first appeared in CHARMM release c37b1. A key advantage of using OpenMM through the CHARMM/OpenMM API is that applications that need to move between system setup and preparation, processing, and analysis can occur within a single workflow using the CHARMM interpreted command language or directly though Python with pyCHARMM.^9^ Aside from the many simulation environments, restraints, heavy atom-hydrogen constraints, NVT and NPT (using either an isotropic Monte-Carlo barostat or anisotropic pressure coupling often used in membrane simulations), particle-mesh Ewald (PME), CHARMM shifting and switching methods for van der Waals and/or electrostatic interactions, this interface also supports free energy perturbation (FEP) methods utilizing fixed windowing approaches, e.g., FEP with analysis by MBAR (Section 11.11).^10−12^ The CHARMM/OpenMM API provides a robust platform for integrated modeling tasks and workflows and has been utilized in the extension of the CDOCKER approaches^13−15^ to parallel simulated annealing with GPU acceleration with any of the CHARMM-compatible physically based FFs, including CHARMM,^16^ CHARMM General FF (CGenFF),^17,18^ AMBER,^19^ GAFF,^20^ OPLS,^21^ and the LigParGen OPLS extension for small molecules.^22^ At present, not all of the CHARMM functionalities are fully implemented through the API, and a key missing element is full access to the Drude polarizable FF that has been rapidly developing over the past several years.^23^ Plans are underway to provide availability through the CHARMM/OpenMM API in the near future.

### 2.2 DOMDEC Parallel-Scalable Platform

In 2014, a new DOMain DEComposition (DOMDEC) MD engine was introduced into CHARMM by Hynninen and Crowley.^25^ It was faster both in the execution on serial and parallel CPU platforms. Serial performance was approximately two times higher than in the previous versions of CHARMM with its “fast” CPU-based options. The parallel version enabled efficient utilization up to hundreds of CPU cores.

The DOMDEC module of CHARMM served as an early platform for the development of the multisite λ-dynamics (MSλD)^26−28^ and explicit-solvent constant pH MD (CpHMD)^29−31^ methods. Also implemented as part of this effort were GPU-resident kernels that accelerated components of the computation and enabled partitioning of the system being studied across both CPU and GPU cores for scaling and acceleration. Finally, a GPU implementation that handles all energy calculations except for the SHAKE constraint and position propagation was implemented in the “GPU only” functionality of DOMDEC. All of CHARMM’s MSλD and CpHMD functions were integrated into the “GPU-only” kernel, which was the platform on which NAMD’s GPU-accelerated kernel (gpu-offload) was based as well as more recent faster engines including the newly developing apoCHARMM (Section 2.4).

### 2.3 BLaDE

The BLaDE (Basic LAmbda Dynamics Engine) module of CHARMM^24^ was developed to optimize the speed of λ-dynamics simulations on GPUs, but it also provides a robust and accelerated platform for conventional MD simulations. Previously, the DOMDEC module^25^ was the fastest implementation of λ-dynamics as noted above, but the SHAKE constraint and position propagation being handled by the CPU were rate limiting. Also, DOMDEC performed suboptimally on smaller systems on a single GPU. BLaDE optimizes these tasks and achieves 5- to 6-fold speedup over DOMDEC. Although the CHARMM/OpenMM API (discussed above) exhibits similar performance on standard MD (Table 1), it is less suited for λ-dynamics.

Table 1 shows benchmarks for BLaDE, DOMDEC, and OpenMM through their CHARMM interfaces, and a stand-alone version of apoCHARMM. We have focused on previously established benchmarks as presented earlier^24^ and elsewhere. DHFR is a small globular protein, ApoA1 is a solvated lipid nanodisc, DMPG is a larger lipid bilayer. T4L is a protein mutation calculation, and HSP90 is a ligand perturbation calculation. Benchmarks were repeated 5 times and run on single NVIDIA GPUs as noted in Table 1. These benchmarks demonstrate that BLaDE scales well, especially for λ-dynamics.

Since BLaDE is designed to be simple and fast, not all features present in CHARMM are available in BLaDE. For example, while much of CHARMM uses the Langevin Piston barostat,^32^ BLaDE uses the Monte Carlo barostat^33,34^ in constant pressure simulations, removing the overhead of computing the virial and rectifying SHAKE constraints after coordinate update. Similarly, BLaDE only includes a Langevin thermostat, and is not yet implemented in energy minimization routines, though energy calls can be made directly to BLaDE. New features continue to be added, including support for harmonic and nuclear Overhauser effect (NOE) restraints, support for non-orthogonal boxes, PME^35^ or force/energy switching electrostatics,^36^ and support for AMBER FF with different 1–4 scaling and improper torsion potentials.

### 2.4 apoCHARMM, Embracing CHARMM-Centric Functionality

apoCHARMM is a developing open source package designed specifically to support some of the distinctive methods of CHARMM absent in the CHARMM/OpenMM or CHARMM/BLaDe APIs (see Sections 2.1 and 2.3), and at the speeds provided by modern GPU architectures (Table 1). In particular, apoCHARMM in its current development supports, or plans to support:

- A complete analytic virial tensor.
- Multiple PSFs (protein structure files) simultaneously (upper limit set by the hardware resource).
- Uncommon crystal symmetries such as P2_1_ (Section 12.4).

By accounting for the complete virial tensor, an implementation of the Langevin piston algorithm^32^ for constant pressure or constant surface tension ensembles is enabled. Simultaneous support for multiple PSFs allows free energy methods modeled after the CHARMM PERT approach to be run. It also allows the enveloping distribution sampling (EDS) based method for free energies,^37−39^ and for state-based CpHMD.^40,41^ Support for P2_1_ crystal symmetry^42,43^ allows lipid bilayer systems to be simulated without chemical potential mismatch between upper and lower leaflets, which can be very useful when making membrane insertions^44^ (Section 12.4).

A number of different integrators have been implemented for different ensembles: Velocity-Verlet and leapfrog integrators for the microcanonical ensemble, and Langevin thermostat and Nosé–Hoover integrators for the canonical ensemble. Holonomic constraints are handled using SHAKE and SETTLE algorithms. Since the virial is calculated during force calculation, the isobaric ensemble can be sampled using the Langevin piston method.^32^ This is an extended ensemble method with additional degrees of freedom corresponding to pistons that are used to control the pressure. Thus, a number of ensembles are available including constant area (NPAT) and constant surface tension (NPγT).

Several methods for free energy difference calculations are implemented in apoCHARMM.^45^ A unifying scheme, which depends on a variant of energy interpolation, is implemented using a composite design pattern, where forces and energies of the end states after being separately calculated are interpolated. Additionally, soft-core formulation of the van der Waals interaction^46^ to calculate λ-specific energy is available. The double exponential method^47^ has been implemented as well. Although it is slightly slower than the van der Waals formulation, it provides a base version of soft-core.

apoCHARMM is derived from the erstwhile GitHub package by Antti-Pekka Hynninen.^48^ It is written in CUDA and modern C++ to leverage the full potential of NVIDIA GPU architectures. Additionally, it features a Pybind11-based Python interface, ensuring convenience for end-users. The codebase adheres to test driven development (TDD) principles and incorporates Catch2-based unit tests with extensive code coverage. Notably, apoCHARMM is designed as a GPU-exclusive implementation, with all aspects of MD including energy and force calculations, restraints, constraints, and integration, executed entirely on GPU. Minimizing host-GPU memory transfers, the system only necessitates such transfers during logging or trajectory saving operations. One of the modular design patterns employed is the mediator pattern that reduces dependencies between different components by mandating communication through a central mediator object. In a similar vein, loggers and integrators leverage a publisher–subscriber design pattern, facilitating the versatile reuse of different loggers with distinct integrators. Overall, apoCHARMM performance is comparable to or better than other GPU based MD engines (Table 1). Since it is optimized for larger systems, its performance is not as good for the smaller DHFR.

## 3 Streamlining CHARMM Workflows

The rich methodology and broad functionality of CHARMM, including its unique scripting language, has enabled many complex workflows to be created and tested prior to committing them to code in FORTRAN 90/C/C++.^3^ These scripting capabilities have led to extensive libraries of CHARMM scripts in various forums and repositories as well as seeded the establishment of the web-based CHARMM-GUI^49^ and a range of other modeling tasks, e.g, MCSS,^50^ early stages of XPLOR,^51^ and SILCS.^52^ CHARMM scripting language, although extremely powerful, is not naturally integrable with other workflows that are convenient and widely used in the modeling of biomolecules. This realization has provided impetus for establishing a complete Python interface to CHARMM’s full range of functionality and efforts to facilitate the utilization of CHARMM and pyCHARMM, namely crimm and CHARMM-GUI.

### 3.1 pyCHARMM

Efforts were initiated in the group of Charles Brooks to develop CHARMM callable functionality though a Python interface and APIs, called pyCHARMM.^9^ It enables CHARMM variables and data structures to be explored and used, and in some instances manipulated at the Python level, providing the means of creating complex workflows that integrate and extend tools built in Python for numerical and graphical tasks. Native Python functions and modules complement and extend the already rich landscape of CHARMM functionalities. Examples include a framework that enables novel energy functions to be integrated with CHARMM’s modeling tools through Python callable routines available in Python, CUDA, and OpenCL, as well as utilize machine learned functions such as TORCH-ANI^53^ and PhysNet^54^ for energy and force calculation. Analogous ‘hooks’ are built into the CHARMM dynamics engine. Graphical engines are also readily integrated into pyCHARMM for rapid visualization of simulation models and results. Loosely coupling tasks across many processors too is straightforward within pyCHARMM workflows using MPI frameworks such as MPI4PY and this has facilitated free energy calculations using multiscale Bennett’s acceptance ratio (MBAR) and thermodynamic integration (TI) approaches, or high-throughput MSλD free energy methods (Section 5.1), string path optimization calculations (Section 7.3), replica exchange (Section 7.1), and fully automated docking workflows employing CDOCKER (Section 4.1).^13,14,55,56^ pyCHARMM is integrated with the accelerated platform kernels and APIs of CHARMM/OpenMM (Section 2.1) and CHARMM/BLaDE (Section 2.3).

Since its release in early 2023,^9^ two workshops have been given on integrating modeling tasks using pyCHARMM. The first focus was on general modeling tasks and methods with examples provided as Jupyter Notebooks and Python scripts (July 2022),^57^ followed by an advanced workshop held in July 2023, focused on utilizing pyCHARMM for high-accuracy, high-throughput free energy calculations.^58^ The Jupyter Notebooks and scripts associated with both workshops are also available through the GitHub page of Charles Brooks’ lab: https://github.com/BrooksResearchGroup-UM. The release and ongoing development of pyCHARMM represent an important milestone for integrating biomolecular modeling, FFs, advanced simulation, sampling, and docking protocols, into the widely used Python programming language.

### 3.2 crimm

Despite the best efforts from the developers of major biomolecular modeling and simulation softwares, there exists a substantial barrier when researchers first start to learn these tools. Frustrations often arise from the unfamiliarity of command scripts and in system preparation protocols that involve multiple steps to process macromolecules, small molecule ligands, water, and ions. A number of structural preparation tools have been developed for this purpose. For example, CHARMM-GUI (Section 3.3), originally designed to prepare simulation systems for CHARMM, provides a web interface to assist users with building a system for simulation.^49,59−62^ Despite convenience, it lacks scriptability and integratability. Other tools such as PDBfixer from OpenMM provide Python APIs for scripting and possible integration with other tools. However, they rely on structure files such as PDB as an intermediary to pass structural information to other software packages. The limitation of the PDB file format has rendered it insufficient in keeping complete information of a macromolecular system. Python packages such as Biotite^63^ and BioPython^64^ offer adequate APIs for structure manipulation and protocols for integration with other computational tools, but they are limited in utility for structural preparation for MD simulations. Functions such as building missing loop regions, building missing atoms, adding hydrogens, solvation, *etc.,* are currently absent. To address issues of scriptability and integratability in structure preparation, crimm (Chemistry with ReInvented Macromolecular Mechanics)^65^ is being designed with the following software principles and aims:

1. Accurate, consistent, and complete structure information and annotations for biomolecules maintained throughout the structure preparation pipeline.
2. Intuitive object design that organizes structural entities (e.g., model, chain, residue, atom) for retrieving information and manipulating structures, thus providing greater flexibility for programming.
3. Abstraction on routines (e.g., protonation, solvation, loop building) to create high-level APIs provided in Python for a convenient and intuitive scripting on structure preparation.
4. Clear protocols and reference implementations to Adaptors (interfaces to convert between Python classes in memory) to pass structural information to other software library or platforms, where accurate and efficient transfer of data can be guaranteed.
5. Visualizations in an interactive programming environment, i.e., Jupyter Notebook, to aid examination of structures.
6. Ease of installation, free and open source, and support for all major hardware platforms to encourage adaptation.

Crimm is directly built on the BioPython library and adopts the SMCRA model (Structure, Model, Chain, Residue, and Atom) for representing structures.^64^ A BioPython-based object class provides optimal classification of macromolecular chain entities (protein, RNA, DNA, oligosaccharide, *etc.*). Importantly, functions of BioPython can be directly called with crimm structural object as an argument. All structural objects can be directly visualized using NGLView^66^ in a Jupyter Notebook/Lab.

Structure preparation in crimm begins by fetching structures from the RCSB^67^ or AlphaFold DataBase^68^ as mmCIF format files for the complete and consistent organization of information.^69^ In the context of CHARMM, the topology generation functions with CHARMM naming conventions and the CHARMM C36 FF is used. Currently available routines to process initial structures from the RCSB include automated missing loop/residue and disulfide bond assignment based on the data in the mmCIF file, patching of titratable residues with protonation state assigned by using interface to PropKa,^70^ and topology generation. A solvation module is under development. Adapters to pyCHARMM^9^ have been implemented and crimm structures can be operated on or one can run simulations with CHARMM functions via pyCHARMM. An Adapter to RDKit^71^ has also been implemented for small molecule ligands integral to PDB entries. These are created as mol objects in RDKit to guarantee they maintain the correct bond orders. Other features are currently being developed to aid structure preparation and will address interfaces and adaptors to packages such as OpenMM,^8^ OpenFF,^72^ and Autodock Vina.^73^

### 3.3 CHARMM-GUI

Since its original development in 2006,^49,59−62^ CHARMM-GUI has proven to be an ideal web-based platform (https://www.charmm-gui.org) to interactively build complex systems and prepare their inputs with well-established and reproducible simulation protocols for widely used simulation packages such as CHARMM, AMBER,^74^ Desmond,^75^ GENESIS,^76^ GROMACS,^77^ LAMMPS,^78^ NAMD,^79^ OpenMM,^8^ and Tinker.^80^ CHARMM-GUI has been widely adopted for various purposes and it now contains more than 20 modules designed to set up a broad range of molecular simulation systems.^81−83^ CHARMM-GUI also provides educational resources including online lecture materials, an online user forum, and workshops. Its archives support scientific reproducibility by providing the lipid conformation library^59,61,62^ used in membrane generation, prebuilt COVID-19 systems,^81,84,85^ prebuilt membrane complexes,^82,83^ and a searchable CHARMM small molecule library (CSML). Many original modules were developed as an in-house effort, but close collaborations with the developers of CHARMM and other simulation packages have been established for adding newer modules.^86−88^

The philosophy in CHARMM-GUI development is less about providing the nuts and bolts of molecular modeling, but instead it focuses on helping users to achieve a task, including building membrane systems,^61,62,89−94^ modifying and solvating proteins,^95,96^ characterizing protein–ligand interactions,^97−104^ or modeling complex carbohydrates^105−107^ via a streamlined interface.^108−116^ This makes CHARMM-GUI broadly accessible to users with little experience in modeling tools while remaining useful to experts, especially for batch generation of systems.

CHARMM-GUI development is not only guided by requests from general users and experts, but also in response to an emerging need for a unified platform to prepare and execute various advanced simulation approaches developed in diverse simulation communities and packages.^113,117−120^ In addition to building complex molecular systems, CHARMM-GUI also assists with preparing input files for both general and advanced modeling and simulation tasks.

## 4 Docking Methods

### 4.1 CDOCKER

First introduced in 2003,^55^ CDOCKER provides an integrated CHARMM-based scripting framework for small molecule-receptor docking studies. It employs a numerical grid-based representation for the van der Waals and electrostatic interactions utilizing a fully molecular mechanics (MM)-based FF representation of the interactions.^55^ CDOCKER utilizes conformational search based on simulated annealing, and it is also compatible with enhanced sampling search approaches such as self-guided Langevin dynamics.^58^ It has been used in a broad range of applications, including early efforts in community-based docking.^121−125^ In this capacity it served as a platform to explore a range of docking and scoring approaches, including some of the early flexible receptor^126^ and covalent docking methods.^56^

In the past few years, CDOCKER has been significantly updated to utilize accelerated platforms such as GPUs.^13−15^ While the basic philosophy has remained centered on sampling via (accelerated) simulated annealing and structured around the state-of-the-art small molecule and biomacromolecular FFs, new fast Fourier transform (FFT)-based approaches have been introduced for binding pocket and ligandable-site discovery via functional probe docking,^127^ representing important hydrogen bonding by use of hydrogen-bond-specific donor–acceptor grids,^14^ and hybrid sampling methods that combine simulated annealing with genetic algorithm moves.^14^

CDOCKER has also been implemented as a package within pyCHARMM (Section 3.1). In addition to providing full access to the methods available within CDOCKER, pyCHARMM greatly simplifies the workflow through use of ‘best practice’ parameter choices and a single callable pyCHARMM command. This enables large-scale virtual screening via a single script that integrates ligand building via RDKit and SMILES strings, parametrization of ligands with small molecule FF parameter estimators such as CGenFF,^17,18^ GAFF,^20^ LigParGen,^22^ and OpenFF,^128^ automated protein grid generation, parallel docking, clustering of results and ranking, including reranking with implicit solvent models such as GBSA/GBSW/GBMV, and FACTS.^129−132^ In summary, CDOCKER is a fast, flexible and accurate GPU-accelerated molecular docking engine that can handle cases from high-throughput small probe docking to flexible receptor–ligand docking.

### 4.2 EnzyDock

**Figure 1:**
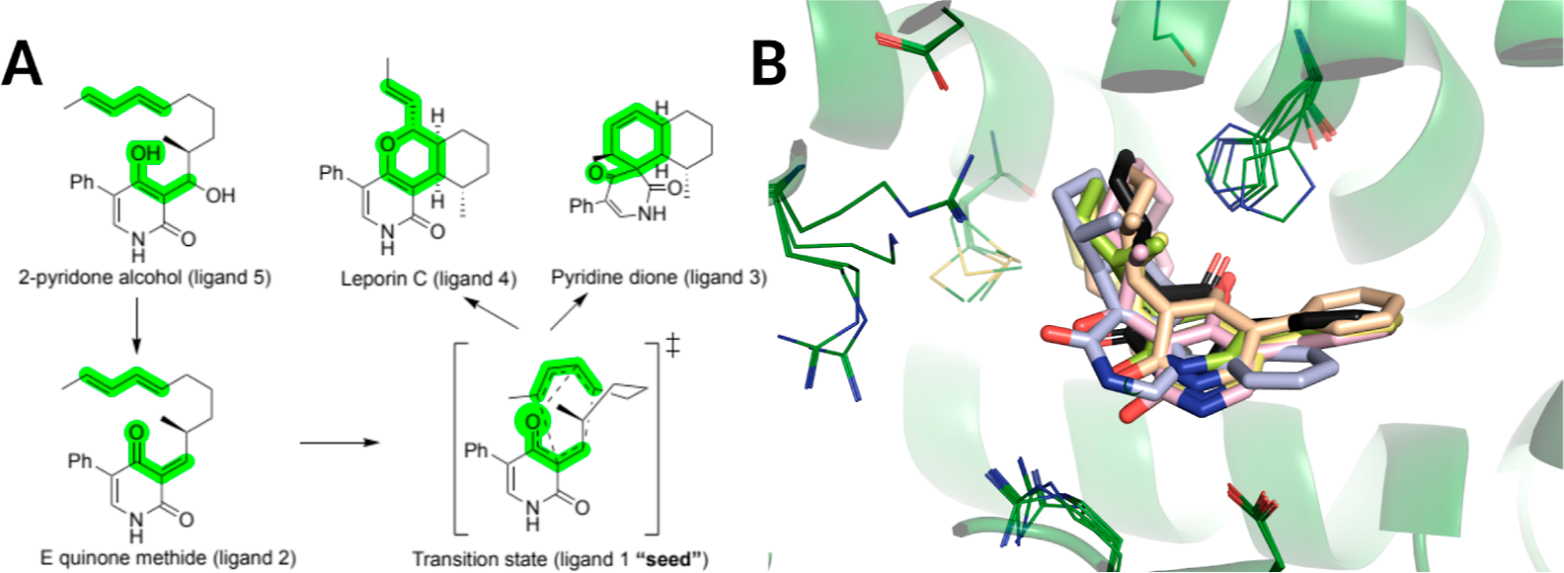
Main concepts of EnzyDock applied to the mechanism in the Diels–Alderase enzyme, LepI.^133,134^ (A) Similar (mapped) atoms are marked in green. (B) EnzyDock docking with the transition state as a template (“seed”) for docking the remaining states.

Modeling enzyme reactions requires a carefully designed computational protocol that relies on well-established theoretical foundation. The starting point is reliable 3-dimensional (3D) structures of the substrate, product, intermediates, or transition states bound to the enzyme. EnzyDock^135^ is a CHARMM-based docking program like the well-known CDOCKER^56,136,137^ (Section 4.1), with emphasis on enzymes. Its main feature is mechanism-based multistate consensus docking that allows the docking of reaction substrate, intermediates, transition states, and products in a mechanistically consistent and induced-fit manner (Figure 1). EnzyDock is written as a series of CHARMM scripts (>10,000 lines of script code), Python codes (∼3,000 lines), and shell scripts. EnzyDock is a docking-tool and it does not compute free energy profiles that can be obtained using other methods in CHARMM such as umbrella sampling (US),^138^ string-based methods^139^ (Section 7.3), or metadynamics.^140,141^ Consensus docking in EnzyDock is achieved by applying geometric restraints implemented via NOE restraints on reaction states relative to a predetermined “seed” state, such that all states are docked with similar poses under a given user-defined threshold (Figure 1). For instance, the seed state could be a tightly bound transition state or a known inhibitor-bound state. Conversely, if unrestrained multistate docking is performed, a reaction pathfinder module identifies all matching poses along a reaction path.^142^ Additional restraints such as on dihedral angles can enforce specific stereo- and regio-chemistry during docking, while positional harmonic and NOE restraints can be employed to include chemical information such as the initial cleavage site, nucleophilic attack, or ligand positions relative to key active-site residues or cofactors. Different protonation states of enzyme and cofactors during docking of different states is facilitated via CHARMM patching. Sampling of configurational space is performed using MD^3^ or Monte Carlo (MC)^143^ simulated annealing on a grid representing the enzyme,^55^ and poses are scored using the C36^144^ and CGenFF^145^ FFs. Flexible residues, cofactors, and waters are treated as explicit atoms on the grid. Following ligand pose clustering, final energy minimization and scoring is performed using all-atom description of the entire system and optional refinement using a QM/MM approach^135,146^ with a range of QM methods, e.g., semiempirical (SE)^147^ or density functional theory (DFT)^148^ (Section 11). Bulk solvation is modeled using an implicit solvation model (e.g., GB).^3^

EnzyDock has been applied to diverse systems such as terpene synthases, racemases, Diels–Alderases, phosphotriesterase,^149^ and covalently bound ligands.^135,150^ It has also been used along with other docking programs in a benchmark study on ligand binding in the main protease in SARS-CoV-2.^151^

From a user perspective, the enzyme must be provided as a PDB file or CHARMM PSF and CRD (coordinate) files, ligand states as PDB files or SMILES strings, and atom mapping between similar states along a reaction path must be provided by the user. Additional restraints can be provided by the user. EnzyDock is available via GitHub and has recently been implemented in CHARMM-GUI^49^ (Section 3.3).

### 4.3 CIFDock

Accurately modeling protein and ligand flexibility is vital when using molecular docking to elucidate binding modes and predict binding affinity. Binding events may rely on “induced fit” where the ligand induces conformational changes in the protein binding site.^152^ Accounting for induced fit has been shown to be critical for accurate modeling of the complex.^153,154^ To this end, we developed a novel CHARMM-based induced fit docking protocol (CIFDock)^155^ that employs all-atom FFs and enhanced sampling MD.

The CIFDock protocol begins with processing the protein structure through CHARMM-GUI^96^ to fix bond orders, add hydrogens, and correct protonation states of the protein residues. The resulting PDB file is then fed into a series of CHARMM scripts which separate the protein, ligand, ion, and water molecules into CHARMM-compatible structure files, and they will be combined during subsequent steps. Key to the CIFDock protocol is the definition of active site residues that are mutated to alanine, to allow for a more “open” active site that can accommodate larger ligands and facilitate greater ligand conformational searching. In the final preparation step, the Confab module of OpenBabel^156^ is used to generate a ligand conformational ensemble to seed initial binding pose searching.

The main docking procedure begins with the initial placement of the ligand in the active site of the protein in a random orientation. The ligands are then sampled using a 20-ps self-guided Langevin dynamics (SGLD)^157^ simulation. Following this step, pairwise root-mean-square deviation (RMSD) clustering of ligand conformations is performed using the CORREL module to avoid further sampling of overlapping conformations. Each cluster is saved as a trajectory file which consists of conformations that were within a predefined cutoff radius of the cluster center. Each of the resulting protein–ligand complexes is then “backmutated” (i.e., the residues mutated to Ala are mutated back to their original residues) and a random dihedral angle-based rotamer library^158^ is generated, and side chains are relaxed by a short energy minimization and SGLD simulation. Explicit water molecules and ions saved in the preparation stage are added back, and a second SGLD simulation is conducted on the active site complex.

The resulting “docked” poses are scored and ranked using a set of custom scoring functions that are based on the well-validated SWISSDOCK^159^ scoring function. They are linear combinations of energy terms calculated by CHARMM, which include FF-based energies and the GMBV II implicit solvent model for solvation energy.^160^ CIFDock was validated by cross-docking studies on a set of 21 pharmaceutically relevant proteins. Results obtained were comparable to, or in some cases improved upon, commercial docking programs. This can be attributed to the treatment of the ligand, active site, and explicit waters as fully flexible components during the docking procedure. Additionally, because CIFDock is based on short classical MD simulations, its computational cost is minimal.

To handle the formation of covalent bonds and allow covalent inhibitors to be screened, we integrated both MNDO and SCC-DFTB^161−165^ minimizations (Section 11) into the CIFDock workflow. These minimizations together with additional dynamics simulations using positional restraints ensure adequate protein–ligand complex sampling pre- and postreaction. The covalent-based CIFDock (CovCIFDock) has been validated on a cross-dock and self-dock test set,^166,167^ with an average RMSD of 1.91 and 1.89 Å, respectively, and a 76% success rate. This compares favorably with commercial covalent docking programs such as Schrödinger’s CovDock-LO (Lead Optimization) that has a 74% success rate on the same test sets. The hybrid QM/MM minimizations also add little computational overhead to the docking procedure.

## 5 Free Energy Methods

### 5.1 λ-Dynamics, Multisite λ-Dynamics, and Constant pH MD

Alchemical free energy simulations are an important class of statistical mechanical methods used in computing free energy values and differences in small molecule design and refinement,^28,168,169^ as well as protein design^170,171^ and CpHMD simulations.^50,172^ Alchemical methods determine free energy differences by simulating chemical transformations along a non-physical pathway, often using a chemical progress variable λ. λ-dynamics is a particularly efficient and scalable alchemical method that takes advantage of natural fluctuations in the systems being studied to “drive” the chemical coordinate between the desired end points, and is generalizable to multidimensional chemical spaces, allowing exploration of many substituents at a site or even at multiple sites (MSλD) in a single simulation.^26,173^

For two states *A* and *B* of a molecular species (e.g., a protein or a side chain) and the environment (e.g., solvent and/or the receptor pocket), the alchemical hybrid Hamiltonian (or Lagrangian) for the λ-dynamics is
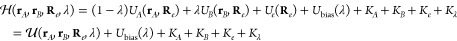
1where *U*_*A*/*B*_ represent potential energy of *A*/*B* interacting with themselves and the environment and *U*_*e*_ is the potential energy of the environment itself. Terms involving these three potential energies are denoted together as . *U*_*bias*_ is a biasing (umbrella) potential to facilitate sampling in the chemical coordinate λ. *K*_*p*_ (*p* ∈ {*A*, *B*, *e*, λ}) is the corresponding kinetic energy term for the conformational or chemical variable. From Eq. 1, one can derive coupled equations of motion for the atomic coordinates **r**_*A*/*B*_ and **R**_*e*_ and the chemical coordinate λ with a suitably assigned mass. Integrating the equations of motion subject to a holonomic constraint on λ ∈ [0, 1] allows sampling of the “extended system” in a statistical ensemble of choice.^173^ In the canonical ensemble, the partition function is
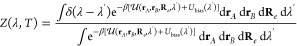
2where δ(λ – λ′) is the Dirac-δ function and β = 1/*k*_*B*_*T* is the inverse temperature (*k*_*B*_: Boltzmann constant, *T*: temperature). It follows that Δ*G*_*AB*_ is given by
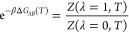
3

Extension to multiple sites at one or multiple positions of a scaffold is generalized from the terms in  above to
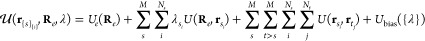
4Thus, each substituent *i* of the *N*_*s*_ substituents at each site s of the *M* total sites gets its own *λ*_*s*_*i*__. Interactions of a substituent with itself and the environment *U*(**R**_*e*_, **r**_*s*_*i*__) are scaled by *λ*_*s*_*i*__ while interactions between sites *U*(**r**_*s*_*i*__, **r**_*t*_*j*__) are scaled by the product of *λ*_*s*_*i*__*λ*_*t*_*j*__, and all remaining interactions, *U*_*e*_(**R**_*e*_), are unscaled. Although λ-dynamics has been primarily implemented in CHARMM, it can be implemented in OpenMM using custom non-bonded forces.^8^ However, for large chemical spaces the computational efficiency is poor. CpHMD methods based on λ-dynamics have also been implemented in GROMACS,^6^ Amber,^5^ and AMOEBA.^174^

In CHARMM, λ-dynamics is implemented through the BLOCK module, where many new features have been introduced to improve the accuracy, robustness, scope, and sampling. It is computationally expedient to ensure that λ remains between 0 and 1 (boundary constraint), and all λ values at a particular site add up to 1 (normalization). While these criteria can be maintained approximately or exactly with restraints or constraints, respectively, as was done in the earliest implementations,^173^ it is more convenient to maintain them with implicit constraints through change of variables. This provides an alternative set of alchemical variables θ that map back to λ such that the constraints and normalization are satisfied by construction.^26^

Soft-core interactions that remove non-bonded singularities near the alchemical end points of 0 or 1 are important for convergence. They are especially critical for the accuracy and reproducibility of λ-dynamics because free energy is estimated by binning together states near alchemical end points, where hard cores can lead to very sharp changes in the free energy. The BLOCK module contains a special set of soft core functions for λ-dynamics that enables van der Waals and electrostatic interactions to be turned off concurrently.^175^ For λ-dynamics, the PME electrostatics^35,176^ gives better results than force switching electrostatics,^36^ especially for longer simulations.^177,178^ The BLOCK module includes commands to enable a generalization of PME for λ dynamics.^179^ It is also worth noting that the MSLD command (‘L’ for λ) in the BLOCK module accepts an FFIX option that will run otherwise identical simulations, but with fixed values of λ for FEP validation or discrete λ sampling.^10,180−182^ Several additions to BLOCK allow broader applicability of λ-dynamics to more unusual perturbations. Protein mutations to proline, and ligand calculations involving ring changes, core hopping, or macrocyclization require special considerations to ensure that when a substituent is non-interacting at λ = 0, the dummy atoms in the substituent are only bonded to one environment atom so they do not exert a net force on the rest of the system.^183^ To satisfy these considerations, the BLOCK RMLA command allows removing λ scaling for classes of interactions, and it is recommended to only leave bond and angle interactions unscaled and to scale dihedrals. For finer granularity, soft bonds are implemented in BLOCK to, for example, break the proline ring at λ = 0 and allow free rotation of other amino acids at the same site around their λ backbone angle.^184,185^ If significant portions of a molecule are similar but cannot be incorporated into the common core of a hybrid topology model due to differing charge or atom types, they may be harmonically restrained together with their bonded interactions scaled with the CATS command in BLOCK,^185^ analogous to a similar process in NAMD.^7^

Another set of features crucial for sampling of the chemical space is adaptive landscape flattening (ALF) where a biasing potential in the λ space is iteratively developed to flatten the chemical landscape for enhanced sampling.^175,177,178^ These biases are implemented by the LDIN and LDBV commands and are typically tuned by an external ALF python package.^175^ Sampling can also be improved with Hamiltonian replica-exchange MD (REMD) through the REPD module.^27,31^ More rapid sampling can be achieved with the BLaDE module^24^ (Section 2.3).

The above developments enabled sampling of massive chemical spaces spanning 512 HIV reverse transcriptase inhibitors,^27^ 240 T4 lysozyme mutants,^177^ and 32768 ribonuclease H variants,^171^ as well as challenging perturbations of both ligands^186^ and proteins.^185^

### 5.2 Hybrid Sampling and Free Energy Algorithms

The calculation of solvation free energy and binding affinity of small molecules to macromolecules are among the most important practical applications of MD simulations, especially with the potential impact on drug discovery efforts. A wide range of methodological advances were implemented in CHARMM to improve the statistical convergence and physical accuracy of free energy calculations. Conceptual advances in free energy methodologies implemented in CHARMM were reviewed in ref (187). For example, a version of λ-dynamics was introduced via a MC multicanonical REMD (FEP/REMD).^188,189^ Specifically, the FEP/REMD helps resolve the poor convergence of the free energy estimates as a function of λ near the end points (λ = 0 and 1), which is often reflected as hysteresis between the forward (0 → 1) and backward (1 → 0) calculations from traditional FEP calculations based on single trajectories.

Applications to the calculation of the binding free energy of different kinase inhibitors demonstrated that the FEP/REMD algorithm was critical for tackling complex ligands accurately.^190−192^ A similar general strategy improved the convergence of multidimensional US calculations by swapping configurations from different windows via Hamiltonian REMD (US/H-REMD)^193^ (Section 7.1). Another issue concerns the sampling of solvent configurations. The binding of a ligand to a receptor frequently involves the displacement of a certain number of bound water molecules. This is not an issue if the binding site is in direct contact with the bulk solution. However, the convergence and accuracy in FEP/MD calculations can be severely compromised when a binding site is deeply buried and is inaccessible to bulk water. In this case, simple MD does not guarantee a complete sampling of the solvent during the FEP calculation. As an illustration, the binding of camphor to a deeply buried pocket in cytochrome P450cam causes about 7 water molecules to be expelled.^194^ To address this, standard MD was coupled with the grand canonical MC (GCMC) algorithm to allow the number of water to fluctuate in any chosen region during an alchemical FEP calculation.^194^ GCMC helps better sample the solvent configurations in the binding pocket that are poorly accessible to bulk solvent. It is also powerful by introducing fluctuations in the number of solvent molecules in FEP calculations carried out with a reduced model where only the region surrounding the binding site is explicitly considered while the effect of the surrounding solvent and protein is mimicked implicitly with the generalized solvent boundary potential (GSBP).^195^ Such a strategy made it possible to calculate the standard binding free energy of antibiotics to the peptidyl-transferase P-site of the bacterial ribosome.^196,197^

Over the years, increasing efforts were made to streamline free energy calculations, enabling automated calculation of the absolute solvation free energy of a large number of small drug-like molecules using explicit solvent.^198^ Moreover, collaborative efforts were made to test the accuracy and reproducibility of free energy calculations across different software packages.^199^ One of the principal advantage of CHARMM is that different methodologies can be naturally integrated within a single job. For example, a US formulation of equilibrium binding^200^ was used to characterize the binding specificity of a large number of SH2 domains^201^ with the generalized Born with a simple switching (GBSW) implicit solvent model.^131^ As another example, the PBEQ continuum electrostatics module of CHARMM^108,202^ conveniently allows one to directly access and read MD trajectory snapshots, and then combine its MM potential energy together with the solvation contribution based on the Poisson–Boltzmann and surface area approximation (PBSA). This MM/PBSA strategy, seamlessly integrated within CHARMM, has been used, for example, to process a large number of protein complexes to assess the binding specificity within a family of synaptic surface receptors.^203^

A growing family of hybrid sampling methods combining the strength of MD and Metropolis MC were tested and implemented, benefiting from the flexibility of the control flow from the native CHARMM scripting command language at the level of the input file.^204,205^ These algorithms typically consider new configurations generated by driving the system via a non-equilibrium MD (NEMD) trajectory that are subsequently treated as putative candidates for MC acceptance or rejection.^204,205^ The hybrid NEMD/MC algorithms can be exploited in a variety of context and offer a promising avenue to sample the configurations of complex systems. For example, the discrete ionization state of titratable residues can be sampled, effectively as a constant-pH simulation.^206^ Another example is to consider new configurations of an all-atom system generated by driving it via NEMD toward a configuration that originated from a CG simulation. It was shown that the CG-guided hybrid NEMD/MC algorithm can enhance the sampling of solvated peptides even with fairly rudimentary CG models as a guide.^207^

### 5.3 Optimal Variance Alchemical Path for Free Energy Calculation

Despite continuous development of free energy calculation methods,^28,99,208^ practical challenges impede their precision and possibly reliability.^45,199^ Options for improvement include enhanced sampling,^99,189,209−211^ careful design of alchemical cycles,^199,211,212^ variational and integration approaches,^211,213−216^ and the design of the alchemical path itself,^99,215,217−220^ the latter being the focus of this section.

The hybrid Hamiltonian method relies on the ‘optimal alchemical path’ theory.^217^ To overcome barriers between reactant and product phase spaces, it is implemented at the interaction pair level, treating each pair separately though in parallel. Denoting abolished (*A*) interacting pairs as *p*_*i*_ ∈ *P*_*A*_ and created (*B*) ones as *p*_*i*_ ∈ *P*_*B*_, the corresponding Hamiltonian contributions are
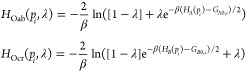
5where *H*_*A*_, *G*_*A*0,*i*_, *H*_*B*_, and *G*_*B*0,*i*_ are the energy functions and estimates for the free energy of abolishment or creation of each of the pairs, *p*_*i*_. Approximations for Eq. 5, denoted by *H*_cr_ and *H*_ab_, follow from Eq. 25 of ref (217), and the hybrid Hamiltonian is given by
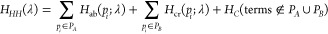
6where *H*_*C*_ is for all other terms unaffected by the transformation. The improper and proper dihedral angle fluctuations being modest, simple multiplication factors are used for created and abolished terms, respectively (isomorphous to Eq. 25 in ref (217)). For Ewald sum, a linear scheme for charge, *q* = *q*_*C*_ + (1 – λ)*q*_*A*_ + *λq*_*B*_, is used.

**Figure 2:**
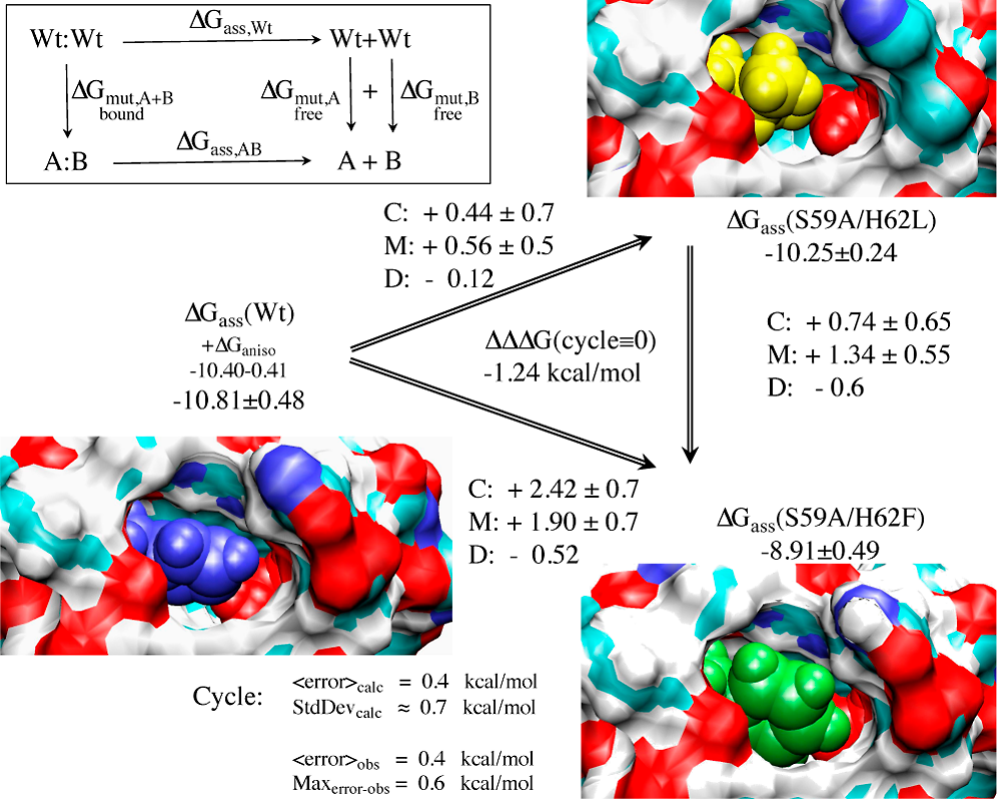
Thermodynamic cycle. Inset: general cycle design; horizontal arrows: measured affinities in kcal/mol;^222,223^ vertical arrows: computations for tetramer (left) and the two types of dimers (right). Graphical panels: local molecular surface at the interfaces with the mutated residues displayed as spheres for WT (S59: red, H62: blue), S59A/H62L (A59: red, L62: yellow), and S59A/H62F (A59: red F62: green). For WT, a 0.41-kcal/mol entropic term is added to account for higher symmetry.^222^ Computed (C:), measured (M:) differences, and discrepancies (D:) are given. Global discrepancy (ΔΔΔ*G*) for the 3 cycles provides a self-consistency check. Average standard deviation (StdDev_calc_) and error (⟨error⟩_calc_) were computed using autocorrelation functions^224^ considering λ windows as independent. The average error ⟨error⟩_obs_ and the maximum observed error Max_error-obs_ that compare experimental results with calculations are also reported.

As a result, the derivative with respect to λ can be intertwined as an additional dimension to that of the system spatial coordinates, **r**, extending Eqs. 4.6 and 4.9 of ref (176) as
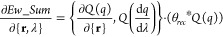
7where ∗ indicates convolution, *Q* the charge mesh, and θ_*rec*_ the reciprocal factor mesh defined in ref (176). An application of the method was on the R67 DHFR system that is a pseudo-homotetramer, a dimer of dimers.^221−223^ To simulate the mutation process, the two subunits of one dimer had an *A*-hybrid residue at position 59 and the two subunits of the other dimer had a *B*-hybrid residue at position 62 (Figure 2). Simulations were run sequentially for 10 discretized values of λ from 0 to 1. Hybrid residues were also subjected to TI in their isolated acetylated and aminated form as a control.

**Figure 3:**
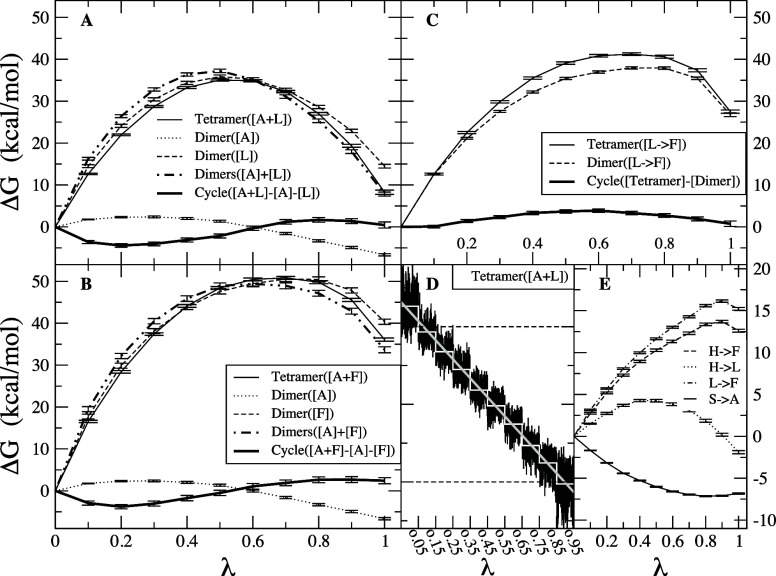
Integration along the thermodynamic cycles in Figure 2. Cumulative error estimates^224^ are also shown for the tetramer, each of the dimers, the sum of the dimers, and the global cycle for (A) WT to S59A/H62L, (B) WT to S59A/H62F, and (C) S59A/H62L to S59A/H62F for which only one dimer is involved since residue 59 remains as Ala. (D) Integrand for the transformation of the tetramer from WT to S59A/H62L shown in black as an example. The average for each λ window is marked by a stepwise white line. Linear regression along the whole trace is shown as a light gray line to appraise the linearity of the integrand with respect to λ. Dashed lines mark ±100 kcal/mol. (E) Integrand for isolated hybrid residues (acetylated and aminated) in a vacuum to evaluate the intrinsic energy contributions due to the FF energy difference of the original residues.

Branches of the various thermodynamic cycles in Figure 2 are further analyzed in Figure 3. Individual curves are bell-shaped, mirroring the quadratic form of the partition function of the optimal path as function of λ (Eq. 11 of ref (217)) and yielding a linear integrand. Due to differences in the position of the maxima for the different branches of a same cycle, the global cycle profiles are sinusoidal rather than quadratic, nonetheless very tempered.

As previously reported, S59A/H62L is favorable despite loss of a hydrogen bond and the formation of a small hydrophobic cavity.^222^ In comparison, S59A/H62F is less favorable despite good shape complementarity and creation of new hydrophobic contacts.^223^ Despite modest calculation effort only intended to illustrate the optimal alchemical path integrand properties, simulations reproduce those unexpected results. Interestingly, TI on isolated hybrid residues revealed the predominance of the amino acids intrinsic FF potential differences on the integrand, suggesting that reducing those differences could reduce the difficulty to reach accurate results. The linearity of the integrand with respect to λ for the method presented here facilitates integration, hence it is a desired property. It avoids the need for evolved integration schemes that can amplify errors, but are required to treat irregularity or singularity found, for example in conventional van der Waals creation.^214^

## 6 Constant pH Methods

### 6.1 Hybrid-Solvent and All-Atom Continuous Constant pH Methods

Describing protonation state changes due to a change in solution pH or conformational environment was first enabled in CHARMM through the GB CpHMD methods.^225,226^ In these methods, an auxiliary set of (λ) coordinates representing the evolution of protonation states are propagated based on the idea of λ-dynamics^173^ (Section 5.1). Since 2010, the CpHMD framework was further developed to be carried out in explicit-solvent MD simulations (see, e.g., Section 5.1). An example is the hybrid-solvent CpHMD^227^ that samples solute conformation in explicit solvent but leverages the GBSW implicit solvent model^131^ for propagating protonation states. The pH REMD method was also developed to accelerate sampling of the coupled conformation and protonation states.^227^ The hybrid-solvent CpHMD was later extended for transmembrane protein simulations^228^ by including the implicit membrane GBSW model^229^ with a water cylinder to account for water molecules in the pore of a channel or a transporter. To remove the dependence on the GB models which limits the accuracy, the all-atom CpHMD methods with generalized reaction field^230^ or PME for long-range electrostatics^179^ have been developed. To enforce net charge neutrality in all-atom CpHMD, an approach based on cotitrating ions^230^ or water^231^ has been developed. The hybrid-solvent and all-atom CpHMD have enabled not only new lines of inquiries, e.g., pH-dependent self-assembly mechanism of chitosan in which a total of 160 glucosamine units were allowed to titrate,^232^ but they also provided fresh perspectives to resolve old questions where, e.g., the hybrid-solvent CpHMD simulations revealed the formation of proton-coupled hydrogen bonds as a major determinant for acid/base.^233^

### 6.2 Constant pH MD with Discrete Protonation States

There are two main classes of constant pH simulations depending on whether the protonation states vary discretely (either deprotonated or protonated)^207,234−241^ or continuously.^172,225−227,242^ Two types of the former class are implemented in CHARMM. The first is based on the MD/MC constant pH method^237,238^ that is available only in implicit solvent. The second type is based on the EDS method,^37^ and is available for explicit solvent. Constant pH simulations with continuous protonation states, also in CHARMM, are available for implicit,^225^ combined implicit and explicit,^227^ as well as explicit-only solvent.^172^

The MD/MC method,^237^ originally available for Amber, has been implemented in CHARMM and further extended to include constant pH REMD.^238^ During MD simulation, attempts to change the protonation state according to the Metropolis criterion are made at a user-defined interval. The deprotonated state is modeled with the proton present following the charge distribution for the deprotonated state. For replica *i*, let the positions and momenta of atoms be *q*_*i*_ and *p*_*i*_, respectively, *N*_*i*_^*p*^ be the number of titratable residues that are protonated, and pH_*l*_ its pH. Similarly define *q*_*j*_, *p*_*j*_, *N*_*j*_^*p*^ and pH_*m*_ for replica *j*. Denoting *X*_*i*_^*l*^ ≡ (*q*_*i*_, *p*_*i*_, *N*_*i*_^*p*^, pH_*l*_) and *X*_*j*_^*m*^ ≡ (*q*_*j*_, *p*_*j*_, *N*_*j*_^*p*^, pH_*m*_), the probability of exchange between replica *i* and replica *j* is
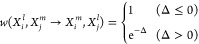
8
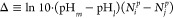


In addition to the constant pH REMD that greatly improves sampling of the protonation state,^238^ the reservoir constant pH REMD method was developed to better sample conformational states.^243^ It relies on pregenerated reservoirs of conformations with fixed protonation states. The reservoirs can be generated either by long MD simulations, or with an enhanced sampling method, so that conformations with a given protonation state follow the Boltzmann distribution. Then an attempt to replace the current conformation with a random reservoir structure is made after a given number of steps. The attempt is accepted if protonation states of all ionizable residues match with those of the reservoir structure, and rejected if not. In this way, the system can sample conformations from the Boltzmann ensemble of the reservoir.

Another method implemented in CHARMM is the EDS with Hamiltonian REMD (EDS-HREM).^40^ In the EDS approach,^37^ a hybrid Hamiltonian enveloping both states is defined such that the corresponding partition function is the sum of partition functions for individual Hamiltonians. In addition, a smoothness parameter can be introduced to facilitate conformational transitions between states with high energy barrier. In its constant pH implementation,^40^ the two states are protonated and deprotonated, and a pH-dependent energy offset between the two states is introduced.
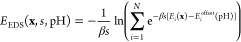
9Here, *E*_*i*_(**x**) is the potential energy of state *i* with coordinate **x**, *s* is the smoothness parameter, and *E*^offset^(pH) is the pH-dependent energy offset calculated ahead of the simulation via thermodynamic cycling. The Hamiltonian can be extended to several titrating groups, conveniently describing clusters of coupled residues. For example, it has been used to calculate the p*K*_a_ values of four glutamic acid residues in the selectivity filter of a sodium channel.^244^

Different replicas have different values of *s*, which allows for replicas with low *s* (very smoothed) to cross energy barriers, while replica with *s* = 1 yields the conformational ensemble identical to the semigrand canonical ensemble at convergence. As a follow-up, a 2-dimensional (2D) replica exchange pH method was added in CHARMM, where the second dimension is pH.^41^

FEP methods have also been used for protein p*K*_a_ calculation in both implicit and explicit solvent,^245^ as well as in QM/MM settings.^246^ In a recent study, p*K*_a_ calculations from the 2D EDS-HREM in explicit solvent have been found to agree well with FEP results for a complicated system consisting of four selectivity filter glutamate residues of an ion channel with bound ions.^247^ Additional FEP simulations led to a new proposed mechanism of selectivity in this ion channel, based on the shift of the p*K*_a_ value in the presence of different ions.^247^

### 6.3 Proton Hopping Simulations

Classical biomolecular MD simulations normally do not allow changes in covalent bonding. This is an issue in systems involving proton transfer, as e.g. in proton diffusion in water where a proton breaks a bond with one water and forms a new one with a neighboring water molecule. The MOBHY (for “mobile hydrogen”) module in CHARMM allows proton mobility by interspersing discrete proton moves during a dynamics trajectory.^248^ After a given number of MD steps, an attempt is made to move a titratable proton to an eligible alternative location, i.e., a potential acceptor to which the titratable proton is hydrogen bonded. Upon the hop attempt, the molecular geometries and FF parameters of protonated and deprotonated species are changed accordingly. The missing protons are represented by dummy atoms (no charge and no interactions with surroundings). The initial protein structure is generated with all potential protons present, i.e., all specified titratable residues should be fully protonated in the PSF (whether they are truly protonated is selected by the user). Thus, no actual changes in bonding take place during a proton hopping simulation; only the atom types and charges change. The excess proton is represented as a classical hydronium ion. Acceptance of a proton move is based on a Metropolis-like criterion that employs an empirical threshold for the energy change upon proton hopping. The threshold is chosen to reproduce the experimental proton diffusion coefficient in water. Similar empirical thresholds are used for proton hopping between water and protein side chains, while the true rates can be obtained by more elaborate methods.^249^ This method has been applied to proton conduction by gramicidin A,^248^ investigation of the asymmetry of proton conduction in the influenza M2 proton channel,^250^ and evaluation of models for the human voltage gated proton channel.^251^

## 7 Enhanced Sampling and Transition Path Methods

### 7.1 Replica Exchange MD (REMD)

In REMD, *N* independent copies (or replicas) of a system are run in parallel and are periodically swapped (i.e., exchanged) to enhance the crossing of potential energy barriers.^252,253^ REMD is useful in systems where energy barriers lead to poor sampling and slow convergence in conventional MD, hindering accurate calculation of thermodynamic quantities.^188,189,254^

When the system volume does not change, the probability of observing a system in a configuration represented by coordinates *X* and Hamiltonian *a* with energy *E*_*a*_ ≡ *E*(*X*) is
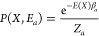
10where β_*a*_ = 1/*k*_*B*_*T*_*a*_, with Boltzmann constant *k*_*B*_ and temperature *T*_*a*_. *Z*_*a*_ is the partition function. For *N* non-interacting replicas, the probability of observing the system in a particular state is the product of the probabilities for individual replicas:
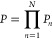
11The enhancement of sampling in REMD comes from periodic swapping of the coordinates and velocities between two replicas. By imposing detailed balance, the ratio of the forward and backward transition rates between replicas *a* and *b* in exchanging their coordinates *X* and *Y* is given by^253^
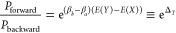
12where *e*^Δ_*T*_^ is the temperature replica exchange probability. In Hamiltonian REMD, *e*^Δ_*H*_^ can be similarly defined. They are used to accept or reject the exchange using the Metropolis criterion:
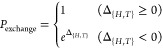
13

When the system’s volume changes (NPT ensemble), Eq. 10 changes to^255^
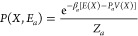
14where *P*_*a*_ is the external pressure at Hamiltonian *a* and *V*(*X*) is the volume of the coordinates *X*. The exponents of *P*_exchange_ then become
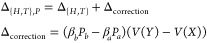
15

REMD in CHARMM is handled through the REPD (REPlica Distributed) command. It requires MPI parallelism with one or more MPI processes per replica. Exchanges are attempted at a user-specified interval, typically on the order of 1 ps. The exchange direction alternates between “up” and “down” in the replica space. While it is not strictly necessary to attempt exchanges only between neighboring replicas, acceptance of an exchange between two replicas requires overlap between their potential energy distributions that is typically highest for neighboring replicas. Exchanges are accomplished by swapping coordinates and velocities between MPI processes, so that each MPI process yields a “replica” trajectory, i.e., coordinate frames corresponding to a single temperature or Hamiltonian.

CHARMM supports REMD for temperature, general Hamiltonian, self-guided Langevin,^256^ and CpHMD using either discrete^237,238^ or continuous^179,225,227^ protonation states. CHARMM also supports coupling of the top and/or bottom replicas (i.e., the highest and lowest in replica space) to pregenerated structure reservoirs. Exchanges with the reservoir can further accelerate conformational sampling,^257^ and can be done assuming either Boltzmann (recommended)^258^ or non-Boltzmann^259^ weighting. In constant pH REMD, CHARMM supports exchanges with reservoirs that have fixed protonation states, where exchanges with a structure in the reservoir can only be accepted if the protonation state of all ionizable residues matches the structure to be exchanged (Section 6.2).^243^

CHARMM also supports multidimensional REMD,^260,261^ with the only restriction being that a dimension aside from the general Hamiltonian may only be used once (e.g., one temperature dimension and one self-guided Langevin dimension is permitted, but not two temperature dimensions). The combination is multiplicative: For example, a setup with 4 temperatures and 2 Hamiltonians will use 8 replicas in total. To simplify scripting, CHARMM sets up several user-accessible variables, such as ?NREP and ?MYREP, which refer to the total number of replicas and the global replica index respectively. For multidimensional REMD, ?NREPD⟨*X*⟩ and ?MYREPD⟨*X*⟩ refer to the total number of replicas and replica index in dimension ⟨*X*⟩, respectively (for example, ?NREPD1 is the number of replicas in the first replica dimension).

REMD in CHARMM can be combined with other ensemble methods such as EDS^37^ via the MSCALE module.^262^ Earlier, a constant pH method in explicit solvent with discrete protonation states was developed based on a combination of EDS and a 1D REMD.^40^ A more recent version features EDS with a 2D REMD (the second dimension being pH), which significantly accelerates the convergence of constant pH simulations.^41^

### 7.2 Biasing Methods

#### 7.2.1 Targeted MD (TMD)

Conformational transition pathways can be simulated with a number of TMD methods. The original implementation^263^ introduces a holonomic constraint that reduces the RMSD from the target coordinates with a preset value at each MD step. While this guarantees to reach the target conformation, generated pathways are generally irreversible^264^ and they can cross large free energy barriers.^265^ By using a perturbation of a fixed magnitude that minimizes the RMSD with the target at every step, the restricted perturbation TMD (RPTMD) method^265^ generates low free energy pathways along which potential of mean force (PMF) profiles can be readily calculated.^266^ The RMSD can also be decreased by a restraint potential (RTMD) that can be symmetrized to yield more reversible paths.^264^ Due to the use of global best-fit rotations, these TMD methods tend to favor large scale motion before small conformational changes,^265,267^ which is subdued in locally restrained TMD (LRTMD) by applying a number of TMD restraints on subsets of atoms.^267^

#### 7.2.2 Related Conformational Free Energy Sampling

CHARMM supports a number of enhanced sampling techniques to evaluate conformational free energy differences. US^138^ and adaptive US^268,269^ of distances, angles, torsions, RMSD, and more complex geometrical order parameters are supported by the CONS, RXNCOR, and ADUMB modules. US is typically performed through the use of harmonic restraints that bias the system toward a desired target. CHARMM also supports best-fit positional restraints in which the reference coordinates are first rotated and translated to minimize the restraint energy. These best-fit restraints are key to the efficiency of confinement methods^270−274^ that calculate conformational free energy differences by transforming (part of) the system to the desolvated harmonic oscillator state. The Gaussian-mixture US (GAMUS) method allows enhanced sampling of multidimensional order parameters (3–6 dimensions).^275,276^ Like adaptive US, GAMUS uses the negative of the calculated free energy as the biasing potential, which is updated periodically while taking all sampled data into account. GAMUS constructs its biasing potential from a Gaussian-mixture model that fits the probability distribution using fully optimized Gaussian functions. By foregoing grids, GAMUS can sample higher dimensional spaces than traditional adaptive US. CHARMM also supports Tsallis-based biasing potentials^277^ that increase sampling by reducing the force near energy barriers. In CHARMM, Tsallis-based sampling can also be coupled to replica exchange with solute tempering^278,279^ for faster sampling.^280,281^

### 7.3 String Method (SM) for Conformational Transitions

If a process of a system with positions **x** is described by the reaction coordinate , the free energy  of a conformational state *q*(**x**) = *q*_0_ is
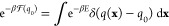
16One often wishes to follow the progress of an actual chemical or physical reaction as *q*_0_ is varied from the initial (reactants) to the final (products) value. Below, we focus on a set of methods in which the reaction coordinate is optimized from an initial pathway or a set of intermediate configurations.^282−287^

**Figure 4:**
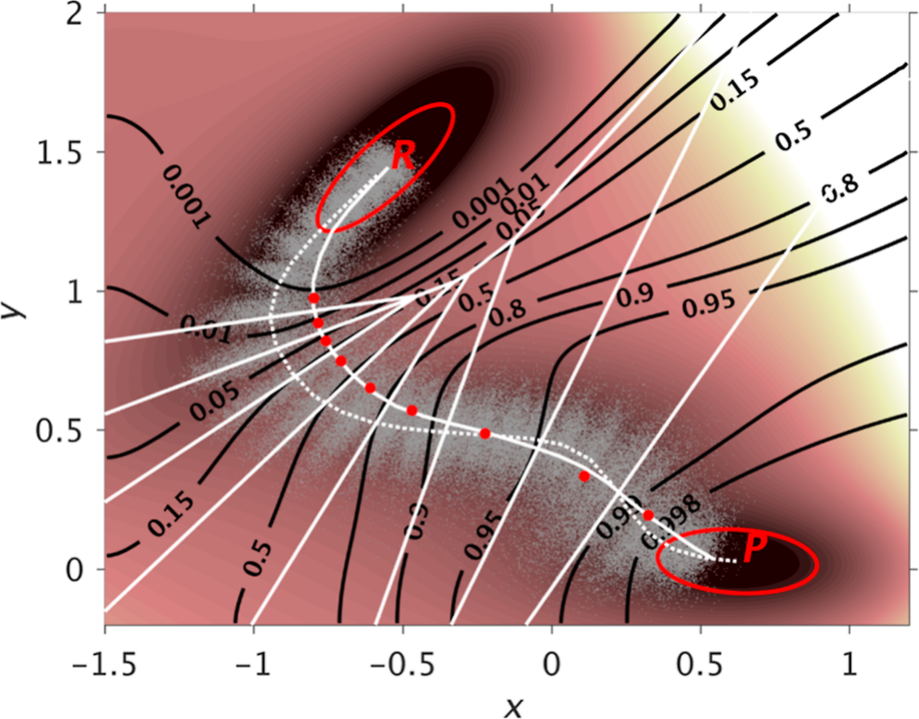
Illustration of the SM on the 2D Mueller potential. An MEP (dotted white curve) and a finite temperature string (solid white curve) connect the reactant (R; *q* = 0) and product states (P; *q* = 1) enclosed within red ellipses. Black contours represent isocommittor surfaces obtained from a 2nd order finite difference solution of the backward Kolmogorov equation for overdamped Langevin dynamics. White straight lines are planar approximations to the isocommittor surfaces, which also partition the configurational space into a Voronoi tessellation with nodes (red bullets). Gray dots are simulation coordinates from overdamped Langevin dynamics restrained to reaction coordinate planes and collectively define a transition tube.

The essential idea of SM^288−290^ is to assume that the optimized path is everywhere tangent (possibly up to a constant multiplicative tensor) to the reaction coordinate gradient without needing to specify an analytical form for it (Figure 4). Three versions of SMs implemented in CHARMM are described below.

#### 7.3.1 Zero-Temperature SM

The zero-temperature SM (ZTSM) computes a minimal-energy path (MEP) which is a curve in the space of *N*_*a*_ atom coordinates defined as  that, for any *α* ∈ (0, 1), satisfies
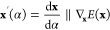
17where **x**(0) and **x**(1) correspond to the reactant and product state, respectively. The ZTSM evolves an initially assigned guess to the MEP using the steepest descent (SD) minimization while enforcing uniform parametrization by arc length, |d**x**/d*α*| = constant. In the CHARMM implementation, the continuous string is discretized into *N* replicas or ‘images,’ each assigned to a separate group of processors for parallel execution
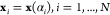
18with *N* typically determined by the available computing processors. The string evolves to the MEP as
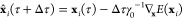
19
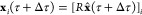
20where Δ*τγ*_0_^–1^ controls the speed of SD evolution (Δτ is an artificial time step, and γ_0_ is a friction constant that ensures dimensional consistency).

Eq. 19 is advanced independently for each image, and *R* is the reparameterization operator that corrects the provisional coordinates **x̂** so that |**x**(α)′| is constant along the string. *R* is common to the SMs in CHARMM (Section 7.3.4). The evolution step (Δ*τγ*_0_^–1^) and convergence criteria can be set manually, or automatically by the SD minimizer of CHARMM. While SD is the default minimizer for ZTSM, other minimizers in CHARMM can also be used.

#### 7.3.2 Finite-Temperature SM

The finite-temperature SM (FTSM) can be derived from the backward Kolmogorov equation (BKE)^291^ corresponding to overdamped Langevin dynamics.^292−295^ In FTSM, the desired reaction coordinate *q* is assumed to be the *committor* function that solves the BKE,^291^ and the committor isosurface *q* = *q*_0_ is approximated by a hyperplane (see Figure 4)
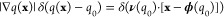
21The Jacobian |∇*q*(**x**)| preserves the volume and **ν**(*q*_0_) is the unit normal to the hyperplane *P*_*q*_0__ that approximates the isosurface *q*(*x*) = *q*_0_. **ϕ** is constrained by
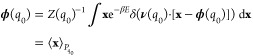
22where *Z*(*q*_0_) = ∫e^–*βE*^*δ*(***ν***(*q*_0_)·[**x** – ***ϕ***(*q*_0_)]) d**x** is the partition function of the hyperplane. In analogy with an MEP, we can parametrize a continuous curve **ϕ**(*q*_0_(α)) having d*q*_0_/d*α* > 0, and identify it with the average reaction path. Provided that the transition ‘tube’ (Figure 4), as measured by the variance of |**x** – **ϕ**(*q*_0_)|, is not too large, **ϕ** also represents the dominant reaction path. From Eqs. 21 and 22, it can be shown that^292^
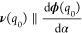
23i.e., the reaction coordinate hyperplanes are locally perpendicular to the reaction path (string). In FTSM, Eqs. 22 and 23 are iteratively solved.^285,293,294^ From an approximation to the string at iteration *n* (**ϕ**^*n*^), one obtains **ν**^*n*^ using Eq. 23, which permits computing **ϕ**^*n*+1^ using Eq. 22. This is repeated until **ϕ**^*n*^ does not change (up to thermal noise). The free energy can then be obtained by TI of the free energy derivatives sampled on the hyperplanes,^285,294^ or by sampling a Voronoi tessellation (Figure 4).^290^

The FTSM in CHARMM can optionally use Hamiltonian REMD to accelerate sampling, and an upper bound on the transition tube width can be set to limit sampling near a predefined path. In a parallel implementation,^296^ FTSM starts from an initial string discretized into *N* images ***ϕ***_*i*_^0^, *i* ∈ {1, ..., *N*}, which can be obtained from, e.g., an MEP or a biased dynamics trajectory. To each image **ϕ**_*i*_ one assigns a separate CPU group and a complete all-atom MD simulation system denoted by **x**_*i*_, to be used for sampling each reaction coordinate hypersurface. Each CPU group receives the neighbor images **ϕ**_*i*±1_ in addition to ϕ_*i*_, which are required to compute **ν**(*q*_0_) in Eq. 23, and samples the hyperplanes independently of the other groups.

#### 7.3.3 String in Collective Variables

There are cases when variables other than Cartesian coordinates, e.g., distances,^297^ are more suitable for the reaction coordinate. Following the steps in ref (289), SM in CHARMM has been reformulated in a coarse-grained (CG) space of collective variables (CVs).^139^ Assume that the reaction coordinate is determined by a set of CVs θ_*j*_(**x**) (*j* = 1, ..., *K*) *via* some function *f* (which does not need to be specified explicitly): *q*(**x**) = *f*(*θ*_1_(**x**), *θ*_2_(**x**), ..., *θ*_*K*_(**x**)). The coarse-graining leads to a *K*-dimensional free energy landscape as a function of CV coordinates denoted by **z**:
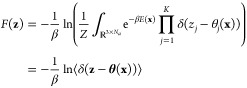
24and a metric tensor **M**(**z**) defined by
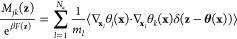
25where *m*_*l*_ is the mass of atom *l*. Using *F* and **M**, it is possible to write down Langevin equations governing the evolution of **z**.^289^ Further, assume that the reaction proceeds *via* a localized reaction channel that contains a minimum *free* energy pathway (MFEP) on the CV landscape
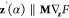
26with parameter α ∈ [0, 1] and |**z**′(α)|′ = 0 (equal arc length) in analogy with Eq. 17 for the MEP.

The SM in collective variables is an iterative algorithm for computing the MFEP using local averaging of the force ∇_**z**_*F* and metric tensor **M** obtained from restrained MD simulations.^139,289^ After the string converges to the MFEP, two types of free energy profiles can be computed, *F*[**z**(α)] in the *K*-dimensional space of the CVs, and a 1-dimensional profile  associated with the reaction coordinate hyperplanes on **x**. An approximate calculation of  in CHARMM is implemented using Voronoi tessellation, which also allows computation of the mean first passage time along the reaction coordinate using the Markov state model.^290^

#### 7.3.4 Reparameterization

The SMs described here involve optimization of continuous curves (strings) specified by a parameter, e.g., {***ϕ***(*α*), *α* ∈ [0, 1]}. In numerical implementation, a set of discrete points along a string are used instead. To maintain uniform string resolution, parametrization by arc length is used, i.e.,
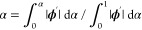
27which implies that |**ϕ**′| is constant along the string, or that |**ϕ**_*i*_ – **ϕ**_*i*–1_| is constant for all images *i* > 0. Because the string deforms as it evolves, points (images) along the curve ϕ(α) must be periodically reassigned to satisfy equidistance. This *reparameterization* operation (*R* in Eq. 20), is implemented by interpolating the string onto a refined parameter grid, i.e., α_*j*_, *j* = 1, ..., *N*_*f*_, with *N*_*f*_ = 5 × *N*, computing arc length on this grid normalized to the unit interval, and interpolating onto the original uniform parameter grid. Linear interpolation is the default and recommended method. Others such as B-splines and cubic splines can also be used.

#### 7.3.5 String with Swarms-of-Trajectories

Rather than refining the string in the multidimensional space of CVs by estimating the average force and metric tensor from restrained trajectories via Eqs. 24 and 25 as described above, an alternative approach considers the average dynamic drift of those variables determined on-the-fly via ensemble of short unbiased trajectories starting at different points along the string.^298^ One advantage of this so-called “SM with swarms-of-trajectories” over the traditional procedure is that the computational task can be naturally distributed over many computer nodes with negligible interprocessor communication. The formal equivalence between the two approaches in the limit of very short trajectories was established,^299,300^ and their respective significance has been clarified.^300^

#### 7.3.6 Script-Based SM Approach and Structure Building

In a first application of the SM with swarms-of-trajectories to an all-atom solvated protein,^298^ the activation pathway of Hck kinase and the inactivating DFG-flip were determined.^301,302^ It bears emphasizing that the SM could be scripted directly in the input file of CHARMM, and required no new source code. The powerful scripting facilities within CHARMM, especially the ability to modify the bonding topology of the system on the fly using the Patch Residue (PRES) facility, made it possible to generate all-atom models of the polymerized FT-30 membranes, which are widely used in reverse osmosis operations.^303,304^

### 7.4 Adaptively Biased Path Optimization (ABPO) for Transition Path Sampling

Algorithms to compute the energetics and conformations associated with protein conformational transitions are most often based on path-restrained sampling using a chain-of-states defined at specified intervals along the path. The ABPO method^305^ is an alternative approach that does not require the protein system be restrained to the path. ABPO is implemented in CHARMM through the ENSEMBLE module with options for defining CVs (also called reduced variables, RVs) and path optimization parameters. An adaptive biasing potential, *V*_*b*_, is utilized to enhance sampling of the path without restraining the system to specific points on the path^306^
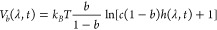
28where *b* is the fraction of the free energy flattened by the bias, *c* has an inverse time unit and controls how the bias couples to the dynamics. *V*_*b*_ adapts from the sampling histograms *h*(λ, *t*) that counts visits to the region of the path around λ over time *t*. The PMF is a direct result of the adaptive bias potential obtained for the optimal path.

**Figure 5:**
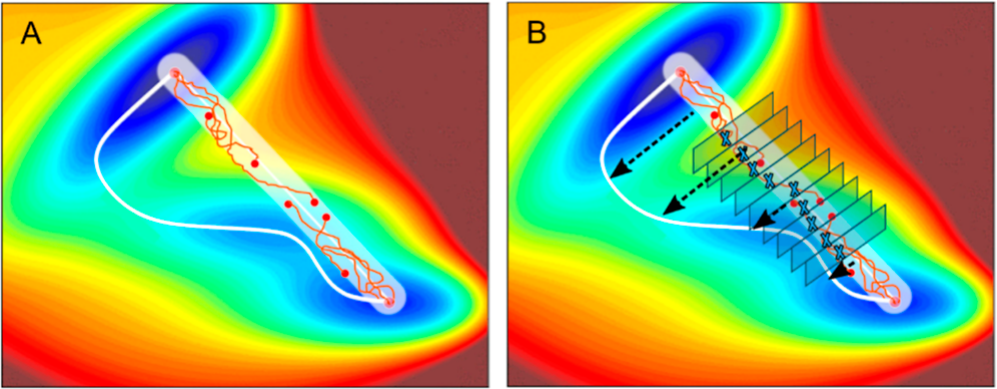
Illustration of ABPO. (A) Energy landscape in the CV space at the initial stage of path optimization. Multiple trajectories are launched from the two end-state energy wells (blue) and sample freely along an arbitrary initial path (red line) enhanced by *V*_*b*_ and within a tube centered on the path (white transparent rectangle) by a tube potential. (B) Trajectory visits to hyperplanes (small gray rectangles) perpendicular to the path tangent are counted. After sufficient sampling, the mean position in each hyperplane from counts over all replicate trajectories (blue X’s) is determined, and the path and tube center are updated to these new values in CV space. The process is repeated to move the path incrementally (dashed arrows) until convergence to the optimal one (white curve).

A second distinction of ABPO compared to path-restrained methods is that evolution of the ABPO path begins by initiating multiple trajectories from an equilibrium ensemble simulated at each end state (Figure 5A). As such, the generation of unphysical structures at specified intervals along the initial chain-of-states path is avoided when starting the ABPO calculation. As trajectories move out of the end-state basin, their proximity to the path is retained with a tube potential of specified radius and centered on the path. An advantage of free sampling within the tube is to reduce frustration in sampling a rugged free energy landscape.

Formulation of the ABPO path follows that of the finite-temperature SM^307^ (*cf.,*Section 7.3.2). The path is specified by CVs, the definition of which is key for the computation of the PMF. Sampling of the path is counted in terms of hits to hyperplanes orthogonal to the tangent at each path index point, and statistics over multiple trajectories in a time period are used to update the ABP. The path is evolved by computing the mean position of trajectory hits in the hyperplanes and updating the path variables to coincide with those of the mean (Figure 5B). A redistribution of the updated path index points is needed for smoothing and respacing using a mollifier.^305^ The optimum path is reached when the distance between the last and penultimate curves falls below a specified threshold.

The PMF *A*(λ, *t*) along the path parametrized with λ (within an additive constant) is computed from the histograms obtained from exhaustive sampling of the optimized path over time *t*,
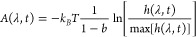
29As a directed approach, ABPO readily affords an atomistic description of a transition process in a reasonable simulation time depending on the choice of the selected CVs. Further, ABPO samples in a tube region surrounding the path and thereby generates a range of conformations orthogonal to the path that would not be obtained with path-restricted methods. The algorithm also provides a convenient way to assess the choice of CVs as well as the convergence of the path by following the time-course of individual CVs as a function of λ, so-called CV plots.^308,309^ ABPO has the potential limitation of insufficient sampling in regions of high free energy, whereas path-restrained methods by nature ensure sampling all parts of the defined path.

### 7.5 Reaction Path Optimization with Holonomic Constraints

When studying protein conformational changes, a chain of intermediate replicas of the system resolve the transition between the initial and final states. To find the most probable pathway, an objective function such as the total energy or free energy of replicas is defined and minimized.^282,307^ Success of reaction path optimization depends on auxiliary schemes to ensure proper distribution of replicas for capturing kinetic bottlenecks.^310^ In general, it is desirable to maintain equal distances between neighboring replicas while the distance is free to change since the actual reaction path is not known *a priori*. A folded-back path should also be avoided as replicas are placed to take forward steps in crossing kinetic barriers rather than going back and forth in a basin. In this regard, the angles between three consecutive replicas are often restrained^310^ to prevent drastic changes in the tangent vectors along the path that are represented by the position vector differences of replicas. A key challenge of reaction path optimization is the auxiliary scheme of managing path quality interfering with the optimization of the objective function. Keeping equal distance between replicas, for example, tends to conflict with the forces along the path in energy minimization. Although the tangential component of the force can be removed,^311^ the non-conservative projected force makes the application of fast-converging gradient-based optimization methods difficult.^312^ The robustness and efficiency in capturing low-energy kinetic barriers are thus limited, especially with a large number of degrees of freedom and a rugged potential energy surface (PES).

The RCONS module in CHARMM overcomes this by treating equal distance between replicas as holonomic constraints.^313^ Built on top of the REPLICA module, the reaction path optimization with RCONS is entirely gradient-based, readily allowing quasi-Newtonian methods and other optimization schemes assuming conservative forces. With Lagrange multipliers in constraint optimization, *ad hoc* numerical procedures such as rearranging atomic positions or force projections are not needed.^313^ Furthermore, the distance between replicas can be defined by using a non-commutative RMS best-fit procedure^312^ that is particularly useful for modeling transitions of macromolecules. Convergence of reaction path optimization provides a way to analyze if a sufficient number of replicas are used by testing whether the accumulated work along the optimized path agrees with the potential energy difference.^313^ Since the tangent vectors in this work-energy analysis are based on positional differences between replicas, the energy or free energy difference along a path can be decomposed into contributions from different atoms to deduce the kinetic bottleneck.^314^ It was also found that the straightness over replicas can be formulated as a kinetic energy potential and a temperature scale can be used to characterize the restraints regulating curvatures along the path.^313^

In principle, any potential energy function can be used to describe the energetics of replicas, and using RCONS with MSCALE provides a versatile framework for the general applications of reaction path optimization. Each replica along the path is treated as a subsystem for using a CHARMM potential energy function or in programs supported by MSCALE such as those providing a QM or QM/MM PES. For complex reactions involving conformational changes, implicit solvent model can be used to obtain an initial MEP followed by explicit-solvent MD simulations to obtain MFEP.^315,316^ RCONS can also be used to constrain the sampling of MD simulation over perpendicular directions to compute the PMF along a path. In this case, the chain defined by replicas is used as a 1-dimensional order parameter for the PMF calculation.^315,316^ Coupled with trajectory analysis, MD simulations constrained on the hyperplanes along a reaction path provide information about mechanistic details of a transition pathway. For example, the VIBRAN facility in CHARMM^317^ can be used to compute the scale-free mechanical coupling network in proteins and nucleic acids.^318−321^

### 7.6 Boxed MD (BXD)

**Figure 6:**
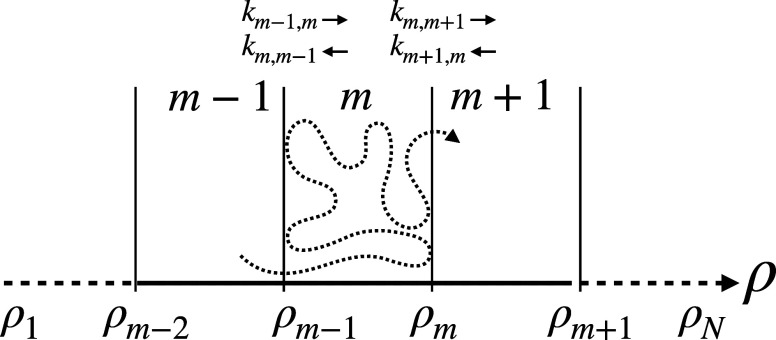
In BXD, the range of values assumed by the CV ρ is partitioned in boxes separated by reflective boundaries.

BXD^322,323^ is a simple technique to estimate rates and PMF *G*(ρ) along a CV ρ in a single MD simulation. BXD falls within a class of sampling methods such as milestoning^290,324^ where molecular configuration space is divided into a set of boundaries (or hypersurfaces). ρ is kept within a perfectly reflecting “box” for a time interval sufficiently long to reach convergence. This is done by reversing the velocity of the particles involved in the definition of ρ. After a given number of collisions with the boundaries, ρ is allowed to increase or decrease so that a neighboring box can be sampled. From the number of collisions with the boundaries, *G*(ρ) over the whole range of ρ can be reconstructed, as well as the absolute rate of entering or exiting a specific “box” (Figure 6). Velocity inversion is carried out at each of the boundaries. Assuming that at a certain time the trajectory is in box *m*, i.e., ρ_*m*–1_ < ρ(**r**) < ρ_*m*_, the transition rate from box *m* to box *m* + 1 is: *k*_*m*,*m*+1_ = *h*_*m*,*m*+1_/*t*_*m*_, where *t*_*m*_ is the time the trajectory spends in box *m*, and *h*_*m*,*m*+1_ is the number of hits (i.e., velocity inversions) at the boundary between ρ_*m*_ and ρ_*m*+1_. After the forward and reverse transition rates are determined, the equilibrium constants between the neighboring boxes *m* and *m* + 1 is
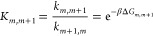
30

The free energy *G*_*m*_ can be determined by setting e.g., *G*_1_ = 0, and the probability of finding ρ in box *m* is
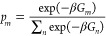
31which can be multiplied by the normalized probability *P*_*m*_(ρ) estimated from the histograms within boxes to obtain the probability distribution function *P*(ρ) = *p*_*m*_*P*_*m*_(ρ), or equivalently, *G*(ρ) = −*k*_*B*_*T* ln *P*(ρ). In practice, the user sets the position of the boundaries and the number of times the trajectory hits a boundary before it is let into the adjacent one. Both affect the convergence, which can be assessed by performing a single simulation spanning multiple times in both directions over the range of ρ(**r**) (from the lowest value to the largest, and *vice versa*). BXD is generalizable to multidimensional CVs using a general velocity-reflection procedure that conserves energy.^325^

### 7.7 Extended Adaptive Biasing Force (eABF) Method

Adaptive Biasing Force (ABF) is based on estimating the average force acting along a chosen CV, ξ, in order to construct and apply a biasing potential *f*_*m*_(ξ) that augments fluctuations of targeted dynamics.^326−328^ The classical ABF method is based on TI^329^ of the average force estimates which are computed in bins along the CV,
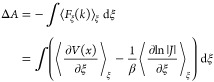
32where Δ*A* is the free energy difference,  is the average force along ξ in the *k*-th bin, *V*(*x*) is the potential energy function, and |*J*| is the determinant of the Jacobian.^327,330^

The biasing force  effectively flattens curvatures in the potential energy surface encountered along ξ, allowing for extensive sampling of transitions along ξ. The biasing potential is adaptive because it is updated by the current estimate of the average force along ξ until convergence.^331^

Estimating  brings about complications that hinder the utility of ABF.^330^ For example, calculating *∂*ln|*J*|/*∂ξ* in Eq. 32 can be challenging.^332^ The extended ABF method (eABF) was developed to overcome these limitations by introducing an extended potential energy function
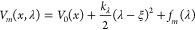
33where *V*_*m*_(*x*, λ) is the extended potential energy, λ is a virtual particle, and *k*_λ_ is the associated spring constant.^333−336^ The key distinction of eABF from ABF (Eq. 33) is the extension of the system via the λ particle; force estimates are now calculated via Hooke’s law and the biasing potential is applied to λ, which augments transitions in ξ via the harmonic coupling. Since the force estimates come from the harmonic restraint between λ and ξ, the recovered PMF (along λ) may deviate from that of the physical system (along ξ) depending on the coupling strength. Several estimators have been developed to recover PMF.^331,336^

**Figure 7:**
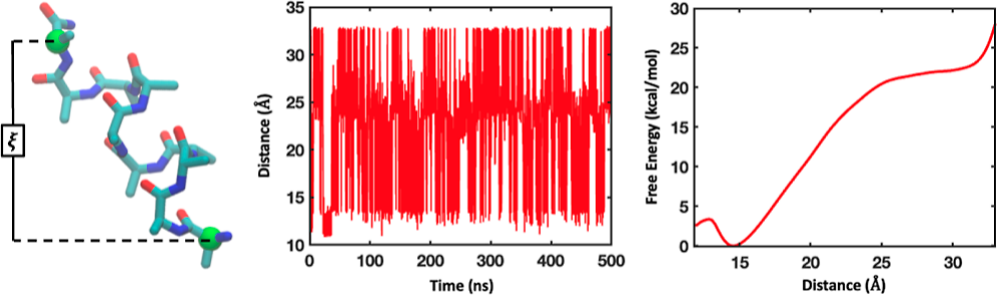
End-to-end distance (ξ) over time and the PMF of deca-alanine obtained using eABF.

As an illustration, a simulation of gas-phase deca-alanine was performed where ξ was defined as the end-to-end distance between the terminal C_α_ atoms. In 500 ns, a number of transitions between the helical state and extended states are realized, with an accompanying PMF along the distance consistent with previous studies (Figure 7).^331,337^

## 8 Advanced Energy Functions, Coarse Graining, and Implicit Models

### 8.1 Multipolar Electrostatics

Anisotropic charge distributions can be conveniently represented as a superposition of atom-centered multipoles.^338^ Halogen modifications are a noteworthy example which lead to a σ-hole on the halogen atom. Such features can be represented by using multipole expansions, often up to quadrupoles.^339−343^ Multipole-based electrostatics requires introducing local axes to define the orientation of higher-order multipole moments relative to the molecular geometry.

Multipolar interactions have been considered early on in molecular recognition.^344^ Compared to the spherically symmetric field around a single point charge, atomic multipoles can better capture anisotropic interactions. An example is carbon monoxide that cannot be modeled well with only atom-centered point charges located at nuclear positions of the two atoms because the total charge (*Q* = 0) and the total molecular dipole μ = 0.048 *ea*_0_ (*e* = 1.6 × 10^–19^ C, the charge of an electron, and 1*a*_0_ = 0.53 Å, the Bohr atomic length) lead to two opposite partial charges that are small in magnitude. In order to describe its substantial quadrupole moment^345−347^ either a third interaction site halfway between the two atoms is included^348^ or the two atoms are described by a distributed multipole expansion.^338,349−351^

The electrostatic potential (ESP) around a molecule can be represented in general as an expansion in multipole moments where the zeroth order contribution arises from atom-centered point charges. Capturing strongly anisotropic and/or directional features, e.g., lone pairs, hydrogen bonding, π-electron density or σ-holes^352−354^ requires a description beyond a single partial charge at each nuclear position. The ESP Φ(**r**) is related to the electron charge density ρ(**r**) through^355^
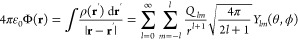
34where **r** and **r**′ are spatial variables and 1/|**r** – **r**′| was expanded in powers of *r*′/*r* < 1 to represent the ESP as a sum over spherical harmonics *Y*_*lm*_(θ, ϕ) from which the spherical multipole moment *Q*_*lm*_ is defined as
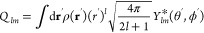
35The above can be integrated to yield a compact atom-centered representation of the ESP around a molecule and are used together with the MTPL module of CHARMM.

**Figure 8:**
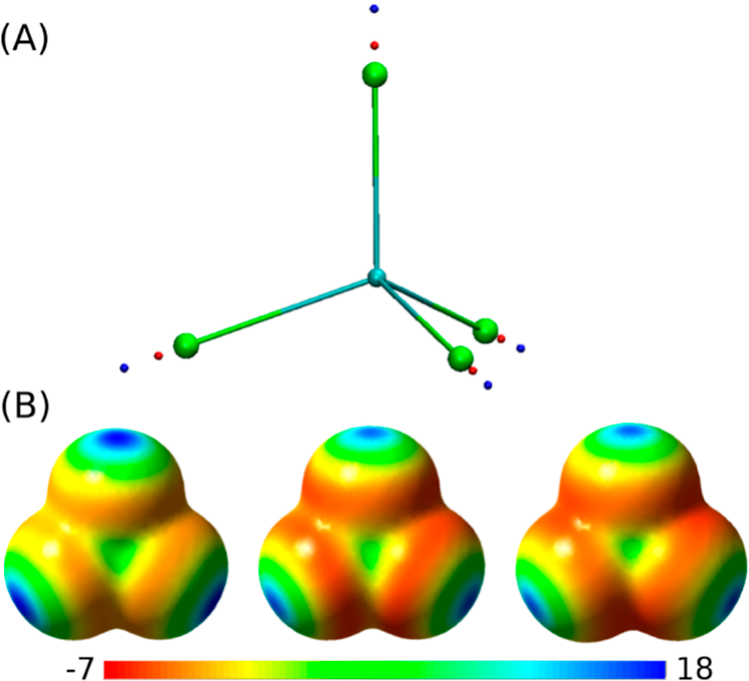
(A) 9-charge symmetry-constrained MDCM for CCl_4_. Red and blue points respectively correspond to negative and positive charge positions. A charge at the C-atom nuclear position is hidden. (B) DFT reference ESP (kcal/[mol·e]) mapped onto the 0.001 au molecular isodensity surface (left); fitted multipolar model truncated at quadrupole (middle); 9-charge MDCM model (right, root-mean-square error (RMSE) of 0.29 kcal/mol over the grid used for fitting).

Alternatively, multipoles of a given order can be represented by fixed charge arrangements, as is done in the distributed charge model (DCM).^356,357^ It replaces the evaluation of multipole–multipole interactions with the same number of charge–charge terms at the expense of introducing additional charge sites (Figure 8A). The magnitude *q*_*i*_ and position of the DCM charges are defined with respect to a reference atom. During fitting it may be useful to constrain the maximum displacement of the DCM charges. Reducing the number of interaction sites can be accomplished using differential evolution to optimize charge positions and magnitudes, and find arrangements that achieve a desired accuracy using a minimal number of charges (MDCM).^357^Figure 8B center and right show the ESP of a multipole representation and the corresponding 9-charge MDCM model for CCl_4_. More recently, the positions of the MDCM charges were explicitly coupled to the molecular geometry which leads to flexible MDCM (f-MDCM),^358^ available through the DCM module in CHARMM. It can effectively capture intramolecular polarization, and meaningful atomistic simulations can be carried out for condensed-phase systems. The latest development is kernel-based MDCM (kMDCM) that uses intramolecular separations as the features in a Gaussian-kernel to describe the charge displacements depending on molecular geometry.^359^

### 8.2 Machine-Learning-Based Energy Functions

Over the past few years, machine learning (ML)-based approaches have flourished for constructing PESs for molecular simulations.^360−363^ Typical approaches include permutationally invariant polynomials,^364,365^ neural networks (NNs),^54,366,367^ or kernel-based methods.^368−371^ The resulting PESs have been used for gas- and condensed-phase simulations to compute observables including spectroscopic properties and reaction rates.

PES representations based on reproducing kernel Hilbert space (RKHS) have long been used for small molecules.^370,372^ The TRIAKERN module in CHARMM provides the functionality to use such representations in MD simulations. The kernel coefficients **α** required for the RKHS-based PES are determined through a versatile external utility.^371^ Typical applications include reactive atom plus diatom collision systems,^373,374^ but the method has also been extended to spectroscopic investigations of larger molecules.^375^

The MLpot module^376^ in pyCHARMM^9^ allows running of mixed ML/MM simulations. It follows more established hybrid QM/MM strategies in which a usually smaller part of the system is treated with a QM method whereas the larger remainder is represented using an empirical energy function.^377−380^ In ML/MM, a ML representation, for example PhysNet,^54^ is combined via mechanical embedding with an empirical FF such as CGenFF available in CHARMM.^3,17,379^

The NN-PES computes the total ML energy and forces together with electrostatic interactions between the predicted fluctuating point charges of the ML-atoms and the static atomic charges of the empirical MM atoms. CGenFF^17^ handles energies and forces for the remaining MM atoms and van der Waals interactions between MM and ML atoms. Therefore, a set of van der Waals parameters must be assigned to the ML atoms. In PhysNet, charges of the ML atoms fluctuate depending on solute structure (intramolecular charge redistribution). This is akin to the fluctuating MDCM approach^358^ where geometry-dependent point charges reproduce the molecular electrostatic potential and describe intramolecular polarization. The advantage of mechanical embedding is the direct application of ML-based models of atomic systems trained in the gas-phase for condensed-phase simulations without additional training. Environment-dependent electrostatics can be included at the training stage by including solvent-surrounded solutes in the training set.

The input file to run pyCHARMM (Section 3.1)^9^ together with MLPot is a Python script. The MLPot module initializes an external model potential and evaluates potential energy and forces for the subset of ML atoms together with the CHARMM FF energy. By adapting the MLPot module in the source code, it is possible to link different model potentials such as ANI^381^ or SchNet.^366^ If the ML-based PES does not predict atomic charges, the electrostatic contribution between assigned static point charges of the ML and MM atoms are computed by the empirical energy function.

For chemical reactions that was long a domain of *ab initio* MD simulation, ML-based energy functions now provide means to run statistically significant numbers of trajectories (∼10^3^ or more) which was previously not possible.^382,383^ More recent examples include malonaldehyde in the gas phase,^384,385^ double proton transfer in hydrated formic acid dimer,^386^ or for atmospherically relevant reactions using permutationally invariant polynomials and NN-based energy functions.^382,387−389^

### 8.3 Multipole and Point-Induced Dipole (MPID)

CHARMM now supports advanced electrostatic interactions with multipole expansion up to hexadecapole via the developmental MPOLe module. Different real-space cutoffs can be applied for PME calculations, enabling the use of smaller cutoffs for higher-order multipoles. Additionally, it allows selective exclusion of specific multipole–multipole interactions, a feature utilized for the development of a water model.^390^ The module also facilitates the calculation of point induced dipoles. Several Thole damping functions, including those used in the AMOEBA^391^ and MPID^392^ FFs are supported. Moreover, the anisotropic atomic polarizability as utilized in the MPID model has been implemented. Induced dipole moments can be calculated either via full self-consistent field (SCF) relaxation or with an extrapolation scheme that uses weighted average of dipole moments after each of several cycles,^393^ where the third-order extrapolation with empirically optimized weighting coefficients (OPT3)^394^ is the default recommended method. See Section 10 for further explanation about CHARMM FFs.

### 8.4 Polarizable Intermolecular Potential Functions (PIPFs)

The point-dipole representation of the electronic response of a molecular system to an external field offers a systematic description of polarization effects within a classical framework, where several approaches can be equivalently derived.^338^ One example is the PIPF^395,396^ module of CHARMM.^397,398^ It complements other polarizable treatments available in CHARMM such as the fluctuating charge^399,400^ and Drude oscillator^401,402^ models.

In PIPF, each interaction site carries a fixed point charge and an inducible point dipole whose magnitude is determined by the total electric field due to all other point charges and induced point dipoles of the system. Assuming linear response, the induced dipole moment ***μ***_*i*_^ind^ at center *i* is proportional to the total electric field (**E**_*i*_^tot^) typically with a scalar isotropic polarizability α_*i*_,
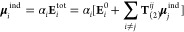
36The second part of Eq. 36 includes two contributions, the permanent electric field (**E**_*i*_^0^) due to fixed-point charges and the induced electric field due to induced dipole moments ***μ***_*j*_^ind^ at other sites. **T**_(*n*)_^*ij*^ is the rank-*n* polarization tensor.^338,403^

Thole’s interaction dipole (TID) model^404^ is employed in the PIPF model. Although an isotropic polarizability is used (a reasonable approximation for a non-interacting atom), the overall molecular polarizability is anisotropic. It has been shown that with the use of only a single parameter for each atom, the computed molecular polarizabilities using the TID model agree well with experimental data for molecules with a wide range of functional groups.^404,405^

Due to the interdependence of ***μ***_*i*_^ind^ in Eq. 36, a self-consistent iterative procedure is used in simulations to find them within a given threshold.^406^ The PIPF module in CHARMM includes three complementary approaches. The first is solving Eq. 36 by direct matrix inversion.^406,407^ Despite its *O*(*N*^3^) scaling behavior, exact results provided by direct matrix inversion are important for validating the convergence threshold for the faster iterative approach with *O*(*N*^2^) scaling. Direct matrix inversion is also needed for handling the intramolecular polarization part in the coupled polarization-matrix inversion and iteration (CPII) method explained below.

The next is to propagate induced dipoles dynamically via an extended Lagrangian.^408^ A Verlet integrator has been implemented to couple the fictitious dipole degrees of freedom to a low-temperature bath using the Nosé–Hoover thermostat.^409−412^ The low temperature dynamics makes the dipole fluctuations close to the true converged results. The extended Lagrangian method accelerates the self-consistent iteration scheme by nearly 2-fold.

The CPII approach involves a preconditioning algorithm. At convergence, the total polarization energy is
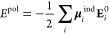
37The nuclear gradients can be obtained by differentiating Eq. 37 with respect to atomic Cartesian coordinates.^403,408^ On the other hand, when direct dipole dynamics is used, evaluating energy derivatives invokes additional terms containing **T**_(3)_(408) since induced dipoles are not at the variational minimum; those third order terms can be obtained in a compact form in CHARMM.^413^

To evaluate the permanent electric field, standard non-bonded list in CHARMM is used, where intramolecular atom pairs up to 1–3 bonded pairs (connected via a single atom) are excluded and 1–4 bonded pairs (connected via two atoms) are included. To alleviate spurious polarization interactions at short-range, Thole’s second damping function in the TID model^404^ is used by default to determine **E**_*i*_^0^ and **T**_(2)_^*ij*^.

Although excluding intramolecular polarization is practical for converging induced dipoles in MD simulations, works based on the TID model suggest that both intramolecular and intermolecular polarization should be included and damped in the same way to obtain consistent molecular polarizability. Unfortunately, including intramolecular polarization between bonded pairs can introduce numerical instability in the convergence of dipoles. While it can be avoided by matrix inversion, it is computationally too expensive for the entire system. The CPII method addresses this^398^ where iterative convergence of induced dipoles under intermolecular polarization is preconditioned using atom-distributed molecular polarizability tensor obtained from the TID model via matrix inversion of individual molecules. Consequently, Eq. 36 is modified to
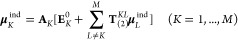
38where *M* is the number of molecules, *K* and *L* are indices for the corresponding atomic quantities grouped by molecules, and  is the atom-distributed molecular polarizability tensor^404,414^ which is in units of Å^3^ (see Eqs. 14 and 17 of ref (398)). The CPII preconditioning algorithm has been implemented in CHARMM and it accelerates dipole convergence in liquid simulations of amide and polypeptide systems.

In addition to gradients, second derivatives of PIPFs can be calculated in CHARMM with the point dipole formalism.^403^ With analytical Hessian available, PIPFs can be used in conjunction with the VIBRan module in CHARMM for vibrational FF analysis.

The PIPF model has been employed in MD simulations to examine polarization effects in a series of organic liquids including alkanes, alcohols, and amides.^395−397^ The results obtained with the classical point-dipole model were found to be in good agreement with those from combined QM/MM simulations in which polarization effects are described quantum mechanically.

Recently, the PIPF model has been employed in the doubly polarized QM/MM (dp-QM/MM) method to enhance the accuracy of SE-QM/MM (SE: semiempirical) free energy simulations.^415^ A well-known limitation of SE-QM methods is their tendency to underestimate molecular polarizability compared with experiments and AI/DFT-QM (AI: *ab initio*) benchmarks, leading to significant errors in free energy profile determined at SE-QM/MM levels. The dp-QM/MM method addresses this by improving the response properties of SE-QM/MM methods through high-level molecular polarizability fitting. Specifically, additional induced point dipoles are introduced on the QM atoms through a set of corrective polarizabilities (“chaperone polarizabilities”), whose magnitudes are determined from ML to reproduce the condensed-phase AI-DFT molecular polarizability along the MEP. These chaperone polarizabilities are then used in PIPF calculations in conjunction with QM/MM to compensate for the polarization energy underestimate in conventional SE-QM/MM simulations. Applied to the Menshutkin reaction in water, the dp-QM/MM method brought the computed and experimental free energy results into closer agreement.^415^

### 8.5 Long-Range Lennard-Jones Interactions

While the PME method^35,176^ for evaluating long-range electrostatic interactions was added to CHARMM in 1995,^416^ implementing the long-range Lennard-Jones (LJ) interaction lagged considerably. Long-range effects of dispersion are important for accurate calculation of free energies and interfacial properties of liquids and surfactants.^417−419^ Ignoring them leads to inconsistencies in the surface tension of lipid bilayers and monolayers.^420^ To address long-range LJ interactions, a lattice-based method termed LJ-PME^421^ was implemented into CHARMM.^422^ The name LJ-PME is arguably a misnomer, in that only the C6 (*r*^–6^) dispersion is calculated with an Ewald summation. The C12 (*r*^–12^) term continues to be truncated with a standard switching function.^4^ Dispersion-PME has been used interchangeably and is perhaps a better name.

Key to the efficiency of electrostatic PME is the simple multiplicatively separable functional form. While the analogous PME method for dispersion has been available for decades,^176,423^ its adoption has likely been hindered by the fact that many FFs, including CHARMM, use the Lorentz rule
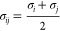
39to combine the LJ geometric parameters σ_*i*_ and σ_*j*_ for distinct atom types *i* and *j*. Since this does not yield a multiplicatively separable form, binomial expansion can be used:
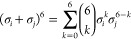
40However, this approach requires several PME evaluations in addition to those used in electrostatic PME. An elegant solution^421^ is to instead assume geometric mean combination rule
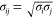
41and apply PME to the dispersion part of the LJ potential, requiring similar effort to that employed in the electrostatic term. This allows the C6 term to be calculated with a single PME calculation involving a simple multiplicative form, .

To correct for this approximation, the geometric-mean term is analytically subtracted (similar to how 1–2 and 1–3 exclusions are handled in electrostatics) for all pairs within a cutoff distance and substituted with the correct form, which may possibly include NBFIX pair-specific corrections (Section 10). The net effect is that the LJ potential is exact up to the chosen cutoff, beyond which the geometric-mean functional form is used. For an ∼8-Å cutoff, the geometric- and arithmetic-mean potentials coincide very closely and the overall approximation is excellent. To account for small but abrupt changes in energy as two schemes handoff at the cutoff, the original formulation in CHARMM applied a shift term to ensure continuity in energy. However this approach does not guarantee continuity in the first derivative. For this reason, a sigmoidal switch function has been implemented to seamlessly transition between the two regimes.^422^

The C6 terms are generally computed using the van der Waals parameters in CHARMM. To achieve greater flexibility, these values may be input directly regardless of atom types or short-range C6 values. In this way, specific interactions can be “fixed” to simplify parameter fitting and optimization by treating short-range and long-range dispersion terms independently.

The LJ-PME algorithm provided the impetus to revisit the use of cutoffs in the CHARMM lipid FF (Section 10.4). It has long been known that the high anisotropy present in such systems renders the standard isotropic corrections inappropriate. Because PME makes no assumption about the isotropy of the system, it is well suited to general systems including lipids. Previous iterations of the CHARMM lipid FF were parametrized with a given cutoff, with the cutoff errors implicitly absorbed into the parametrization. While successful, this strategy makes the resulting FF sensitive to the cutoff used at runtime; a choice other than that used for parametrization can yield erroneous results.

Long-range LJ terms can also be evaluated in CHARMM using the Isotropic Periodic Sum (IPS)^424^ and extended IPS methods.^417,418^ However, LJ-PME provides higher efficiency and transferability of FF among different simulation programs.

### 8.6 FACTS Implicit Solvent

The Fast Analytical Continuum Treatment of Solvation (FACTS) model is an efficient GB-based implicit solvent method for calculating the solvation free energy of proteins, protein complexes, and protein–ligand interactions.^132^ FACTS is based on the analytical evaluation of the volume and spatial symmetry of the solvent that is displaced from a solute atom by nearby atoms. For each solute atom, these two measures of solvent displacement are combined into an empirical sigmoidal equation for the calculation of the atomic (or self) electrostatic solvation energy and the solvent accessible surface area (SASA). The former is used to calculate the Born radii in the GB equation. The SASA is used to evaluate the non-polar contribution to solvation.

FACTS is fully analytical and because of its speed, it is useful for MD simulations. It has two main advantages over other implementations of the GB model. First, FACTS does not use the so-called Coulomb field approximation where the electric displacement field of a solute atom is calculated by assuming that the solute–solvent dielectric boundary is spherical with the atom at the center of the sphere. This assumption breaks down particularly for solute molecules with substantial aspherical volume and/or charges located close to the solute–solvent boundary. Second, FACTS does not require setting the dielectric discontinuity surface. Importantly, the CHARMM energy calculation with the FACTS model is only about four times slower than the vacuum energy, and FACTS scales linearly with system size (see Figure 11 in ref (132)).

FACTS is versatile as parameters for new or unknown atom types (e.g., non-proteinaceous atoms in organic compounds) can be generated automatically by interpolation from the existing FACTS parameters by the FACTS keyword TAVW. The effect of salt (ionic strength) is treated by the linearized Debye–Hückel approximation.^425^ The FACTS energy and force terms have been parallelized for multiple CPUs which provides substantial speed-up particularly for large systems. Furthermore, FACTS is compatible with the IMAGE module for periodic boundary condition (PBC) and the BLOCK module for energy decomposition. The INTE command of CHARMM can be used to evaluate the FACTS energy between two groups of solute atoms, e.g., a protein and a small-molecule ligand.

Since the original publication in 2008, FACTS has been employed in many simulation studies of (small) protein folding, (poly)peptide amyloid aggregation, and binding of ligands to proteins. One interesting example is the MD study of the interactions of the toxic Alzheimer’s Aβ_1–42_ peptide with carnosine, the endogenous brain dipeptide β-Ala-His, which revealed salt bridges with charged side chains, and van der Waals contacts with residues in and around the ^17^LVFFA^21^ central hydrophobic cluster of Aβ_1–42_.^426^ In 2014, the FACTS model was extended to lipid bilayer (membrane) environment by using a position-dependent dielectric constant and an empirical surface tension parameter. It was shown to reproduce the self-energy and pairwise interaction energies in solution calculated by the finite-difference Poisson method.^427^ However, the FACTS model for the membrane has not been implemented into the official version of CHARMM yet.

The last sentence of the original FACTS paper mentioned potential applications beyond MD: “*The accuracy and efficiency of FACTS suggest that it could also be used for protein structure prediction and docking*”.^132^ In fact, FACTS has been employed in several docking programs. A recent example is the FASTDock pipeline for the efficient scoring of poses in ortho- and allosteric pockets generated by MD.^127^ Another example is the docking protocol called Attractive Cavities which uses energy minimization and a smoothed potential energy for guiding small molecules into protein cavities. In a successive refinement step, the binding energy is calculated as the sum based on the CHARMM FF and the FACTS model.^428,429^ Concerning structure prediction, a Python tool allows for the evaluation of the FACTS total energy and its contribution as descriptors for ML models of protein–protein interactions.^430^

### 8.7 Implicit Modeling of Membranes

#### 8.7.1 Membrane Pores

**Figure 9:**
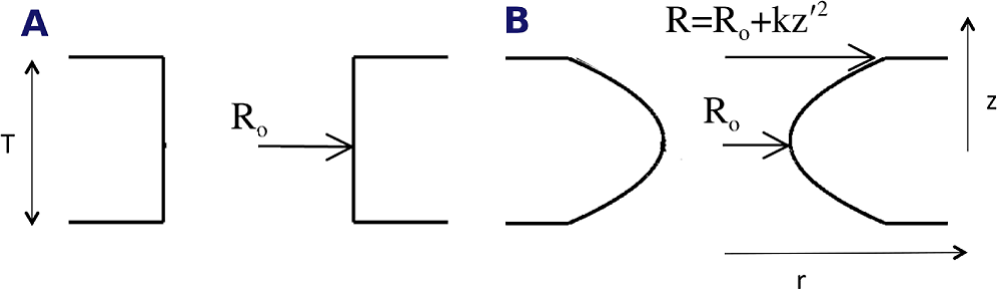
Illustration of pore shapes in IMM1. (A) Constant radius. (B) Radius depending on the *z*-coordinate.

The IMM1 implicit membrane model,^431,432^ an extension of the EEF1 effective energy function for soluble proteins,^433^ has been adapted to account for aqueous pores.^434,435^ IMM1 uses two sets of solvation parameters, one for water and one for the non-polar membrane interior. A continuous switching function *f* describes the transition from one environment to the other. Modeling of pores is accomplished by using a switching function *F* dependent on the vertical (*z*) and radial coordinate (the distance *r* from the *z*-axis). Denoting the thickness of the non-polar part of the membrane as *T* and the pore radius as *R* (Figure 9), the switching functions can be expressed using dimensionless variables:
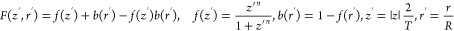
42Different pore shapes can be modeled by making *R* dependent on *z*, e.g., *R* = *R*_*o*_ +  (Figure 9B). Because the Gouy–Chapman formulas are invalid for pores in anionic membranes, an alternative approach is based on numerical solution of the Poisson–Boltzmann equation.^436^ It has been used to investigate the pore forming activity of antimicrobial peptides.^435,437−442^ More recently, this model was used for initial evaluation of putative structures of β barrel membrane pores formed by fibril-forming peptides and proteins, such as amyloid β, IAPP, and α-synuclein.^443−446^ A similar energy function has been implemented in the Rosetta protein design package.^447^

#### 8.7.2 Curved Membranes

The IMM1 implicit membrane model has been extended to spherical and cylindrical membranes (vesicles and tubes) by changing the definition of the relative depth *z*′ from |*z*|/(*T*/2) to |*r* – *R*|/(*T*/2) where *R* is the radius of the vesicle or tube and *r* the radial position of an atom.^448^ The model can also account for changes in lateral pressure profile as the membrane bends.^449^ It has been used to study ESCRT-III snf7,^450^ the mechanism of negative curvature generation by IBAR domains,^451^ and the interaction of caveolin oligomers with membranes.^452^

#### 8.7.3 Mean-Field Modeling of Deformable Membrane Bilayers via HDGB

The Heterogeneous Dielectric Generalized Born (HDGB) model^453^ is an extension of the GBMV implicit solvent model^160^ to capture the interaction of biomolecules with biological membranes. The most straightforward approach for implicitly modeling membrane–water interfaces is via a two-dielectric system where the membrane is modeled as a low-dielectric slab (ϵ = 1–2) embedded in a high-dielectric region (ϵ = 80). This idea was implemented in earlier implicit membrane models.^229,431,458^ The HDGB model refines it by introducing a continuously varying dielectric profile across the membrane–water interface to better describe the actual dielectric profile of membrane bilayers.^459^ The variable dielectric profile is then used in a modified GB equation
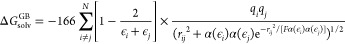
43where  (ϵ_0_: permittivity of vacuum) is a factor arising from units used in CHARMM, *N* is the number of atoms, ϵ_*i*_ is the variable dielectric profile at the position of atom *i* with charge *q*_*i*_, typically along the *z*-direction coinciding with the membrane normal,^453,460^*r*_*ij*_ is the distance between atoms *i* and *j*, α(ϵ_*i*_) are Born radii dependent on ϵ_*i*_, and *F* = 8 is a dimensionless parameter. In addition to a variable dielectric profile, the HDGB model introduces a SASA-dependent non-polar contribution:
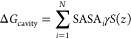
44where SASA_*i*_ is the SASA of atom *i*, and γ is the surface tension reflecting the strength of the non-polar term, and *S*(*z*) is an optimizable profile along *z* implemented as a spline-interpolated function. In principle, HDGB can be applied to any heterogeneous dielectric environment including those with non-slab geometries. For example, spherical micelles can be modeled by varying the dielectric and non-polar profiles as a function of the radial position from the center. It is also possible to exclude select atoms from the variable dielectric and non-polar profiles, which allows modeling of membrane channels where membrane-facing atoms would be in contact with water or ions instead of the lipid bilayer.

HDGB variants available in CHARMM are summarized in Table 2. In the original HDGB,^453^ the dielectric profile was taken from Poisson–Boltzmann calculations for a probe sphere at different locations in a multilayer dielectric system and the non-polar profile was adjusted to match insertion free energies of model molecules. In HDGBv2,^454^ both dielectric and non-polar profiles were optimized further to improve insertion free energies of amino acid analogues. In HDGBv3,^455^ side-chain interactions within the membrane from all-atom simulations were taken into account. HDGBvdW^456^ includes implicit van der Waals interactions inside and outside of the membrane separate from the cavity non-polar term. This further improved intramembrane interactions. Furthermore, DHDGB^457^ adds dynamically fluctuating membrane deformations by coupling HDGB to membrane deformation energies from elasticity theory. This allows for a more realistic modeling of charged and polar compounds near and inside the membrane bilayer via membrane deformations. The DHDGB model can be combined with any other HDGB models in Table 2.

**Table 2:** HDGB Model Variants Implemented in CHARMM^a^

Model	Dielectric Profile	Non-Polar Profile	VDW Term	Deformable
HDGB^453^	PB calculations for multilayer dielectric	Insertion profiles of O_2_ and water	No	No
HDGBv2^454^	Δ*G*_insert_ for amino acid analogues		No	No
HDGBv3^455^	Intramembrane interactions and Δ*G*_insert_ for amino acid analogues		No	No
HDGBvdW^456^	Same as HDGBv3	Reoptimized as for HDGBv3	Yes	No
DHDGB^457^	Any	Any	No	Yes

a PB, Poisson–Boltzmann; Δ*G*_insert_, insertion free energy of amino acid analogues; VDW, van der Waals.

With HDGB, it is possible to study a variety of peptide–membrane interactions. A significant advantage of an implicit membrane model is that slow bilayer reorganization kinetics can be avoided. This is especially relevant for peptide–membrane insertion where atomistic simulations may be too slow to converge. HDGB has been used successfully to study the insertion of viral fusion peptides.^461,462^ Another advantage is that the width of the bilayer can be easily varied simply by scaling the dielectric and non-polar profiles. This allowed a comparison of phospholamban conformational sampling in different physiologically relevant bilayers.^454^ It also led to a method for estimating the optimal membrane width based on the structure of a given integral membrane protein.^463^

HDGB was also used to estimate the membrane permeability of drug-like molecules,^464^ and as a scoring function for membrane protein structures,^465^ which led to a MD-based structure refinement protocol for integral membrane proteins.^466^ HDGB can also be used for simply simulating the dynamics of membrane-embedded integral proteins.^467^ However, for larger systems, the computational advantage of HDGB over explicit lipid simulations is not as significant.^468^

### 8.8 Transferable Coarse-Graining via PRIMO

**Figure 10:**
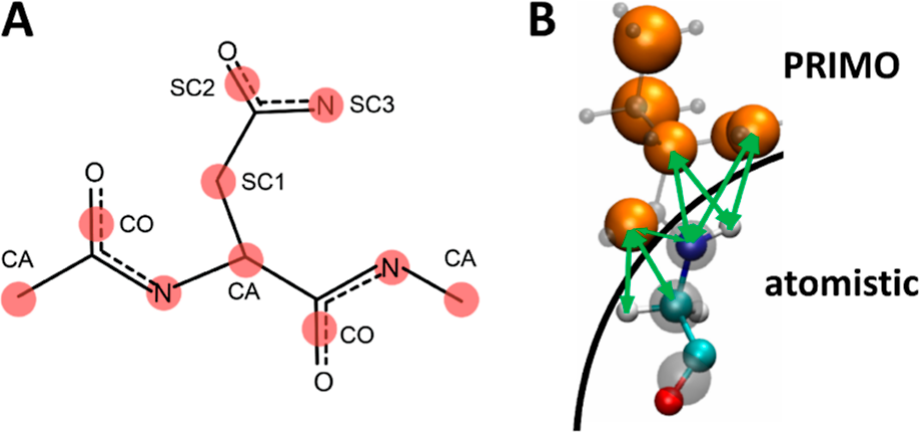
Illustration of the PRIMO CG model. (A) PRIMO interaction sites (red spheres) for asparagine as an example. (B) Hybrid all-atom/CG model of a protein with coupling between a PRIMO region and an atomistic region.

The Protein Intermediate Resolution MOdel (PRIMO) is a CG model for proteins and nucleic acids with resolution intermediate between atomistic and residue levels.^469^ In PRIMO, protein backbones are represented with three particles: C_α_, N, and CO (at the midpoint of the carbonyl group). Non-glycine side chains are represented with one to five beads depending on the size of the side chain (e.g., Figure 10A). Nucleic acids are represented with a similar level of coarse-graining.^469^

The CG sites in PRIMO were chosen to allow an analytical reconstruction of atomistic detail with minimal loss of accuracy by applying known standard bond geometries.^469^ On average, all-atom reconstructions from PRIMO deviate by only 0.1 Å from the original all-atom models.^469^ For comparison, at the time PRIMO was developed, all-atom reconstructions from C_α_-only models deviated by 1.7 Å^469^ on average and even when C_α_ sites were combined with a site at the side chain center, the reconstruction error remained at 0.9 Å.^469^ More accurate all-atom reconstructions from residue-level CG models up to 0.5 Å are now possible with advanced ML models,^470^ but PRIMO has an advantage by maintaining very close connection to atomistic models with significantly reduced number of interaction sites. The near-exact mapping between CG and atomistic levels in PRIMO can be used to compress all-atom trajectory data.^471^

The PRIMO FF incorporates a combination of bonded and non-bonded terms in all-atom FF.^472^ It augments standard bonded terms with spline-based functions because interactions between many of the CG sites have multiple minima that are not approximated well with single-well harmonic terms. Moreover, some bonded terms operate on virtual sites that are reconstructed on-the-fly from the CG sites, which is possible because of the computationally efficient analytical mapping from PRIMO to atomistic sites. The virtual site approach is also used for an explicit hydrogen-bonding potential using 2D spline interpolations of PMF as a function of hydrogen bond angle and distance similar to the CMAP torsion potential in CHARMM.^473^ Finally, PRIMO captures solvation effects via implicit solvent terms. A GBMV-based model^160^ is used to capture electrostatic contributions to the solvation free energy. It is complemented with a per-residue SASA term that adds non-polar contributions and compensates for incomplete electrostatic solvation contributions with the GB model because of less polarized PRIMO interaction sites. By replacing GBMV with the HDGB implicit membrane model,^453^ PRIMO can be extended to simulate protein–membrane interactions.^474^ The PRIMO FF were parametrized primarily by matching energies from CHARMM’s all-atom FFs, in particular CHARMM22/CMAP^475^ and CHARMM36^476^ with further adjustments made based on simulations of test peptides.^472^

The PRIMO FF is fully transferable to other systems.^477^ It is possible to run stable MD simulations of arbitrary protein systems without restraints^472,474^ and PRIMO can be used in combination with enhanced sampling techniques in peptide folding simulations,^472^ or to study the insertion of peptides into membranes.^474^ PRIMO is also useful for extensive conformational sampling in protein structure refinement.^478^

Because PRIMO and atomistic interaction potentials are similar and compatible with each other, it is possible to run hybrid multiscale simulations where parts of a system are represented by PRIMO whereas other parts are represented in atomistic detail. One example is the simulation of a peptide in atomistic detail surrounded by crowder molecules represented at the CG level using PRIMO,^479^ where the coupling between the CG and atomistic levels involves only the non-bonded and solvation terms. It is also possible to run multiscale simulations where different parts of the same molecule are represented either atomistically or via PRIMO.^480^ The coupling between the all-atom and CG levels extends to the bonded terms by maintaining dual resolution across the interface between CG and atomistic regions. PRIMO resolution is trivially obtained from the atomistic level whereas atomistic sites are reconstructed analytically from the PRIMO model (Figure 10B).^480^ Using this approach, it is possible, for example, to efficiently sample dynamic regions of a given system at the CG level while applying atomistic detail to maintain accuracy of more conserved structural elements.^480^

PRIMO is unique because of its close coupling to the CHARMM all-atom FF. Its advantage is a high degree of transferability compared to other CG models and a suitability for multiscale simulation approaches where a given system can be represented simultaneously at different levels of resolution. The CG nature of PRIMO improves computational efficiency over comparable all-atom simulations with the same GBMV implicit solvent model by about a factor of 10 in wall-time.^472^ There are additional gains in efficiency due to the smoother energy landscape at the CG level. However, the use of the relatively expensive GBMV model limits PRIMO’s overall performance, especially for larger systems where the advantage of implicit solvent over explicit solvent diminishes.^468^ Combining PRIMO with other less expensive implicit solvent models in CHARMM is an option for potentially overcoming these limitations.

## 9 Specialized Restraint Methods

Restraint energy functions apply biases on particular degrees of freedom, and can drive the system into conformational states that may be otherwise inaccessible, thereby improving sampling around those states.

### 9.1 CONSHELIX Module

**Figure 11:**
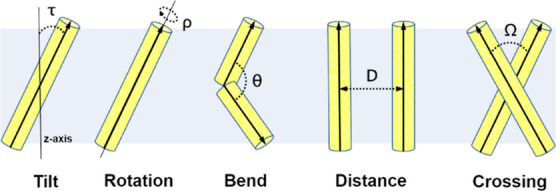
Helical reaction coordinates.^481,482^ The tilt angle τ is relative to the *z*-axis; the rotation angle ρ is measured for a designated atom about the helical axis; the bend angle θ is between two helical axes; the minimum distance *D* and crossing angle Ω are between two neighboring helices. Corresponding restraint energy functions are handled by the CONSHELIX module in CHARMM. For a hairpin, tilt, rotation, and distance restraint potentials are also available.

Most restraint potentials control reaction coordinates between atoms, such as CONS HARM, NOE, and RESD for atoms, and CONS DIHE for dihedral angles in CHARMM. By comparison, the restraint potentials in the CONSHELIX module are applied to molecular-level reaction coordinates such as helices and hairpins (Figure 11). These energy functions are especially useful for controlling motions of transmembrane domains within the lipid bilayer,^483,484^ which are critical for, e.g., signal transduction,^485,486^ transport of ions and small molecules, antimicrobial activity, and transmembrane responses.^487^

The CONSHELIX restraint potential *U*_ξ_(**R**) (Eq. 45) takes a quadratic form for non-periodic variables (ξ = τ, θ, *D*, or Ω; defined in Figure 11) and a cosine function for periodic variables (ξ = ρ), where **R** represents coordinates of atoms selected to define the helix/hairpin principal axis. Denoting the force constant and the target value as *k*_ξ_ and ξ_0_, respectively,
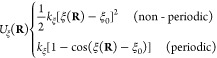
45An example application is the study of the mismatch in the thickness of the hydrophobic region between the protein and the lipid bilayer,^487^ which leads to changes in lipid length, tilting of transmembrane proteins, and association of multiple transmembrane proteins. To explore the resultant tilting motion of the WALP19 model helix peptide (sequence: GWW(LA)_6_LWWA) in a DMPC bilayer, umbrella sampling was performed.^488,489^ The weighted histogram analysis method (WHAM) and TI were utilized to obtain the PMF as a function of the tilt angle τ, where ‘precession entropy’ was proposed as the driving force for tilting. As τ increases, the accessible volume of the helix conformation also increases (precession entropy), which stabilizes the helix in a tilted orientation.

Another application is the association free energy between two helices using the distance *D* in a DMPC bilayer.^490^ The asparagine residue at the center of the helix drives helix–helix association in a membrane by forming bifurcated hydrogen bonding. However, the interaction between the helix and the lipid promotes dissociation of the two helices. The free energy cost for lipid depletion between helices is relatively small, around 7.6 cal/[mol·Å^3^], compared to that for cavity formation in water, 24–33 cal/[mol·Å^3^]. Through TI, various energy terms within a residue can be decomposed, which inform residues that favor helix–helix or helix–lipid interactions.

### 9.2 SSNMR Module

Solid-state NMR (SSNMR) spectroscopy is used to determine membrane protein structures in a native-like membrane environment. It utilizes 2D ^15^N–^1^H NMR polarization inversion spin exchange at magic angle (PISEMA) spectrum experiments to obtain orientational information from dipolar coupling and chemical shift. To harness this experimental data effectively, the SSNMR module has been implemented in CHARMM.^491^

Experimental observables (*O*_ssnmr_) obtained through the SSNMR spectroscopy are dipolar coupling (ν) for ^15^N–^1^H pair and chemical shift (σ) for ^15^N atom:
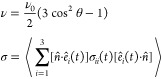
46Here, ν_0_ ≡ γ_*N*_γ_*H*_*hμ*_0_/(8*πr*^3^) is the dipolar coupling constant, where γ_*N*_ = −2.71 × 10^7^/(T·sec), γ_*H*_ = 2.675 × 10^8^/(T·sec), *h* is the Planck constant, μ_0_ is the vacuum permeability, and *r* is the N–H bond length. θ is the angle between the N → H bond vector and the direction n̂ of the magnetic field that is assumed to be normal to the membrane.  (*i* = 1, 2, 3) are the instantaneous basis vectors for the chemical shift tensor where σ_*ii*_ is its diagonal element.  and  are on the plane spanned by C, H, and N atoms of the peptide bond, and .^491−493^

**Figure 12:**
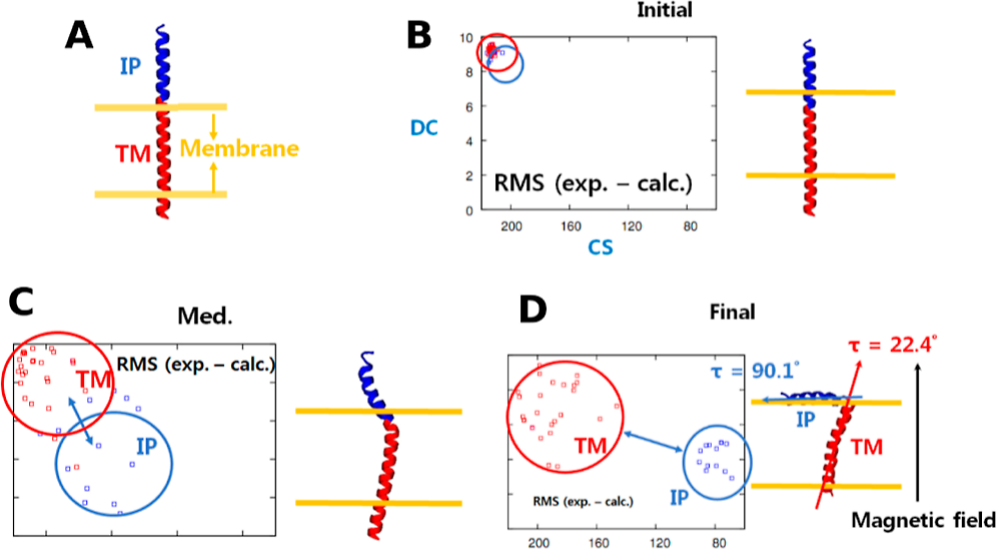
Structural changes of the fd-coat membrane protein through MD simulations with the SSNMR restraint potential. (A) The fd-coat system. IP (blue): In-plane helix; TM (red): Transmembrane helix. (B) Distribution of chemical shift (*x*-axis) and dipolar coupling (*y*-axis) calculated for the initial state structure. (C) Structure and calculated distribution in an intermediate stage of the simulation. Note changes in the distributions and conformations. (D) The final stage of the simulation. IP is at the lipid–water interface, while TM is positioned within the membrane.

Due to cos^2^ θ in Eq. 46, for one dipolar coupling experimental value, up to four helix orientations are possible, potentially with multiple helical structural conformers in membrane (‘structural ambiguity’). The simplest form of restraint energy *U* is a quadratic function that minimizes experimental values
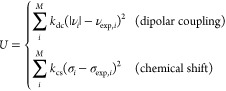
47where *M* is the number of restraint potentials and the subscript ‘exp’ denotes experimental value. Eq. 47 can be minimized through various methods such as MD, MC, genetic algorithm optimization, and simulated annealing. A structure that satisfies experimental data can thereby be obtained. An example is modeling the structure of the fd-coat, the major pVIII coat protein of the fd filamentous bacteriophage (Figure 12). It has two types of α-helices within the lipid membrane: the amphipathic in-plane (IP) helix at the water–lipid interface, and the longer transmembrane (TM) helix. Solution and solid-state NMR structures are available (PDB ID: 1FDM and 1MZT).^494^ Calculations were performed in vacuum while considering a virtual lipid environment where Eq. 47 was used with experimental values as minima. Initially, the structure is perpendicular to the lipid membrane. Subsequently the IP region adheres to the lipid membrane surface, and the TM region adopts a tilted structure to match the hydrophobic length of TM and the membrane thickness (Figure 12), which satisfy constraints based on experimental data.

Other applications of the SSNMR module on transmembrane proteins include MerF (a mercuric ion transporter), M2 (transmembrane domain from influenza A virus), and Vpu (viral protein u from HIV-1).^491^ In another application, the SSNMR module was combined with the ensemble dynamics (ED) technique^495−498^ in an explicit membrane system to address discrepancies between semistatic and dynamic fitting models of SSNMR observables. Compared to these two fitting approaches, the main advantage of the SSNMR ED is its ability to generate an ensemble of structures (e.g., TM helix orientational distribution) that satisfy experimental observables within a reasonable physical model, without prior knowledge about the underlying distribution or motion.

### 9.3 Residual Dipolar Coupling (RDC) NMR Orientational Restraint

**Figure 13:**
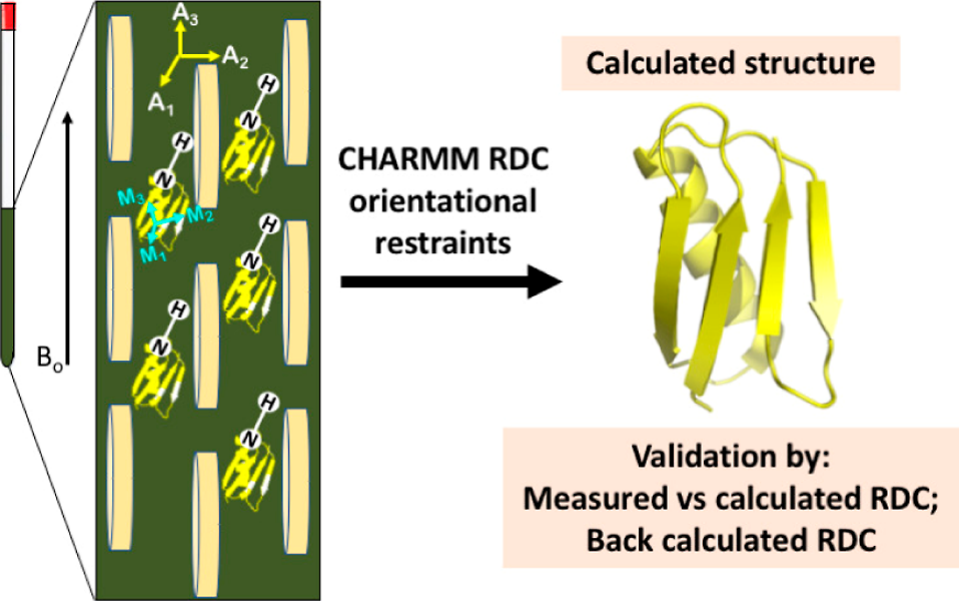
From RDC (*D*_PQ_) measurement to structure calculation using CHARMM illustrated by using the N–H internuclear vector of protein G (PDB 1P7E).^500^ The protein solution in the NMR tube is indicted in green. Oval-shaped slabs indicate the alignment media. **B**_0_ is the applied static magnetic field, and **A**_*i*_ and **M**_*i*_ (*i* = 1, 2, 3) represent the 3 principal axes for the alignment tensor and the inertia tensor of the molecule, respectively.

The RDC module in CHARMM leverages experimental time-averaged RDC orientational NMR restraints (Figure 13).^499^ RDC informs about the orientation of each internuclear vector **r**_*PQ*_ = **r**_*P*_ – **r**_*Q*_ formed by a pair of NMR active nuclei P and Q in a molecule with respect to the static magnetic field **B**_0_. CHARMM uses decoupled RDC orientational information^500^ that consists of (1) the angle ψ_*i*_ between **B**_0_ and the *i*-th principal axis of the inertia tensor of the molecule, **M**_*i*_, and (2) the angle θ_*i*_ formed between **r**_*PQ*_ and **M**_*i*_. The RDC between the two nuclei is
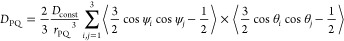
48where *D*_const_ = −*Sμ*_0_γ_*P*_γ_*Q*_*h*/8π^3^, where μ_0_ is the magnetic permeability of vacuum, γ_*P*_ and γ_*Q*_ are gyromagnetic ratios of the nuclei *P* and *Q*, *h* is the Planck constant, and *S* is the generalized order parameter that describes the internal motion of the internuclear vector. The angular brackets indicate time average. Eq. 48 can be expressed in terms of the alignment tensor or Saupe order matrix *A*
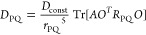
49where *O* consists of the three principal axes (eigenvectors) of **M**_*i*_ and the 3 × 3 matrix *R*_PQ_ = **r**_PQ_ ⊗ **r**_PQ_. Since *A* is traceless, only 5 components are independent, which can be determined by using singular value decomposition (SVD) with the aid of experimental RDC,^501^*D*_*n*×1_^exp^ (*n*: number of experimental values) and **M**_*i*_(500)
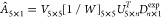
50where *V*, 1/*W*, *U* and *D* are matrices arising from SVD.

CHARMM supports simultaneous use of RDCs measured between different NMR active nuclei (e.g., N–H, C_α_–C, and C–N). Since RDC provides only the orientational information, it alone cannot fully determine the structure of a biomolecule. Thus, RDC and other NMR restraints such as NOE (via the NOE keyword) are used together. Alternatively, one can use a fragment-based approach^502^ to determine molecular structure using multiple RDCs collected in multiple alignment media. It is recommended to “reset” the RDC module before using it for structure determination.

The module requires the name of the input file(s) containing experimental RDC values, their upper and lower bounds, and the corresponding internuclear atoms. Since the CHARMM RDC module uses the harmonic potential with soft asymptotic behavior, for each RDC, one can specify the force constant for the harmonic potential and the slope for the asymptotic function (default = 1), the value for the exponential function used in the soft asymptote if it is other than 1, and the cutoff length for the harmonic function (default: 1 Å). The input files may contain RDCs collected in different alignment media, stored in the CHARMM (default), BMRB, or XPLOR format. Other command options include the maximum RDC restraints, whether the RDCs (other than for N–H) need to be scaled with respect to the N–H RDC, and whether the principal axes are calculated only with respect to the RDC atoms. For validation, users can compare experimental versus calculated RDC data and back-calculate other types of RDC (e.g., C–N and/or C_α_–C) for the structure based on the input RDC used (e.g., N–H).

### 9.4 Torque Application

Application of a torque on selected atoms about a user specified axis can be achieved by the PULL TORQue command.^503^

## 10 CHARMM Force Field Development

Since the publication of the second CHARMM paper in 2009,^3^ significant progress has been made in the CHARMM-related FFs. While the additive CHARMM36 (C36) FF was mature at that time, additional enhancements and refinements, including creation of CGenFF, were performed. With respect to polarizable FFs, major developments in the classical Drude oscillator model were made including coverage of all the major classes of biomolecules and progress was made toward a Drude General FF (DGenFF). A detailed description of the potential energy functions for the additive and Drude FFs is presented in ref (23).

In addition, a polarizable model based on MPID (Section 8.3) onto which Drude FF-based parameters can be mapped was presented.^392^ To avoid the costly SCF procedure (Section 8.3), a small mass is assigned to the Drude particles, which are then propagated as dynamic variables during simulations via a dual-thermostat extended Lagrangian algorithm, with a “cold” temperature imposed on the degrees of freedom corresponding to the induced dipole. The statistical mechanical validity of this procedure was clarified.^504^ In addition to CHARMM, the additive and Drude FFs are available in OpenMM,^505^ facilitating the CHARMM/OpenMM API (Section 2.1), as well as in GROMACS^506^ and NAMD,^507^ though the implementation of the Drude FF in GROMACS is currently limited, and NAMD currently does not include LJ-PME capabilities (Section 8.5).^421^ Notable is the ability to setup and generate inputs for complex molecules for a range of programs using CHARMM-GUI^87^ (Section 3.3), including for the Drude polarizable FF.^115^ An advantage of the Drude FF over other polarizable FFs is the computational efficiency, i.e., it is only about 4 time slower to run compared to additive FFs.

### 10.1 Water, Ions, and Polar Solvents

A number of extensions of the Drude FF, including additions to the potential energy function, have been made with respect to water and ions since 2009. Extensions to the additive FF include the use of alternative LJ interactions between the CHARMM TIP3P water model and proteins^144^ (Section 10.2), and revised LJ parameters for Na^+^ and Ca^2+^ with lipids.^508,509^ The default water model with the Drude FF is the SWM4-NDP (simple water model with 4 sites and negative Drude polarization) model,^510^ though a 6-point model SWM6 with an improved condensed phase hydrogen bonding properties is available with an increased computational cost.^511^ The energy function was expanded to account for polarization anisotropy to describe the dielectric constant of liquid amides more accurately.^512^ Substantial work was undertaken on the ions in the Drude FF, including a range of monatomic ions^513,514^ and later for molecular ions, including a number of ions uncommon in biomolecular systems.^515−517^

Developing the parametrization for all charged moieties made it possible to adopt a consistent absolute solvation scale for monatomic and molecular ions.^513,515^ Notable with the optimization of Mg^2+^ was the use of an LJ repulsion between the SWM4 Drude particle and the Mg^2+^ ion.^517^ This enabled steric repulsion between water and ion as the water polarizes in the electric field of the ion. This avoids overbinding and yields a model that can reproduce both the experimental thermodynamic and kinetic properties, a capability not attained in any other FF to our knowledge. It highlights the importance of explicitly including electronic polarizability in a FF as well as the advantage of using a Drude oscillator particle in representing the electronic degrees of freedom. It also captures the cation−π interactions for aromatic side chains more accurately.^518^ As parametrization of ions and charged molecules is typically carried out to reproduce experimental data in the infinite dilution limit, accounting for the osmotic pressure made it possible to extend the models to concentrated solutions via pair-specific LJ (NBFIX) and Thole electric shielding (NBThole).^519^ A similar philosophy was exploited to optimize the parametrization of amide solutions.^520^ Early efforts led to a preliminary set of optimized parameters for ion–protein interactions,^521^ although additional tests revealed a number of issues that are currently being addressed.

### 10.2 Proteins

Developments in the C36 additive protein FF have involved two iterations building on the C27 FF, also known as C22/CMAP.^473^ The revisions primarily involved optimization of the bonded parameters with only minimal changes in non-bonded terms.^476^ The first iteration published in 2012 yielding C36 focused on the CMAP term targeting NMR solution data for non-Gly, non-Pro amino acids, the CMAP terms for Gly and Pro residues targeting high-level QM data and the χ_1_ and χ_2_ side-chain dihedral parameters, with the latter targeting condensed phase data from simulations of (Ala)_4_-X-(Ala)_4_ model peptides.^522^ Subsequent focus was on the CMAP term to account for oversampling of the α_L_ conformation with C36 and improvements in the interaction between Arg and carboxylate groups using an off-diagonal LJ term (NBFIX in CHARMM nomenclature), yielding C36m.^144^ These additional optimizations lead to improved treatment of intrinsically disordered peptides (IDPs) while maintaining accurate treatment of folded proteins. C36m is considered the default FF for additive protein simulations.

An interesting outcome of that study regarded the role of LJ interactions between the water molecule and the protein in the sampling of folded versus unfolded states of IDPs. While changing the magnitude of the water–protein interactions could improve the equilibrium between folded and unfolded states for a specific protein, a general solution that universally treats all IDPs in the context of an additive FF may not be accessible. Additional works included improved treatment of cation−π interactions,^523,524^ and halogen–protein interactions important for ligand–protein simulations.^525^ Furthermore, the additive FF was extended to over 100 non-standard amino acids^526^ and to α-methyl amino acids.^527^

Advances in the Drude FF have been substantial as prior to 2009 only water, ion, and small molecule parameters had been published. Parameters have been released for many other biological molecules, including significant work on the protein portion of the FF. Small molecule developments included heteroaromatics,^528^ sulfur-containing compounds,^529^ and ethers^530^ with the Drude FF shown to yield accurate hydration free energies facilitated by the use of atom-pair-specific LJ parameters (i.e., NBFIX).^531^ Building on the foundation of the small molecule parameters the first generation of the Drude protein FF, Drude-2013, was presented. It overcame challenges with moving from individual molecules to a polymer in a polarizable FF, where unexpected overpolarization was avoided by accounting for the conformational properties of the polypeptide backbone.^532^

Application to polypeptide simulations revealed importance of explicit polarization in both peptide folding^533^ and unfolding,^534^ by capturing cooperativity inaccessible to additive FFs. A number of issues found in Drude-2013 including the stability of β-sheet structures, led to additional optimization yielding Drude-2019.^535^ Improvements involve both the polypeptide backbone and side-chain conformational properties including optimization of the electrostatic parameters of the atoms linking side chains to the backbone, as well as the treatment of cation−π and anion−π interactions.^536^ Drude-2019 shows systematic improvements as compared to C36m and Drude-2013, and it allows stable simulations of proteins on the microsecond time scale. While the Drude FF has largely been developed assuming an explicit solvent model, a Poisson–Boltzmann (PB) implicit solvation model has been developed^537^ and subsequently used to predict p*K*_a_ values of proteins.^538^ With the Drude PB model, p*K*_a_’s calculated for 8 proteins were insensitive to the assigned dielectric constant, in contrast to the need for a value of 4 with C36m. This indicates a potential advantage of the polarizable model in implicit solvent approaches.

### 10.3 Nucleic Acids

Both additive and polarizable FFs for nucleic acids have been updated. Updates to the C27 nucleic acid FF include improved treatment of RNA, largely to account for contributions of the 2′OH group to conformational heterogeneity^539^ as reported in a combined QM/bioinformatics study.^540^ Work on DNA focused on the equilibrium between the BI and BII conformations in duplex structures.^541^ In both cases, adjustments to the C36 FF only involved select dihedral parameters, suggesting that minimal improvements within the non-polarizable additive approximation were necessary. Beyond canonical DNA and RNA, the C36 FF was extended to a range of naturally modified ribonucleotides as required for the ever increasing list of non-coding RNAs.^542^

Given the high charge density of polyanionic nucleic acids, a polarizable model is of particular interest. Development of the Drude FF was based on carbohydrate, ion, and heteroaromatic parameters. The first step involved optimization of Drude parameters for nucleic acid bases targeting a range of QM data for interactions with water and for base–base interactions, as well as experimental data including base crystal geometries and heats of sublimation.^543^ They were then combined with initial parameters for the phosphodiester linkage yielding the Drude-2013 DNA model^544,545^ that was iteratively optimized with particular emphasis on dihedral parameters associated with phosphodiester, sugar, and sugar–base glycosidic linkages. The optimization involved comparison with experiments including crystal data, to ensure suitability in simulations of duplexes in the condensed phase such as the equilibrium between A- and B-DNA and the BI/BII forms. This initial model improved agreement with experimental data regarding base flipping,^546^ and yielded insights into the distribution and competition between ions around duplex DNA.^547^ Impact of ions on DNA conformation including the minor groove width could also be addressed.^548^ Such results cannot be captured well by additive FFs, again emphasizing the utility of the polarizable model for studying charged species.

Subsequent optimization of the Drude-2013 DNA FF focused on the underestimation of base stacking in duplexes and unwinding of Z-DNA. It involved additional QM calculations on Z conformations and application of higher-level model chemistries for other QM data.^549^ The resulting model reproduced both crystal and solution scattering data over a range of duplexes in microsecond simulations.^550^ The FF was also extended to RNA^551^ which focused on the role of the 2′OH group on the conformational properties using QM data on RNA-specific model compounds. Condensed phase testing involved stem-loop structures, adenine riboswitch, and canonical duplexes, showing good agreement with crystallographic and NMR data.

The combination of the DNA and RNA FF, termed Drude-2017, was applied successfully to a number of systems including quadruplexes where the ions in the G tetramer are stabilized by the explicit inclusion of electronic polarizability.^552^ However, a tendency of the Drude-2017 FF to overpolarize the Drude particle during MD simulations was noted. While this was addressed by using the Drude hardwall constraint^553^ it represents a non-adiabatic condition. The electrostatic parameters were subsequently adjusted to yield a model that was successfully used in simulations of RNA hairpins.^554^

### 10.4 Lipids

Significant advances were made to the additive and polarizable lipid FFs. Revised parameters for 6 neutral lipids were introduced, yielding the C36 lipid FF.^555^ As background, previous CHARMM lipid FF^555^ required an applied surface tension to avoid unphysical bilayer surface area contraction in NPT simulations. Adjustments to charges and torsion angles in the headgroup region in C36: (1) reduced the surface tension to zero at the observed experimental surface area per lipid for free-standing dipalmitoylphosphatidylcholine (DPPC) bilayers; (2) increased area compressibility to experimental ranges; and (3) captured the experimentally observed splitting in deuterium order parameters for carbons in glycerol and carbon 2 of the chain 2. The C36 FF was further validated by the agreement with experimental bending constants^556^ and spontaneous curvatures^557,558^ (those for bilayers required new code for pressure profiles described in Section 12.3). Extensions to new lipids are ongoing. To date, common phospholipids are parametrized, including 13 variants of inositol lipids, sphingolipids, 5 hydroxylations for ceramide lipids, ether lipids, glycolipids, and acyl chain variants (saturated, monounsaturated, polyunsaturated, branched, and cyclic). Excluding the nearly unlimited variations in glycolipids and lipopolysaccharides, over 300 lipids have been parametrized for C36^559−561^ and they are readily available in CHARMM-GUI. A united-atom representation wherein hydrogen atoms are combined with their bonded heavy atom, has also been formulated and tested for most common lipids (C36UAr).^562^ It is currently being extended to other lipid head groups and chain types such as sphingolipids.^563^

Despite its extensive refinements and wide usage, C36 has two fundamental limitations: sensitivity of the truncation method used for the LJ interactions, and lack of polarizability. The former manifests as inconsistent bilayer and monolayer surface tensions, which is because bilayers are parametrized to agree with their experimental surface area at particular temperatures. The acyl chain–air interface of monolayers is highly sensitive to truncation of the LJ potentials, causing underestimation of surface tension when using the same parameters that otherwise yield accurate results for bilayers. Conversely, the surface tension of the C36 DPPC monolayer agrees well with the experimental value when long-range LJ terms are included, but the bilayer contracts.^420^ It was thus necessary to parametrize the bilayer and monolayer consistently. While it can in principle be carried out with a truncated LJ potential, it is physically more reasonable to parametrize both with long-range interactions, which also avoids the sensitive dependence of bilayer properties such as for phase changes, on user-specified cutoff values. While a computationally efficient way of including long-range LJ terms in anisotropic systems such as bilayers and monolayers was unavailable when C36 was developed, subsequent incorporation of LJ-PME^421,422^ (Section 8.5) led to reparameterizing C36 to C36/LJ-PME.^564,565^ Consistency of bilayer and monolayer surface tensions for DPPC in C36/LJ-PME was obtained without compromising the overall quality of C36 for bilayers. Yet, monolayer isotherms at very large surface area where the surface tension of water–air is important are not well-described in C36/LJ-PME because the water–air surface tension of TIP3P (the default water in the additive CHARMM FF) is substantially lower than the experimental value.^417^

The second limitation of C36, the lack of polarizability, manifests as water permeability in saturated lipids being 5-fold lower than experimental values.^566^ This is because the transfer free energy of water into hexadecane (a good model for the interior of a bilayer) is overestimated by 1 kcal/mol. Also, the dipole of the additive water cannot readjust when it is in the lipid environment. This motivated the development of the CHARMM Drude polarizable FF. Early versions of the lipid Drude FF^553,567,568^ provided insight into membrane dipole potentials,^567^ the mechanism of permeation of arginine as a function of membrane thickness,^569^ as well as ion conduction along the narrow gramicidin A channel.^570^ While these studies demonstrated the importance of a polarizable FF for membranes, the initial parametrization of phospholipid molecules had a number of shortcomings, including overestimated bilayer area compressibility. Furthermore, it was optimized with a truncated LJ potential without accounting for long-range dispersion. Bilayer surface areas and compressibility of the revised Drude-2023 FF^571^ agree much better with experiments. More importantly, Drude-2023 yields more accurate dipole potentials, water permeability, monolayer isotherms, and lipid diffusion constants compared to C36 or C36/LJ-PME. Efforts toward a more comprehensive collection of lipids in the context of Drude FF, including charged lipids and ceramide-based lipids, are ongoing.

### 10.5 Carbohydrates

Building FF for carbohydrates poses a particular challenge given the wide range of monosaccharides, including both furanoses and pyranoses, the large number of chemical functional groups beyond hydroxyls, and various glycosidic linkages in poly- and oligosaccharides. Additive carbohydrate FF developments since 2009 included acyclic species^572^ and furanoses^573^ along with the required glycosidic linkage.^574^ Significantly increasing the coverage of the FF was the inclusion of a variety of chemical groups along with testing on polysaccharides and glycan–protein interactions.^575,576^ They together represent the carbohydrate portion of the C36 FF that has been widely used for carbohydrates, glycolipids and glycoproteins.

A similar path was taken with the Drude polarizable FF. Extensive non-bonded parameter optimization was undertaken on acyclic polyalcohols,^577^ aldehydes, and ketones,^578^ as required for the treatment of linear alcohols. FF for furanose^579^ and pyranose^580^ monosaccharides were completed and subsequent adjustments were made to the LJ parameters of pyranoses to improve the treatment of stacking interactions that led to better diffusion behaviors of glucose.^581^ This was followed by parametrization of glycosidic linkages involving furanoses and pyranoses^582^ and extension to N-acetyl groups^583^ and both N- and O-linkages for glycoproteins.^584^ Application of both the C36 and Drude FF to mannose disaccharides showed good agreement with NMR observables.^585^ As with the rest of the Drude FF, the nomenclature, with few exceptions, has been designed to be identical to that of C36 for ready access.

### 10.6 Small Molecules

CGenFF and DGenFF have been developed to greatly broaden the coverage of FFs by rapidly generating topologies and parameters for a wide range of molecules including those of medicinal chemistry and ionic liquids. CGenFF initially leveraged the wide collection of topologies and parameters of C36. Its coverage then extended to drug-like molecules by applying an optimization protocol that maintains compatibility with C36. While the initial CGenFF paper focused on the general philosophy of the model and details of parameter optimization,^145^ the machinery for rapid application to small molecules was already in place. This included bond perception and atom typing algorithms along with the charge assignment protocol compatible with C36.^17,18^ In addition, the CGenFF program outputs penalties associated with charges and parameters not in the existing CGenFF parameter set, where the penalty is assessed based on the similarity between the algorithmically derived parameter and those available in the FF. As the penalty is not a direct measure of the “quality” of a given parameter, in many cases, parameters with relatively high penalties are often appropriate for modeling and simulation. CGenFF has been extended to include sulfonyl- and halogen-containing compounds.^586,587^ Treatment of halogens made use of lone pairs on aromatic Cl, Br and I atoms, allowing for modeling of halogen bonds involving weak favorable interactions with hydrogen bond acceptors along the C–X bond. Another major extension included parametrization of non-standard amino acids mentioned above,^526^ which were treated with CGenFF combined with the C36 protein FF.

The small-molecule DGenFF was designed to be analogous to CGenFF with some important differences. Notably, DGenFF takes advantage of the original CGenFF program in which the bond perception, atom typing, parameter assignment and bonded penalty assignment algorithms were based on a rule-based approach that creates a rules file specific for the DGenFF while using the same CGenFF program. Assigning electrostatic parameters for the Drude FF requires partial atomic charges, atomic polarizabilities, and Thole scaling factors, where a deep neural network (DNN) was developed for each term,^588^ building upon an earlier DNN model.^589^ Features were based on atom connectivity up to 1–5 bonded atoms along with local through-space atom type–atom type pairs with training targeting QM data on nearly 40,000 small model compounds (<200 Da). This approach rapidly assigns electrostatic parameters along with penalties based on populations of different atom types and their connectivity in the DNN training set. Additional validation against QM dipole moments and molecular polarizabilities on 900 FDA-approved compounds showed excellent agreement, indicating that the method is appropriate for drug-like molecules 200 to 700 Da in size. Note that including distance features in the DNN to model asymmetric electrostatic parameters on lone pairs requires that molecules have approximately correct 3D geometries as well as correct ionization and tautomer states.

Efforts are ongoing to extend the coverage of DGenFF comparable to that of CGenFF. To date, extension to halogens has been completed.^590^ It includes the presence of lone pairs to accurately treat halogen bonds along with careful optimization of the anisotropic atomic polarizability on the halogens Cl, Br, and I and inclusion of LJ parameters on Drude particles, as was done for water–Mg^2+^ interactions discussed above. These latter terms allow for accurate modeling of out-of-plane interactions with hydrogen bond donors where the halogen atom serves as a hydrogen bond acceptor, which is more favorable than halogen bonds and is present in a large number of ligand–protein complexes.^591^ Further extension of DGenFF will be facilitated by a DNN-based workflow to optimize LJ parameters of new atom types.^592^ Upon completing full coverage, global optimization of LJ parameters will be undertaken, as done for a subset of atom types in the Drude FF.^593^ The resulting FF parameters yield good agreement with both pure solvent properties and hydration free energies for a large collection of small molecules. To our knowledge, agreement with both classes of condensed phase properties has not been attained with any additive FF, even when a similar global optimization protocol was used.^594^

A final issue concerning FFs in general is the validity of implementation.^595^ CGenFF is based on a specific algorithm to assign atom types yielding model compound topologies used for parameter assignment and optimization. When other algorithms are used based on analogy, the resulting charges and parameters are inconsistent with those on which the FF was optimized. For correct implementation of CGenFF, individual molecules can be uploaded online by users from educational institutions at https://cgenff.silcsbio.com/ and the CGenFF program can be obtained at no charge for users from educational institutions from Silcsbio LLC (silcsbio.com).

## 11 Mixed Quantum Mechanics/Molecular Mechanics (QM/MM) Methods

### 11.1 Background

QM/MM methods are practical and efficient approaches for simulating chemical reactions in condensed phase including enzyme catalysis.^596−599^ A QM method is necessary for modeling changes in electronic structure such as bond formation and cleavage, photochemical reactions, and electron transfer in redox catalysis by metalloenzymes. However, it is neither practical nor necessary to treat an entire substrate–enzyme complex and the surrounding solvent at the QM level. This is further complicated by the need to sample multiple protein and solvent configurations to determine the free energy change along a reaction pathway. A combined QM/MM approach addresses this challenge by selectively applying the QM treatment to a region of the system involved in the reaction, such as the substrate, cofactors and key amino acid residues directly participating in the chemical event. This ‘QM subsystem’ is embedded in the rest of the system represented by an MM FF.^596,600,601^ Because of its effectiveness and simplicity, QM/MM methods have become the *de facto* choice for simulating enzyme reactions.^597−599,602,603^

A QM/MM approach was first implemented in CHARMM by Field and Bash in 1987,^601,604^ employing the SE ‘neglect of diatomic differential overlap’ (NDDO) method along with the CHARMM FF. QM/MM approaches have since continuously embraced diverse methods, including *ab initio* (AI), DFT, and SE QM alternatives. The SE-QM methods encompass both the NDDO-based models^161,605−608^ and the density functional tight binding (DFTB) methods, also referred to as the self-consistent-charge DFTB (SCC-DFTB) methods.^609^ Both of them are incorporated into CHARMM.

The QUANTUM module was the first SE-QM/MM method implemented in CHARMM, which was based on the MOPAC program (version 4.0).^610^ Subsequently, two new NDDO-based SQUANTM^3^ and MNDO97^147,611^ modules were added. The SQUANTM module was based on an implementation in the AMBER program,^612^ and the MNDO97 module was derived from a stand-alone MNDO97 program.^613^ The latter has recently been rewritten for computational speed and parallelization.^147^ The DFTB method was similarly implemented in CHARMM.^163^ Due to their computational efficiency, these SE-QM/MM methods are frequently used in conjunction with other free energy simulation techniques, including US,^138^ SM,^139,614^ reaction path,^148,615^ and FEP.^604^

For AI and DFT-based QM/MM methods, CHARMM provides robust interfaces to external softwares including Q-Chem,^616^ GAMESS-US,^617,618^ GAMESS-UK,^619^ CADPAC,^620^ and Gaussian16.^621^ In addition, the MSCALE module provides a flexible means for accessing other QM programs, such as MOLPRO.^622^ Unlike the SE-QM/MM modules of CHARMM, other packages for AI and DFT calculations must be obtained separately. Except for GAMESS-US and GAMESS-UK that can be compiled as a single executable within CHARMM, other packages should be installed separately.

### 11.2 QM/MM Potentials and Practical Considerations

The effective QM/MM Hamiltonian operator is
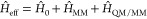
51where  and  describe the QM and MM subsystems, respectively, and  describes the interaction between the two. The latter is further decomposed into electrostatic, van der Waals, and the QM–MM boundary terms:
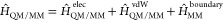
52In CHARMM,  is solved self-consistently with , while  is modeled with a LJ potential. The total energy of the system is
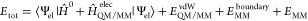
53where Ψ_el_ represents the Hartree–Fock wave function for electrons and nuclei of the QM region. For evaluation. the first three terms in Eq. 53 are determined in the QM/MM module while *E*_MM_ uses MM energy routines. For performing MD simulation or energy minimization, the QM electron density matrix from the previous MD/minimization step can be used as the initial guess for the next SCF calculation in Eq. 53, with optional addition of small random perturbations to reduce hysteresis and accelerate the convergence of the QM and QM/MM energies. This is implemented in all SE-QM/MM modules as well as in AI-QM/MM modules supporting GAMESS-US, GAMESS-UK and Q-Chem.

In Eq. 53, *E*_MM_^boundary^ addresses cases where the QM and MM division occurs across covalent bonds leaving the QM region with unsaturated dangling bonds. This commonly occurs in enzyme simulations where specific side chains are included in the QM region, for which three methods are available in CHARMM. The first is the hydrogen link (H-link) atom approach, where a hydrogen atom is added within the QM region to saturate and cap the dangling bond.^601,623,624^ The H-link method is conceptually simple, so it has been widely adopted in various packages. In CHARMM, users can introduce an MM angle term to keep the H-link atom aligned with the replaced QM–MM bond. Also, charges on nearby MM atoms can be reassigned to minimize artificial polarization around the QM–MM boundary. The second model is the double-link atom method (DLAM) where an additional H-link atom is introduced at the MM atom site of the QM–MM covalent bond to achieve bond saturation at both of the loose QM and MM ends^625^ (Section 11.6). This method can be used together with delocalized Gaussian MM (DGMM) charges to mimic the delocalization of charge densities on MM atoms.^626^ The third is the generalized hybrid orbital (GHO) method.^627−629^ While the first two methods introduce additional degrees of freedom via the link atoms and alter local electrostatic potential by adjusting partial charges, the GHO method treats the QM boundary atom as a special *sp*3-hybridized carbon and also as an MM atom connected to nearby MM atoms. Its three *sp*3-hybrid orbitals pointing toward the connected MM atoms called auxiliary orbitals, are fixed with their electron densities assigned based on their MM charges. The remaining *sp*3-hybrid orbital called the active orbital, is optimized during the SCF iteration. In addition, MM FFs are applied to the GHO atoms to maintain the surrounding geometry.

The above boundary methods, particularly the GHO method, are specifically designed for covalent bonds between two *sp*3-hybridized carbon atoms, and they are not recommended for arbitrary covalent boundaries. This ensures that the covalent boundary does not perturb the geometry around the QM–MM bond and minimizes artificial polarization of the QM electron density. The GHO method implemented in CHARMM supports all four SE-QM/MM modules (QUANTUM, SQUANTM, MNDO97 and SCC-DFTB) and AI/DFT QM/MM methods through the GAMESS-US interface. The H-link atom approach is available for all QM/MM modules of CHARMM.

In QM/MM simulations, one must decide on: (1) the QM model, (2) atoms for the QM region, and (3) representation for the QM/MM covalent boundary, and (4) the boundary condition of the whole system.^630,631^ For (1), among AI-QM, DFT and SE-QM, computational errors contributing to the final results should be considered. While AI-QM and DFT methods are more accurate, their high computational cost limits routine use in extensive MD and free energy simulations. Thus, selection of the QM theory level and the basis set should be tailored to individual problems. Also, the high accuracy of computationally expensive QM methods such as a coupled-cluster model may be overshadowed by the statistical noise itself. In such cases, an SE-QM method would be more suitable for lengthy QM/MM simulations. However, they require calibration against AI-QM/DFT levels for the reaction under consideration.^608,632−634^ Among the three NDDO-based SE modules, QUANTUM and MNDO97 in CHARMM now have the option to read non-standard parameters without modifying the source code, obviating the need to rebuild the executable for reaction-specific parametrization.

About the choice of the QM region, there are recent debates about the minimum QM region size required for convergence.^377,635−640^ It affects the computational cost and the extent of sampling needed. Advances in efficient algorithms and specialized hardware enable systematic exploration of the QM size for the desired accuracy. However, such an investigation is currently feasible only for relatively small systems, and chemical intuition still remains crucial for selecting the QM region. In any event, we note that QM/MM methods are fundamentally empirical approximations. There is no reason to expect that an arbitrary combination of QM and MM models will reproduce the full quantum results. One should carefully optimize parameters for separating a full QM system into two distinct QM and MM models, to determine the minimum size of the QM region.

CHARMM can perform the QM/MM calculations using both PBC and the solvent boundary condition.^630^ When using PBC, the QM/MM-Ewald^641^ and QM/MM-PME methods^612,642^ can be used. In this case, the *E*_MM_ term in Eq. 53 includes MM–MM interactions with all images as for the regular PME and the  term includes long-range electrostatic interactions of all MM and QM periodic images with the QM charges. The QM/MM-Ewald method is available in all SE-QM/MM methods, while the QM/MM-PME method is available in the SQUANTM and MNDO97 QM modules; the QM/MM-Ewald method is also supported by the AI/DFT-QM/MM method employing the QChem package.^643^ For the DFTB method, the GSBP method is also available as an alternative way to incorporate long-range electrostatic interactions into the QM/MM framework.^644,645^ Otherwise, it is recommended not to use any cutoff scheme for non-bonded interactions for balanced interactions between the QM–MM and MM–MM pairs.^641^ This is because in Eq. 53, any MM atom included in the QM/MM interactions, e.g., those within the cutoff distance of any QM atom, interacts with all QM atoms and thus directly polarizes the QM electron density.

### 11.3 Recent Advances in SE-QM/MM Methods

Main strengths of SE-QM/MM methods over AI/DFT-QM/MM methods are their efficiency and flexible functional forms that allow recalibration against target data.^608,633,634^ Extended MD simulations within reasonable computational time is thus possible while yielding accuracy tailored to individual systems. In enzyme mechanism studies, SE-QM/MM methods are frequently employed together with the RXNCOR module for US simulations and more recently, with the SM.^139,646,647^ They can also be used in FEP simulations such as calculating solvation free energies of solutes and ligand p*K*_a_ values, functionalities available in the QUANTUM, SQUANTM, and SCC-DFTB modules.^598,645,648,649^

**Figure 14:**
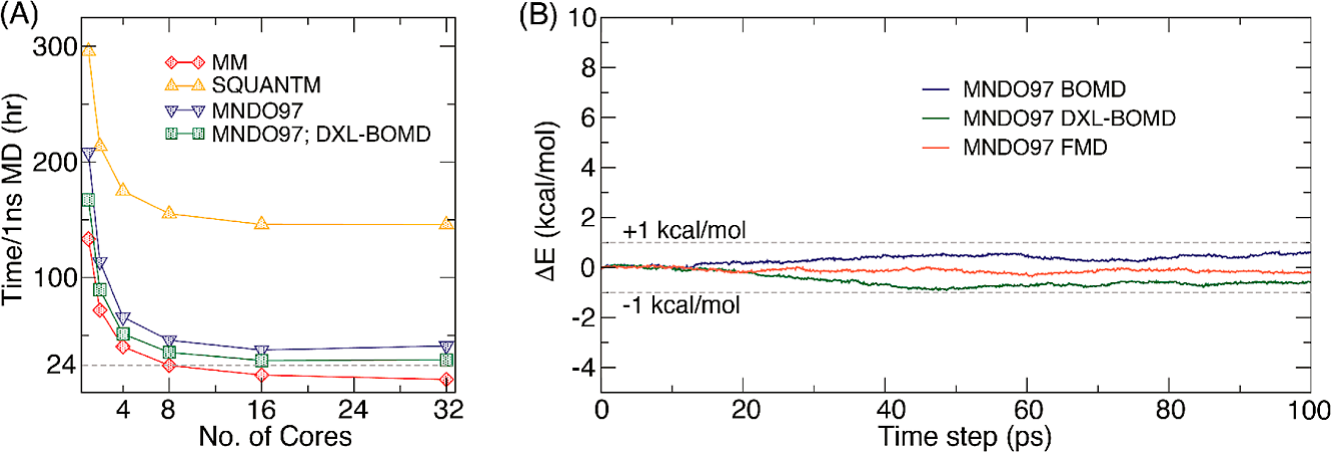
Performance of QM/MM methods.^147^ (A) Wall time in hours per 1-ns MD simulation for insulin receptor kinase versus the number of CPU cores, for the MM-only, SQUANTM, MNDO97 BOMD, and MNDO97 with DXL-BOMD methods. (B) Energy conservation in different SCF accelerator implementations for adenylate kinase, showing less than 1 kcal/mol deviation of the total energy during 100-ps NVE MD simulations. Simulation systems consist of (A) 76 QM atoms and 28,823 MM atoms and (B) 92 QM atoms and 47,201 MM atoms. In both cases, the QM region is treated with the AM1/d-PhoT SE QM model^608^ for the MNDO97 module and with the AM1 model for the SQUANTM module.

CHARMM also has several acceleration algorithms for the SE-QM/MM methods, including the direct inversion of the iterative subspace (DIIS) extrapolation scheme for faster SCF convergence^650,651^ and the pseudodiagonalization algorithm for the Fock matrix. In addition, the MNDO97 module has recently incorporated the MPI parallelization and several new SCF accelerators, achieving more than 10-fold speed up (Figure 14).^147,165^ The newly implemented SCF accelerators are as follows:

#### 11.3.1 Extended Lagrangian MD (ELMD).^165^

This method^652^ performs MD simulations with the electron density of the QM subsystem propagated by the Lagrangian:
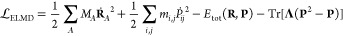
54Here, **R** denotes the QM and MM coordinate with mass *M*_*A*_ (*A*: atom index). **P** is the electron density matrix with mass *m*_*ij*_ for the corresponding element.  and  are time derivatives. The Lagrange multiplier **Λ** enforces the idempotency constraint on **P**. Alternatively, **P** can be propagated using the curvy-steps unitary update algorithm.^653^ Both methods avoid time-consuming SCF iteration at the expense of a smaller integration time step for the density matrix propagation. This limitation can be alleviated by applying the multiple time step (MTS) approach where nuclear coordinates are propagated with a larger time step, typically 0.5 or 1 fs, as commonly used in QM/MM MD simulations.

#### 11.3.2 Extended Lagrangian Born–Oppenheimer MD with Dissipation (DXL-BOMD).^147^

The idempotency condition of Eq. 54 is modified into a harmonic restraint for the auxiliary density variable **D**, to oscillate around the converged SCF density:
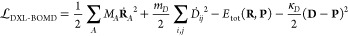
55The true (SCF) density **P** is approximated by **D** that serves as the initial guess in the SCF iteration, and κ_*D*_ is the force constant. **D** is extrapolated based on a predetermined number of previous SCF densities.^654,655^ The SCF iteration is then performed for a given number of SCF steps. In practice, Eq. 55 is solved in the limit of vanishing *m*_*D*_, resulting in coupled equations of motion, one for the nuclear position and the other for **D**, thereby eliminating the dependence of results on *m*_*D*_. This method is also supported by the DFTB QM/MM module.^655^

#### 11.3.3 Fock Matrix Dynamics (FMD).^147^

The Fock matrix is directly extrapolated based on its elements determined from previous MD steps, followed by regular SCF iteration until convergence.^656,657^ Both DXL-BOMD and FMD methods significantly reduce the number of SCF iterations compared to conventional (BOMD) SCF calculations while maintaining energy conservation.^147^

**Figure 15:**
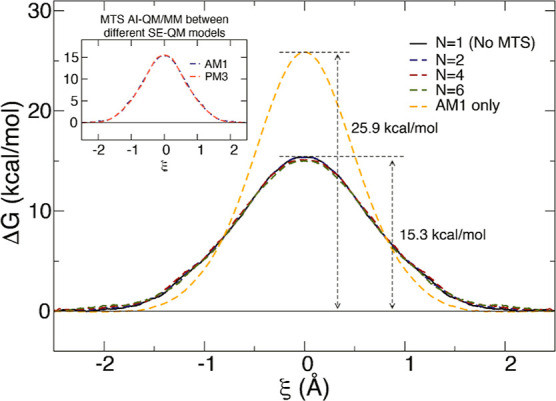
PMF for the S_*N*_2 reaction between CH_3_Cl and Cl^–^ in water.^642^ Results from MTS simulations with varying number of MD steps *N*, for evaluating the AI-QM/MM correction term. The MTS AI-QM/MM simulations were carried out using the AM1 and HF/3-21G methods for the low- and high-level QM theories, respectively. “AM1 only” results are from the AM1 SE-QM/MM simulations. The inset compares the impact of low-level QM theory on the PMF, while the high-level theory remains at the HF/3-21G level.

In addition to efficiency, SE-QM/MM methods in CHARMM are being developed to improve accuracy. Recognizing the importance of non-bonded interactions, the MNDO97 and DFTB modules have implemented Grimme’s dispersion and hydrogen bond correction terms.^638,658,659^ To further improve the quality of the PES, a simple valence bond-like (SVB) term has been introduced in the NDDO-based SE-QM/MM modules.^646,660^ In addition, the SQUANTM module integrates the SE-QM/MM and GAMESS-UK AI/DFT-QM/MM methods, introducing a dual-level approach that interpolates the QM/MM energy to the AI/DFT-QM/MM level of theory.^642^ This method is compatible with the MTS algorithm so that MD simulations are performed at the SE-QM/MM level while simultaneously correcting energies and gradients at the target AI/DFT-QM/MM level over a longer time step (Figure 15). These developments enable simulations of highly challenging systems with unprecedented accuracy and efficiency, pushing the boundaries of QM/MM methods.

### 11.4 Path-Integral-Free Energy Perturbation for Nuclear Quantum Effects

An important approach for treating nuclear quantum effects (NQE) is the Feynman path integral (PI) formalism^661^ that describes the wave function by an ensemble of paths weighed by the classical action for each, to capture Schrödinger’s delocalized wave behavior. It is readily generalizable to multiparticle systems, and it naturally accounts for thermal effects and sampling can be performed in MC and MD simulations.

The quantum transition state theory (QTST) rate constant may be computed with PI (PI-QTST).^662^ Consider a system composed of a set of QM atoms with coordinates **r** embedded in a bath of classical atoms with coordinates **R** in thermal equilibrium. The QM partition function *Q*_*Q*_ can be written as the trace of the thermal density matrix ρ
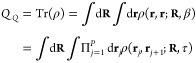
56where *P* is the number of quasi-particles or beads and τ = β/*P* (β = 1/*k*_*B*_*T*). For a closed ring-polymer chain, **r**_1_ = **r**_*P*+1_. In the high-*T* limit (τ → 0 and *P* → *∞*), the semiclassical primitive approximation^663^ can be used for ρ
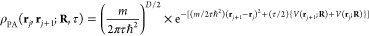
57where *V* is the system potential (i.e., a QM/MM potential), *m* is the quantum particle mass, and *D* is the dimension of **r**. The description above is isomorphic to a classical ring of beads system connected via harmonic springs, and forms the basis for the implementation in CHARMM where the bead distribution is sampled using MC simulations.

The rate constant of PI-QTST^662^ is defined as
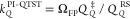
58where Ω_FP_ is a free-particle (FP) prefactor, and *Q*_*Q*_^‡^ and *Q*_*Q*_^RS^ are the quantum partition functions for the transition and reactant states, respectively. Although one can compute the PI-QTST directly, when using expensive potentials like QM/MM, it is convenient to compute the correction to the classical TST due to NQE
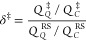
59where the subscript *C* denotes the corresponding classical partition functions. The above includes both quantum vibration (zero-point energy) and tunneling effects. To calculate the quantum to classical ratio of the partition function, a double average scheme^664,665^ can be used
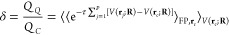
60where **r**_*c*_ is the centroid coordinate. The outer (classical) average is obtained using standard simulation techniques, while the delocalized QM description comes from the inner FP average. This approach is practical since the classical and quantum simulations are performed separately. In CHARMM, the NQE atoms are defined as QM atoms. Currently, the PI method works with all SE-QM/MM modules in CHARMM as well as with AI/DFT-QM/MM using CHARMM and Q-Chem.

**Figure 16:**
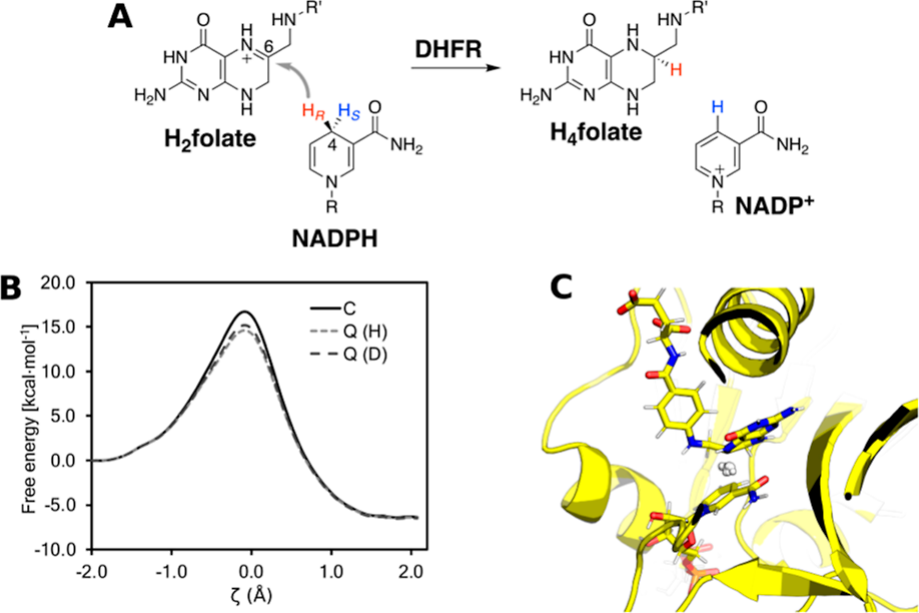
Hydride transfer reaction in dihydrofolate reductase.^633^ (A) Reaction mechanism. (B) Classic (solid line) and quantum (dashed lines) PMFs for hydride and deuteride transfer. (C) Active site for the hydride transfer from NADPH to H_2_ folate. The transferring hydride is described using PI with 32 beads.

A well-known challenge with PI simulations is the difficulty with sampling the polymer ring due to the harmonic coupling between the beads (Eq. 57).^663^ In CHARMM, the inner average in Eq. 60 for the FP PI sampling is performed using the bisection algorithm^666,667^ extended to a ring of quasi-particles^668,669^ or using the staging algorithm.^670,671^ Since each new configuration in the bisection or staging PI sampling is independent of the distribution of beads in previous configurations, the PI rapidly converges, which is essential for accurate calculation of the NQE and absolute rate constants.^633,672−674^ However, specialized techniques are required to precisely compute isotope effects due to minute differences in free energy. In CHARMM, a novel mass-perturbation technique termed PI-FEP, was developed to directly compute the free energy difference between isotopes.^675^ Since relative free energies between the distributions of heavy and light particles are determined by FEP (Figure 16), the precision of the computed kinetic isotope effect (KIE) is of experimental quality for both the primary and secondary KIEs.^671,676−683^

To reduce the computational cost of PI simulations in CHARMM, higher-order factorization of the density matrix operator has been adopted,^684−686^ which converges with a considerably smaller number of beads at the expense of computing the potential gradient in addition to the potential.^671^ This method can be combined with the PI-FEP approach to efficiently compute the KIE.^679^

Information about tunneling can be obtained by inspecting the particle momentum distribution computed using open-chain PI (i.e., **r**_1_ ≠ **r**_*P*+1_).^687−689^ Whereas closed-chain PI only samples diagonal elements of ρ, open-chain PI simulations also sample off-diagonal elements, and it can sample both the anisotropic and isotropic momentum distribution of a transferring hydrogen (H^+^/H^–^/H·) during a reaction.

### 11.5 Density Functional Tight Binding (DFTB) Module

One versatile QM/MM module in CHARMM is based on the DFTB approach,^692,693^ which is an approximation method that aims to strike the balance between computational efficiency and accuracy. Also referred to as SCC-DFTB,^609^ it is an SE QM method in the sense that it employs a minimal basis and the two-center approximation for electron integrals, which are key approximations for NDDO-based SE methods^694^ such as AM1, PM3 and OM2 (Section 11.1). On the other hand, most of the parameters in the DFTB approach are computed based on atomic or diatomic molecules, and the most empirical aspect of the parametrization concerns those used to derive the atomic/diatomic electronic properties and the pairwise repulsive potentials (e.g., confinement radius). The most popular approach for biomolecular applications is the DFTB3/3OB model,^164,695^ which has been parametrized for elements commonly encountered in organic and biomolecular systems: O, N, C, H, S, P, Na, K, Mg, Ca, Zn, Cu and the halogens. For recent reviews of the development and application of the DFTB3 method for condensed phase applications, see refs (658 and 696).

The DFTB3 model is integrated with MM model in CHARMM through the standard electrostatic embedding scheme^163^ where the DFTB3 atoms are represented as Mulliken charges; alternative DFTB3/MM electrostatic interaction models have also been implemented^697^ that consider the finite spatial distributions of the DFTB3 and MM charges. In terms of boundary conditions, the DFTB3/MM model can be used together with either a GSBP^644^ or the PBC with either Ewald summation^649^ or PME^641^ (Section 11.2). For localized reactions, the DFTB3/MM-GSBP approach is computationally most efficient and generally agrees with the more expensive DFTB3/MM-PME approach.^141^ For systems in which the chemical reaction is coupled with considerable conformational rearrangements, the PBC-based approach is more appropriate. Another technical detail relevant to many QM/MM applications is the flexible inner region ensemble separator (FIRES) potential^698^ available in CHARMM (Section 11.7), which prevents the exchange of QM and MM water molecules and thus particularly important for solution reactions^699^ or for solvent-accessible active sites.^700^

The DFTB3/MM approach can be used together with many key functionalities in CHARMM, especially various types of free energy simulations that are essential to quantitative analysis of chemical transformations. For chemical reactions, they include US with the RXNCOR module, an interface with PLUMED^701^ for various metadynamics simulations,^682,690,702−704^ and the SM available in the STRINGM module^139,297,705^ (Section 7.3). Another useful approach for improved sampling is replica exchange US through the REPDSTR module.^297^ For alchemical free energy simulations, DFTB3/MM works with the PERT module.^297,706^ For applications such as redox potential^310^ and p*K*_a_ calculations,^649,707^ the DFTB3/MM model also works with the BLOCK module.

**Figure 17:**
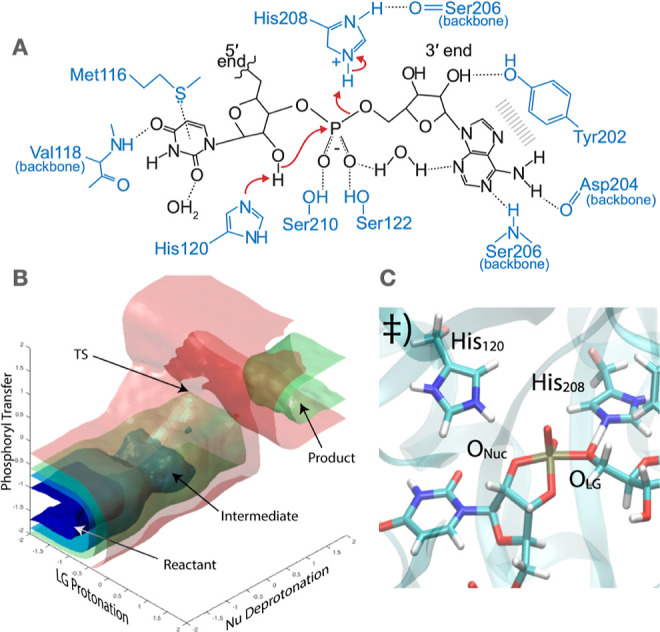
DFTB3/MM free energy simulations for the catalysis in Usb1.^690^ (A) The putative catalytic mechanism of Usb1. (B) The 3D free energy surface from DFTB3/MM multiwalker metadynamics simulations. The three CVs describe proton transfers associated with the catalytic acid/base and the phosphoryl transfer (red arrows in panel A). (C) The transition state structure from the DFTB3/MM free energy simulations features the transfer of a single proton between H208 and the leaving group. This was subsequently confirmed with proton inventory experiments.^690^ Panels A and C were adapted from ref (690), which was published by the Oxford University Press.

In the following, the DFTB3/MM method is illustrated with two types of free energy simulations. For chemical reaction, the catalysis in Usb1, an exoribonuclease that shortens the oligo-uridine tail of U6 snRNA,^690^ is used as an example. In the proposed catalytic mechanism, two active-site histidine residues serve as the catalytic base and acid, respectively (Figure 17A). It was studied with DFTB3/MM metadynamics simulations using three CVs that describe proton transfer involving the catalytic base (H120), the phosphoryl transfer reaction, and the proton transfer involving the catalytic acid (H208), respectively. Multiwalker metadynamics calculations were run with 300–500 walkers, each of which was sampled for 0.5–1 ns, leading to a cumulative sampling of 0.1–0.2 μs for constructing the 3D PMF (Figure 17B). This level of sampling for QM/MM simulations is possible only with SE-type QM methods such as DFTB3, highlighting the value of calibrated SE QM/MM simulations for complex biomolecular processes.^696,703^ The transition state structure captured in the DFTB3/MM simulations (Figure 17C) suggests that one proton is in flight, and the predicted feature was subsequently confirmed experimentally.^690^

**Figure 18:**
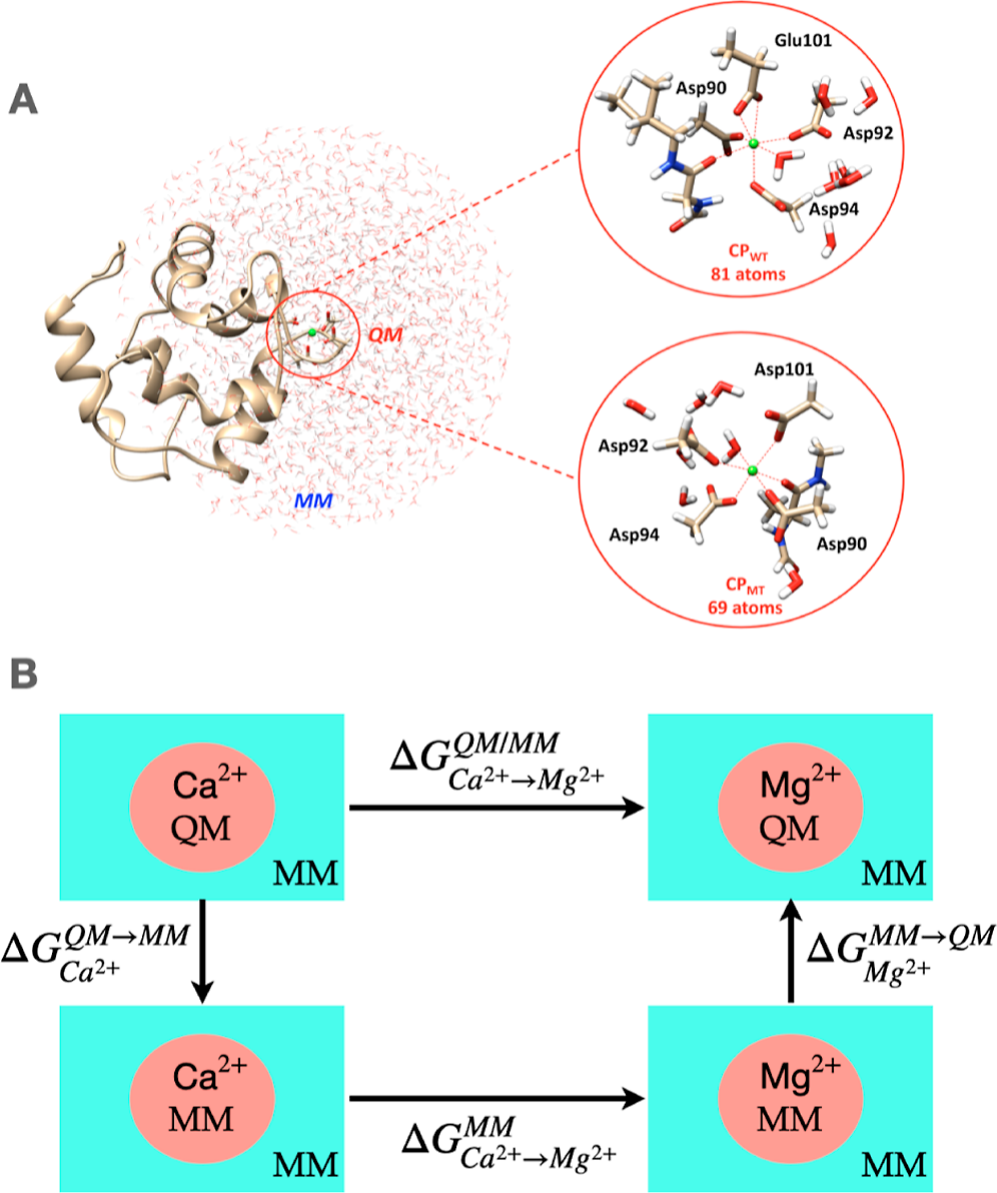
DFTB3/MM FEP simulations for the Ca^2+^/Mg^2+^ binding selectivity in the Ca^2+^ binding protein, carp parvalbumin (CP), and its mutant (D51A/E101D/F102W). (A) The DFTB3/MM-GSBP setup that illustrates the QM regions for the WT and mutant CP simulations. (B) Thermodynamic cycle used to probe the free energy change for the Ca^2+^/Mg^2+^ conversion in a given environment. In simulations, the horizontal transitions occur at the MM level, and in the vertical transitions, a metal binding site is converted between MM and QM treatments using the PERT module in CHARMM. Reproduced from ref (691). Copyright [2024] American Chemical Society.

For alchemical free energy simulations, the binding selectivity of Mg^2+^ and Ca^2+^ in the Ca^2+^ binding protein carp parvalbumin (CP) and its mutant (D51A/E101D/F102W)^691^ is considered (Figure 18A). Experimentally,^708^ the WT CP was measured to bind more strongly to Ca^2+^ by 5.6 kcal/mol; in the mutant, Ca^2+^ is still preferred over Mg^2+^, although the selectivity is reduced to 1.6 kcal/mol. With a standard FF, the relative binding free energy of Mg^2+^ and Ca^2+^ to a protein is readily computed using alchemical free energy simulations; the metal ions are interconverted twice: once in the binding pocket and once in solution, and their free energy difference is the relative binding affinity. With non-polarizable FFs, such calculations did not yield the correct trend and the smaller Mg^2+^ was predicted to bind more strongly.^691,709^ While the same set of alchemical free energy simulations can, in principle, be carried out at the DFTB3/MM level, we adopt an alternative thermodynamic cycle, which involves converting the description of the metal ion and its ligands between MM and DFTB3 levels^297^ (Figure 18B). This has the advantage that structural changes during the MM/DFTB3 conversion are expected to be small and therefore convergence of the free energy simulation is rapid, minimizing the required DFTB3/MM computations. Encouragingly, with DFTB3/MM simulations,^691^ the calculated ΔΔ*G*_bind_ was ∼6.2 kcal/mol, in good agreement with the experimental value. These results highlight the value of a QM description of the metal binding site and support the role of electronic polarization^709^ and charge transfer^710^ in metal binding to proteins. For the mutant, different binding site models led to considerable variations in the computed relative binding affinities. With a coordination number of seven for Ca^2+^, which was shown by DFTB3/MM metadynamics to be the dominant coordination number for the mutant, the calculated relative binding affinity was ∼4.2 kcal/mol, also in fair agreement with the experimental value.

### 11.6 Double Link Atom Method (DLAM)

Accurately modeling chemical reactions in condensed phases using QM/MM is challenging, especially when partitioning across a covalent bond. Introducing dummy or link atoms serves as a bridge across the severed bond, acting as both a connection between QM and MM interfaces and an electron density cap for partitions. Typically hydrogen, a link atom can resemble the electronic character or features lost during truncation and is attached to a host group (HG) via the host atom (HA). The link atom is subject to interface with the MM and QM HGs. In the standard single link atom (SLA) scheme,^601,623^ the link atom is added to the QM HA to compensate or neutralize the charge of the QM fragment and cap the QM Hamiltonian. However, the addition of the QM link atom introduces a number of challenges at the QM/MM interface, primarily the treatment of electrostatics.

Various approaches have been developed to address QM/MM boundary effects in the QM fragment by adjusting the magnitude of polarization from the MM fragment, including the excluded group (EXGR) scheme,^623^ the charge shift scheme (CHSH),^711,712^ the divided frontier charge (DIV) scheme,^624^ and the distributed Gaussian (DG) method.^713,714^ For example, the SLA scheme treats MM atoms as point charges and excludes the MM HA from the QM/MM electrostatics, leaving an artificial partial charge at the interface on the MM HG. The EXGR scheme corrects for the added unrealistic partial charge at the interface by excluding all partial charges of the MM HG, whereas the DIV scheme corrects for the unrealistic charge at the interface by redistributing the excluded MM HA partial charge to the MM HG, and the CHSH scheme introduces a dipole to counterbalance for the charge shift. Alternatively, the DG method includes all electrostatic interactions of the MM HG; however, the MM partial charges are represented as Gaussian charge distributions and utilize a smoothing potential or blur width (σ) to smear the MM electron density where optimal σ values vary depending on the physical property of interest.^626,714^ Although these SLA-based schemes provide a balance for the QM fragment and interfacial electrostatics, they neglect the MM fragment which results in unbalanced forces and unrealistic electrostatics.

DLAM^626^ addresses the above issues by the addition of a link atom to cap the MM fragment but it has been infrequently used due to its challenging implementation. It can now be called directly in CHARMM to add link atoms to both the QM and MM host fragments. The DLAMadd command adds a QM link atom “QQ” to the QM HA, similar to the single link atom command (ADDLink), and adds an MM link atom “QM” to the MM HA. By default, the link atoms are placed 1.0 Å colinearly from the respective HA. The MM link, typically an MM hydrogen, bears a small partial charge that should preserve both the net charge and dipole of the MM HG. Balance can be achieved by shifting charge between the MM link and MM HA, but can be less straightforward in some systems. DLAM is used with the DG method and employs the same σ value for all MM atoms. As σ and MM link partial charge are free parameters in DLAM that can be tuned to balance electrostatics, identifying reliable parameters for complex systems can be a challenge. Currently, optimizations of σ and MM link partial charges for amino acids compatible with the CHARMM and Amber FFs are underway for ease of use.

### 11.7 Flexible Inner Region Ensemble Separator (FIRES)

A QM/MM methodology in which a solute and the nearest water molecules are represented at a high *ab initio* level, offers a powerful strategy to study the hydration structure around small ions in the aqueous phase. However, one challenge with solvent molecules in hybrid QM/MM simulation is that they are free to diffuse away from the region of interest, and be replaced by MM solvent molecules that provide presumlably a less accurate model. To resolve this issue, FIRES was designed in which the ion and a fixed number of nearest water molecules form a dynamical and flexible inner region that is represented with a high level *ab initio* QM method, while the water molecules in the surrounding bulk form an outer region that is represented by a classical FF. Simulations with FIRES yield rigorously correct thermodynamic averages as long as the solvent molecules in the flexible inner and outer regions are not allowed to exchange. The method was used to study hydration structure around Na^+^ and K^+^,^698^ and Mg^2+^ and Zn^2+^.^715^ To obtain a more efficient dynamical propagation algorithm, it is necessary to manage the computational cost of the QM part. To this end, a MTS dual-Hamiltonian propagation algorithm was designed by which the trajectory is propagated at every time step via a computationally inexpensive QM Hamiltonian, and then corrected less frequently using a more accurate and computationally expensive QM Hamiltonian.^716^

### 11.8 Combining QM/MM with Gaussian Process Machine Learning Potentials

**Figure 19:**
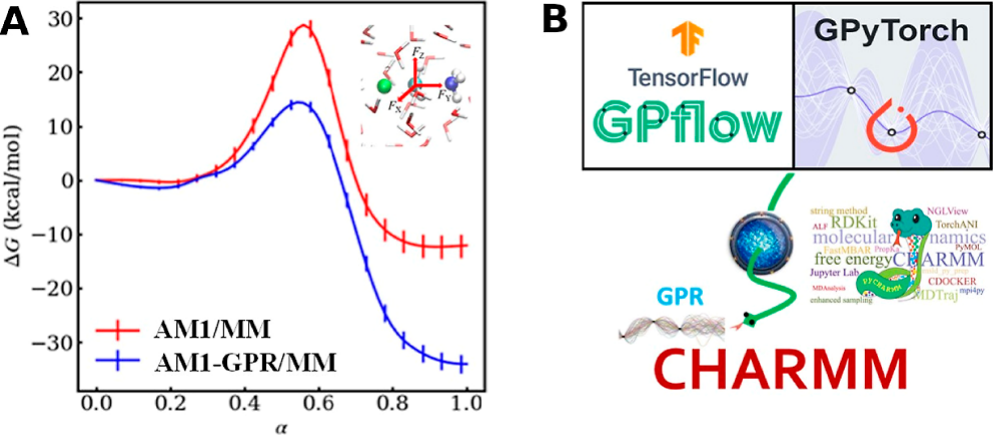
(A) PMFs of the Menshutkin reaction (NH_3_ + CH_3_Cl → NH_3_CH_3_^+^ + Cl^–^) simulated at the AM1/MM and AM1-GPR/MM levels.^717^ (B) The scheme of combining GPR Python libraries and CHARMM through pyCHARMM.

The pyCHARMM^9^ interface in CHARMM has facilitated advanced uses of QM/MM potentials in conjunction with Python-based ML potentials, including those described by neural networks^54^ and Gaussian process regression (GPR).^717,718^ Built upon multivariate Gaussian distribution of latent functions, GPR is a non-parametric, kernel-based stochastic inference ML approach that maximizes the likelihood of training data observation.^719^ In simulations, GPR has been employed to model the relationship between molecular descriptors and the PES (reviewed in ref (363)). Recently, GPR has been utilized to develop delta-ML potentials to improve SE-QM/MM free energy simulations.^717,718^ By combining the AM1/MM potential in CHARMM and energy-based streaming sparse GPR (SSGPR) models, AI-QM/MM quality PES information can be learned along the string free energy paths in a data-efficient manner.^718^ Using the extended-kernel GPR with derivative observations (GPRwDO), both energy and force matching can be employed to improve SE-QM/MM free energy simulations.^717^Figure 19A shows the PMFs for the Menshutkin reaction simulated at the AM1/MM level, before and after deploying the GPR correction model where the latter significantly alleviates overestimation of the free energy barrier and the product free energy. In these QM-GPR/MM studies, GPR models trained using Python libraries such as GPflow^720^ and GPyTorch^721^ are deployed on the fly during MD simulations (Figure 19B). A Colab-based tutorial is available to demonstrate the use of the related Python libraries to train basic ML models for reactive systems.^722^ A similar tutorial for training GPR models for QM/MM systems using CHARMM and pyCHARMM is currently under development.

### 11.9 Multistate Empirical Valence Bond (MS-EVB)

The MS-EVB module of CHARMM^723,724^ is an efficient method for representing reactive PES, e.g., in enzymes where a system moves from a reactant to a product topology (see ref (725) for implementation details). The most common approach to EVB involves a pseudo-Hamiltonian matrix **H**(**q**) constructed from two diabatic reactant (*R*) and product (*P*) basis functions, yielding a 2 × 2 matrix
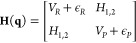
61where *V*_*R*_ and *V*_*P*_ are the potential energies of the reactant and product diabatic states at a given geometry **q**, obtained from standard FF. ϵ_*R*_ and ϵ_*P*_ are constant diagonal energy shifts usually chosen to reproduce the known exo- or endothermicity of the reaction. The off-diagonal element *H*_1,2_ couples the reactant and product basis functions, which is usually a simple function of atomic coordinates. In CHARMM, one can choose constants or 1D/2D Gaussians, which are functions of one or two distances between atoms. **H** can be diagonalized into **D** = **U**^*T*^**HU**, where the diagonal matrix **D** contains the eigenvalues and **U** consists of eigenvectors of **H**. Applying the Hellman-Feynman relation gives a matrix of Cartesian atomic forces
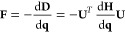
62which contains the gradient vector **F**_*i*_ for each adiabatic state corresponding to the *i*-th eigenvalue of **D** in increasing order of energy. **F**_0_ contains forces corresponding to the lowest eigenvalue λ_0_, and is used for dynamics propagation on the adiabatic ground state.

The CHARMM-EVB implementation utilizes MPI to parallelize the energy and force calculation for each topological replica at any given time step, achieving near-linear scaling with the number of topological replicas so that the number of topological replicas is limited only by the number of MPI threads running on the given hardware.

### 11.10 Reactive MD

Following chemical reactions in time and space is a central aspect of chemistry. For computer-based methods, *ab initio* MD methods at correlated levels are usually too prohibitive, in particular if statistically significant numbers of trajectories need to be run. Earlier and previous empirical efforts to describe bond breaking and formation include approaches based on bond order and bond strength.^726−729^ Alternatively, chemical reactivity can be modeled as a linear combination of empirical energy functions describing two or multiple atom connectivities (reactant and one or several products) and to mix these representations. This leads to reactive PESs as in multistate adiabatic reactive MD (MS-ARMD).^730−732^ Here, the PESs are mixed according to
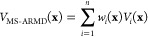
63The weights *w*_*i*_(**x**) are obtained by normalizing the Boltzmann distributed raw weights *w*_*i*,0_(**x**)
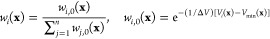
64where *V*_min_(**x**) is the minimal energy for a given configuration **x** and Δ*V* is a characteristic energy scale (switching parameter). Per construction (*cf.,*Eq. 64), only surfaces within a few times of Δ*V* from *V*_min_(**x**) will contribute to instantaneous configuration **x**. ARMD mixes different PESs *V*_*i*_ by using Gaussian and polynomial functions around the crossing points between states by fitting to reference data such as the MEP.^732^ Because the mixed PES *V*_MS-ARMD_(**x**) depends on energies of different states through weights *w*_*i*_ which in turn are analytical functions of the coordinates **x**, energy-conserving MS simulations can be run using MS-ARMD.^732^

A more recent extension combines MS-ARMD^733^ with VALBOND, a FF that allows to describe the geometries and dynamics of metal complexes.^734−736^ The formulation is reminiscent of empirical valence bond theory^600^ where diagonal terms are VALBOND descriptions of the states involved and off-diagonal terms describe the orbital overlap. MS-ARMD can also be combined with MM with proton transfer (MMPT),^737^ to follow proton transfer in gas and condensed phases.^738−741^

In the gas phase, MS-ARMD was used to study reactions such as hydrogen transfer in the photodissociation of H_2_SO_4_ → H_2_O + SO_3_^742^ and other atmospherically relevant molecules by following vibrational excitation of the OH stretch,^743,744^ the Claisen rearrangement reaction,^745^ or to investigate Diels–Alder reactions.^746^ Such studies provide insights into reaction mechanisms and relevant coordinates driving the process. As an example, for the Diels–Alder reaction between 2,3-dibromo-1,3-butadiene and maleic anhydride MS-ARMD emphasized the importance of rotations of the two reactants to reach the transition state.^746^

**Figure 20:**
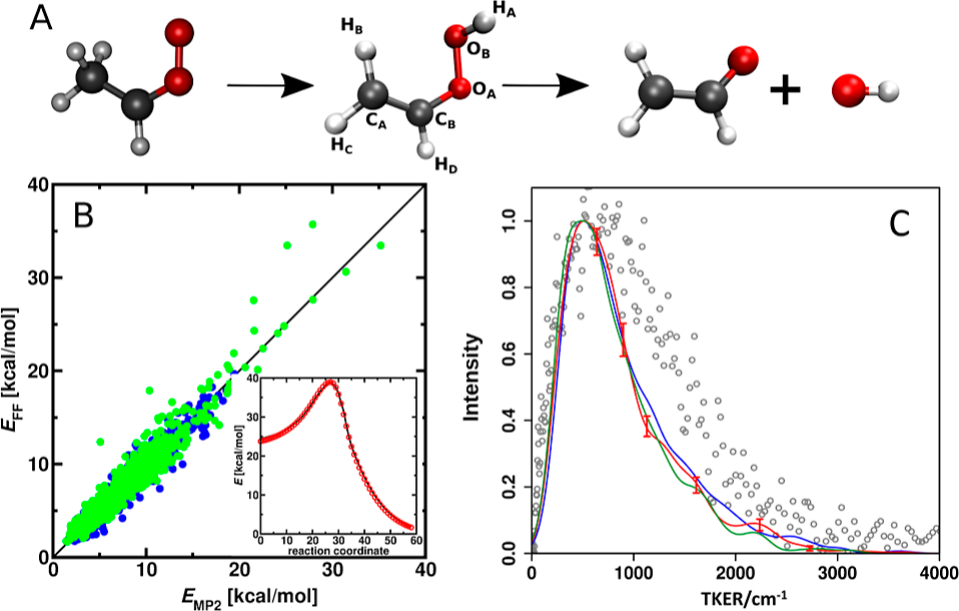
(A) OH-elimination following vibrational excitation of *syn-*CH_3_CHOO. (B) Performance of MS-ARMD. The RMSEs between reference *ab initio* energies and the fitted FF for reactant (blue) and product (green) are 1.1 and 1.2 kcal/mol, respectively. Inset: MEP calculated with MS-ARMD (red circles) compared with reference calculations (black line). (C) Distribution of the total kinetic energy release from several thousand trajectories following CH-excitation with ∼2 quanta using the MS-ARMD PES with OO scission energies of 22, 25, and 27 kcal/mol (blue, red, green). Open symbols are experimental results.^387^ Panels A and B reproduced from ref (382). Copyright [2021] American Chemical Society.

More recently, the unimolecular dissociation of vibrationally excited *syn-*CH_3_CHOO to form OH and CH_2_CHO was investigated (Figure 20).^382,389^ For the reactant and product states, MS-ARMD performs close to the chemical accuracy (∼1 kcal/mol) whereas the MEP is described considerably more accurately (inset in Figure 20B). Atomistic simulations using the MS-ARMD PES are about 2 orders of magnitude more efficient than using a neural network-based PES and about 6 orders of magnitude faster than *ab initio* MD simulations at the MP2 level of theory at which the MS-ARMD PES was developed. In other words, MS-ARMD simulations can be run routinely with high quality and in statistically significant numbers, as exemplified in Figure 20C.

Finally, biological systems were also studied using a combination of RKHS-based PESs and empirical FFs,^747,748^ which allowed structural interpretation of metastable states in MbNO and a molecularly refined understanding of ligand exchange (NO vs O_2_) at the heme-iron in truncated hemoglobin.

### 11.11 Indirect QM/MM Free Energy Simulations

The alchemical free energy functionality, specifically the PERT module of CHARMM, was described in detail in the 2009 paper.^3^ In addition to discrete intermediate states as a function of the coupling parameter λ, PERT supports slow-growth TI (SGTI) that changes λ incrementally at each step of the MD simulation. SGTI suffers from the Hamiltonian lag problem,^749^ hence it is rarely used directly. A free energy difference obtained from SGTI should be treated as non-equilibrium work (NEW),^750^ and the equilibrium free energy difference can be obtained by applying the Jarzynski equality^751^ or Crooks theorem^752^ to a sufficient number of SGTI runs.^753^ Such calculations can be automated by CHARMM’s scripting language, as illustrated below.

PERT fully supports CHARMM’s multiscale capabilities (MSCALE module).^262^ This makes it possible to compute free energy differences between two descriptions of a system, such as an MM description on one hand and a hybrid QM/MM description on the other hand. Most standard applications of alchemical free energy simulations, such as the calculation of relative binding free energies, employ equilibrium methods (TI,^329^ Bennett’s acceptance ratio method, BAR,^754^ or its multistate extension MBAR^12^). NEW based methods are also used.^169,755^ For such traditional applications, it is unclear whether equilibrium or non-equilibrium techniques are more efficient. The situation is different when one has to compute free energy differences between levels of theory, as is the case for the so-called indirect cycle QM/MM alchemical free energy simulations.

**Figure 21:**
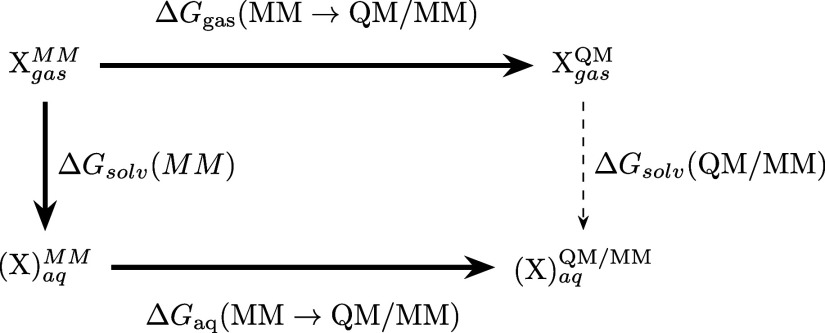
Illustration of an indirect cycle to compute a free energy difference at the QM/MM level of theory.

The calculation of free energies when using a QM/MM description poses two challenges: (1) It is slow, making it difficult to achieve sufficient sampling; and (2) standard recipes to realize alchemical transformations, such as soft-core potentials do not work with QM/MM Hamiltonians.^297,377,756^ Indirect cycles can circumvent both issues. The basic idea is illustrated using the calculation of an absolute solvation free energy (Figure 21). Instead of computing Δ*G*_solv_(QM/MM) directly, one computes Δ*G*_solv_(MM) where soft-core potentials, *etc.*, can be used without restrictions. In addition, free energy differences Δ*G*_α_(MM → QM/MM) (α: gas or aq) both in the gas and aqueous can be calculated, which yields
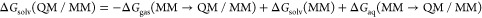
65

The calculation of Δ*G*_α_(MM → QM/MM) is challenging,^757^ but NEW-based approaches have been shown to be reliable and efficient.^758−761^

The combination of PERT and MSCALE enables the calculation of NEW values for transitioning from, e.g., a MM to a QM/MM description using solely CHARMM’s scripting language. By inserting as few as two hundred of such work values obtained from trivially parallel simulations, into the Jarzynski equality, Δ*G*_α_(MM → QM/MM) can be calculated accurately and efficiently in most cases.^760,761^ The NEW switches require equilibrium configurations sampled in the canonical ensemble, i.e., at the low level of theory. Restart files are saved at regular intervals, from which 2–5-ps long independent NEW switching simulations start in parallel.

A self-contained example illustrating this procedure is available at Zenodo.^762^ While the example uses the SCC-DFTB method as the high level theory,^163^ changes required for, e.g., a true DFT method are trivial. The key step is using MSCALE to employ one master (control) job and two slave jobs. The latter are responsible for computing energies and forces at the respective MM and QM/MM levels. The master process is primarily responsible for mixing forces/interactions and integrating the equations of motion. Care is needed to avoid double-counting if additional restraints or similar terms are used. The switching itself is realized as a SGTI calculation of the PERT module. By default, one switches linearly from λ = 0 to λ = 1 in 1,000–5,000 MD steps. The final result of each switch is the NEW value *W*, which can be saved or extracted automatically within the CHARMM script. Full automation can be achieved by calling the relevant CHARMM jobs from e.g., the Unix shell, a Python script, *etc.*

If the convergence of results obtained by the Jarzynski equality^751^ is in doubt, one can also use the Crooks theorem.^752^ In this case, a QM/MM-level equilibrium simulation is needed, followed by switches in the QM/MM → MM direction. The latter are again trivially parallel. While the computational cost of generating the initial configurations from the equilibrium QM/MM simulation is high, this workflow is still significantly more efficient than equilibrium-based approaches, which would require adequate sampling at each intermediate state (typically ten or more).

## 12 Boundary Condition, System Preparation, and Trajectory Analysis

In addition to performing simulation itself, an ability to prepare the simulation system in a desired initial state, impose appropriate boundary conditions or constraints, and analyze simulation trajectories are essential for making scientific discoveries. CHARMM has an extensive set of tools available for this purpose. Presently, at least 25% of more than 1.17 M lines of the CHARMM source code belong to this category. Example applications of the recently developed methods described below are given in references therein. A vast array of other existing tools can be found in the documentation as well as the example ‘Testcase’ input scripts provided in the CHARMM package.

### 12.1 Simulation and Analysis of Membrane Proteins

By virtue of its considerable functional flexibility, CHARMM has been a tool of choice in many studies of ion channels and membrane proteins. A theory was developed and implemented to account for the membrane potential and its representation by a constant electric field in computer simulations.^763^ It was subsequently used in studies of the Kv1.2 potassium channel^764^ and the voltage sensing domain of the voltage-sensitive phosphatase from *Ciona intestinalis*.^765^ Ion permeation through various channels was characterized,^766−769^ and the fundamental principles governing ion selectivity were explored.^770,771^ Computational methods with empirical energy restraints were developed to exploit information from low-resolution experimental data in structural refinement of membrane proteins. A particular attention was given to electron paramagnetic resonance (EPR) accessibility data,^772−774^ and double electron–electron resonance (DEER) technique that reports distance distribution between spin labels.^775−777^ The EPR/DEER methodology is also supported by CHARMM-GUI for easy setup of restrained simulations.^95^ Using these methods, the structure of various ion channels and membrane transporters were refined on the basis of EPR experimental data.^772,773,778−780^ Energy restraints were also developed to exploit information from mutational cross-link data,^781^ which resolved ambiguities about the conformation of the resting state of the voltage sensing domain of potassium channels.^782,783^

### 12.2 Coordinate Unwrapping for Diffusion Constants

In CHARMM, fractional coordinates are used to unwrap trajectories, which yields the same diffusion constants as the more recent “toroidal view preserving” method.^784^ It also highlights the need to correct calculated diffusion constants for PBC artifacts, especially those in lipid bilayers. The translational diffusion constant *D* is typically calculated using the Einstein relation
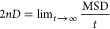
66where *n* is the spatial dimension, MSD is the mean-squared displacement, and *t* is simulation time.^785^ In simulations with finite periodic box, it is standard to “wrap” or “image” positions of molecules such that a molecule crossing a unit cell boundary is translated to the opposite side. This effect must be removed via “unwrapping” for MSD calculation,^786^ which is done in either Cartesian or fractional coordinates.

Constant volume (NVT) or constant energy (NVE) ensembles are recommended for diffusion calculations, as they minimize perturbations to dynamical variables. In MD codes tracking atomic virials like CHARMM, another advantage of using NVT is that the system viscosity and particle diffusion constants can be computed from the same trajectory to compare with experiment.^788^ Unwrapping is straightforward under constant volume, where Cartesian coordinates can be used. However, it can be confounded by volume fluctuations in NPT simulations. For example, the heuristic unwrapping scheme (in which the position of a particle is unwrapped by comparing its current wrapped position to its unwrapped position at the previous time step) used by several MD software packages with Cartesian coordinates was shown to introduce cumulative errors in molecule’s position and calculated MSDs.^789^ A new method called the “toroidal view preserving scheme” was proposed by the same group to correctly unwrap such simulations.^784^

CHARMM avoids the preceding problem by always unwrapping coordinates in fractional space (*i.e.*, the space where each unit cell vector is transformed into 3 orthonormal vectors and positions are mapped onto this lattice) before projecting the coordinates back in Cartesian space. In fractional coordinates, the box fluctuations are removed, and MSD vs *t* plots with the correct slope are produced. Noise at longer simulation times can be mitigated by projecting the coordinates back into Cartesian space using the average unit cell vectors; the keyword for this operation in CHARMM is XFLUC.

**Figure 22:**
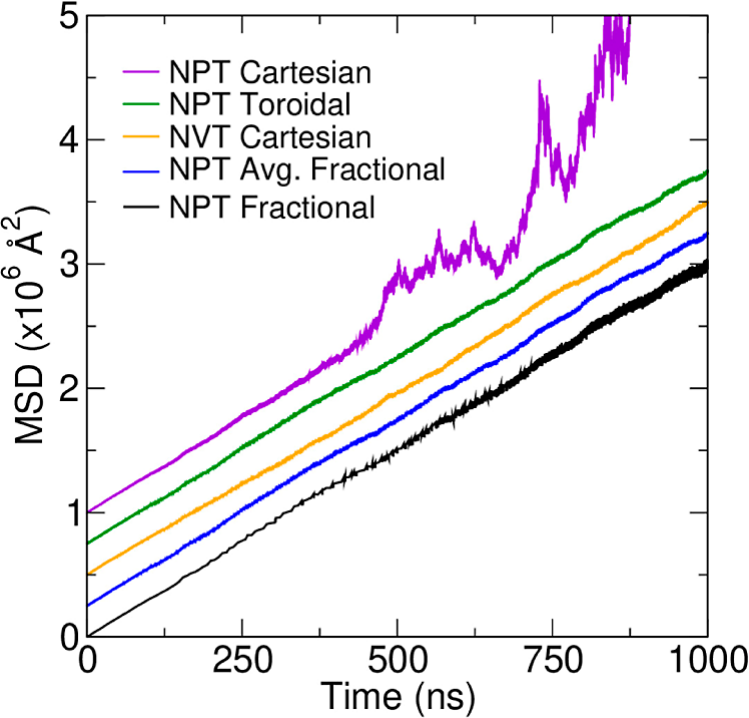
MSD vs *t* for 1-μs NVT and NPT simulations of 1340 TIP3P waters. The plots are offset by intervals of 0.25 × 10^6^ Å^2^ on the *y*-axis to better distinguish them. From top to bottom, violet: NPT with Cartesian unwrapping; green: NPT with toroidal view preserving scheme; orange: NVT with Cartesian unwrapping; blue: NPT with unwrapping in fractional space and using average unit cell vectors (CHARMM XFLUC); black: NPT with unwrapping in fractional space (CHARMM method). Trajectories were run with OpenMM and analyzed with CPPTRAJ version 6.19.3.^787^

The MSD is often computed using multiple time origins as a difference correlation function, where any deviation is indicative of a problem.^784^ For comparison, Figure 22 shows the single time origin MSD vs *t* for 1340 TIP3P waters at 20 °C for NPT and NVT simulations. The NPT simulation was unwrapped in four ways: Cartesian coordinates (non-CHARMM), CHARMM fractional coordinates, CHARMM fractional coordinates with average box dimensions (XFLUC), and toroidal view preserving.^784^ The NPT simulation unwrapped with the Cartesian scheme (violet) shows a significant accumulation of error and deviation from linear behavior after 500 ns. The increase in noise with simulation time for fractional space unwrapping (black) is essentially eliminated by projecting back onto the average unit cell vectors (blue). The resulting MSD vs *t* is practically indistinguishable from the results obtained with NVT (orange) or the toroidal view preserving scheme (green). Diffusion constants obtained from any of the non-Cartesian methods are therefore statistically equal.

**Figure 23:**
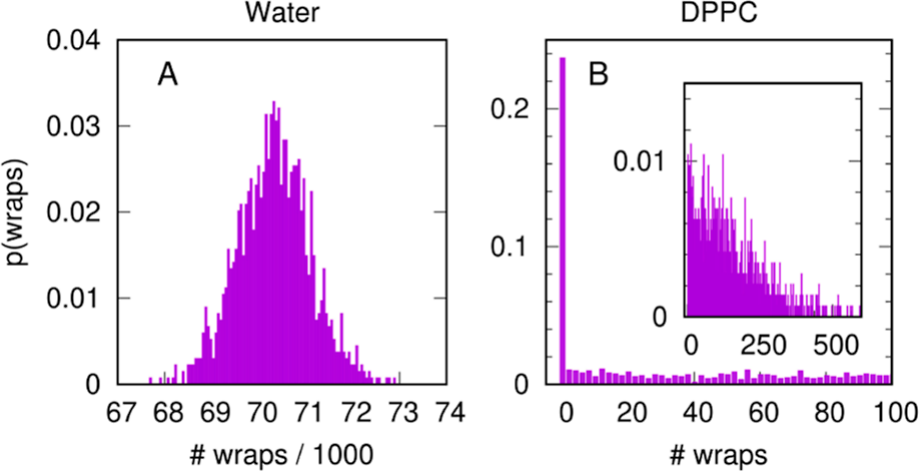
Probabilities of wrap counts for water molecules from a 1-μs NPT MD simulation of TIP3P water and for a bilayer containing 288 DPPC molecules from 5 replicate 400-ns MD simulations.^571^ The inset omits the large peak for DPPC with zero wraps; the mean number of lipid wrap counts is 111.5.

Note that even for Cartesian unwrapping, the first several hundred nanoseconds appear unaffected. Comparison of the wrapping frequency distribution for water and for self-diffusion in a DPPC bilayer shows stark differences (Figure 23). Wrapping events for water are 2 or 3 orders of magnitude more frequent than lipids, and the distribution is approximately Gaussian. The distributions for lipids are Poisson-like with zero being the most frequent value.

Errors obtained from the Cartesian unwrapping scheme are related to the number of wrapping events for a given molecule. Evaluation of lipid diffusion in bilayers from published simulations^571^ shows that for larger, slower moving molecules the unwrapping method had very little effect on the results. Table 3 shows that the standard deviation over five replicate simulations (bottom row) is an order of magnitude larger than that over the three different unwrapping methods (last column).

**Table 3:** *D*_MSD_ (in Units of 10^–7^ cm^2^/s) for NPT DPPC Bilayers with Drude2023 FF, with Cartesian (Cart.), Fractional (Frac.), and Toroidal (Tor.) Unwrapping

Rep #	Cart.	Frac.	Tor.	Avg	Std
1	0.71	0.71	0.70	0.71	0.001
2	0.73	0.73	0.73	0.73	0.001
3	0.74	0.74	0.75	0.74	0.001
4	0.67	0.67	0.67	0.67	0.003
5	0.69	0.69	0.69	0.69	0.003
Avg	0.71	0.71	0.71	0.71	0.001
Std	0.03	0.03	0.03		

Diffusion constants are also affected by the PBC that causes underestimates of the infinite system-size value. PBC errors in diffusion constants in isotropic systems are relatively modest, approximately 10%, and can be corrected by the Yeh–Hummer formula.^790^ The PBC correction for diffusion in lipid bilayers requires the periodic Saffman–Delbrück model.^791,792^ It is quite large for lipid self-diffusion, approximately 3-fold for bilayers with 288 lipids, and should not be overlooked. Our overall recommendations for unwrapping are the following:

- The Cartesian-based method should not be used when the box dimensions can change during a simulation. Though for relatively short simulations of slowly diffusing particles the errors are not substantial (Table 3), accurate methods are readily available and should be used.
- The XFLUC method in CHARMM should only be used when the box dimension fluctuations are less than approximately 15%. This is its major limitation. Though XFLUC would still yield smooth plots, the slope (and hence *D*) could be incorrect if the aspect ratio changes significantly.
- Although the toroidal view preserving method provides a smoother result than with CHARMM fractional coordinates, both methods should give correct answers if the average slope is used.

### 12.3 Calculation of Pressure Profiles in Lipid Bilayers

The lateral pressure profile provides a detailed view of the forces within a planar lipid bilayer with respect to the average bilayer normal (typically *z*-axis). CHARMM provides capability for estimating the spontaneous curvatures in symmetric^557^ and asymmetric bilayers^44^ and the difference in leaflet surface tensions (or differential stress) in asymmetric bilayers.^44,793^ For a simulation system Ω, the volume-averaged virial stress tensor, **σ**, is^794^
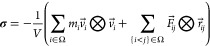
67where *V* is the volume of Ω and *m*_*i*_ and  are the mass and velocity of atom *i*. The symbol ⊗ represents the tensor product between two vectors, , and  is the force exerted from atom *j* on atom *i*. The first and second terms on the right-hand side of Eq. 67 are the kinetic and configurational contributions, respectively. In the configurational virial stress, the contributions from periodic images must be considered for non-bonded interactions. The pressure tensor (**p**) is defined as the negative of the stress tensor, i.e., **p** = −**σ**. For an isotropic system, the bulk pressure is *P* = −Tr(**σ**)/3. CHARMM calculates the virial stress (and thus the pressure) using Eq. 67. While constant pressure can be emulated using Monte Carlo barostat,^34^ the virial is needed to calculate transport properties such as viscosity.^788^

Derivatives of the free energy with respect to virtual transformations^795^ of the periodic box can be computed with the lateral pressure profile *p*_*L*_(*z*) typically along the *z*-direction, normal to the membrane surface
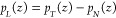
68where *p*_*T*_(*z*) and *p*_*N*_(*z*) are the tangential and normal components of the pressure tensor, respectively. The zeroth moment of the profile is the tension (the derivative of the free energy with respect to area) while the first moment is the derivative of the free energy with respect to the curvature. Interpreted through the Helfrich/Canham Hamiltonian,^796,797^ it provides a convenient route to calculate spontaneous curvature of the leaflet.^798^

For planar lipid bilayers, the slab geometry is a convenient choice for lateral pressure profile calculations
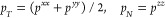
69Here, *p*^*xx*^, *p*^*yy*^, and *p*^*zz*^ are the diagonal elements of **p**(*z*). Since a typical lipid bilayer cannot support in-plane shear strain, there is no off-diagonal coupling of *x* and *y*.

Denoting the *z*-dimension of the simulation box as *L*_*z*_, the bilayer and leaflet surface tensions are given as zeroth-moments of *p*_*L*_(*z*):
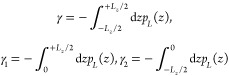
70Without any external force, the bilayer tension must vanish (γ = 0). While leaflets are tensionless (γ_1_ = γ_2_ = 0) in symmetric bilayers, leaflet tensions and their difference (Δ = γ_1_ – γ_2_) in asymmetric bilayers do not necessarily vanish.^799^

The leaflet spontaneous curvature *c*_0_ of a (planar) symmetric bilayer can be calculated from the first moment of *p*_*L*_(*z*)
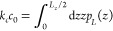
71where *k*_*c*_ is the bending modulus of the leaflet. Spontaneous curvatures of asymmetric bilayers can be calculated with a generalization of Eq. 71.^44,800^

While the bulk pressure tensor can be readily calculated using Eq. 67, the calculation of the local pressure tensor (including the lateral pressure profile) is complicated by the need to assign each contribution to the virial locally in space (here, *z*). Briefly, a vector *contour* is integrated from one force center *i* to the other center *j*, creating a 3D function whose gradient is a Dirac-δ function at each end point multiplied by the force. With this requirement met, the pressure tensor contains spatial correlations of force such that the work required to reshape the box by a virtual deformation can be computed. Many choices of contour satisfy this requirement, leading to the inherent ambiguity of the profile unless complemented by a virtual deformation yielding an observable that resolves the ambiguity.^795^ Because of this complexity, the pressure profile calculation has not been supported in most other simulation programs.

The LOPR module in CHARMM calculates *p*_*T*_ in Eq. 69 where their profiles from the full electrostatics can be obtained by either the Ewald sum^801^ or by the PME method.^176^ Presently there are only two programs in addition to CHARMM that support the pressure profile from full electrostatics: NAMD (by Ewald sum in post analyses) and GROMACS (by PME^802^ in a branch version). Due to the limitation of the Harasima contour^801^ employed for the virial calculation, the normal component *p*_*N*_ (Eq. 69) is not calculated but can be evaluated in post analysis. Furthermore, a planar bilayer cannot support heterogeneous normal pressure. For a bilayer without any external force (γ = 0), *p*_*N*_ is calculated from Eqs. 68 and 70 as
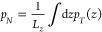
72

The pressure profiles *p*^*xx*^(*z*) and *p*^*yy*^(*z*) are typically calculated by binning the *z*-dimension.^803^ For accurate profiles, the bin size is set typically to ∼1 Å. Alternatively, *p*^*xx*^ and *p*^*yy*^ can be calculated in a binless manner using Fourier series
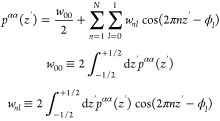
73where α represents *x* or *y* and *z*′ = (*z* – *z*_cm_)/*L*_*z*_ is the fractional *z*-coordinate with respect to the bilayer center *z*_cm_. *N* is the order of the Fourier series, *w*_*nl*_ are Fourier coefficients, and ϕ_0_ = 0 and ϕ_1_ = π/2 are phase shifts for even and odd series. The LOPR module supports both methods for the lateral pressure profile calculation. The Fourier series method is currently supported only in CHARMM, where accurate pressure profiles can be obtained with a moderate number of coefficients, *N* ∼ 20 for typical bilayers with *L*_*z*_ ∼ 80 Å. For simulation systems with larger *L*_*z*_, larger *N* is required.

**Figure 24:**
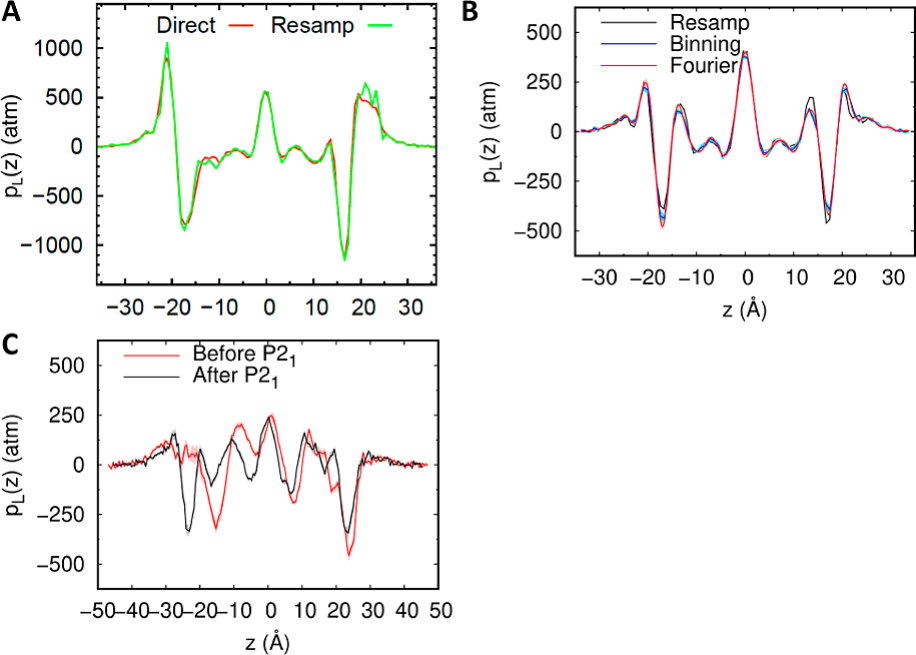
(A) Comparison of pressure profiles from a 100-ns CHARMM simulation of palmitoylsphingomyelin bilayer with complete sampling (Direct) or via the 10% resampling (Resamp) of 100-ps intervals spaced 1-ns apart, using restart files from the fully sampled simulation. The values of the first moment and their standard errors are 0.190 ± 0.009 and 0.192 ± 0.004, respectively. (B) Pressure profiles of a bilayer composed of 72 1,2-dipalmitoyl-*sn*-glycero-3-phosphocholine at 1 atm and 323 K. Data from resampling were obtained by the binning method with 100 bins from the previous CHARMM simulations (black), where 100-ps resampling was done for every 1 ns.^558^ Post analysis using single-step dynamics was tested using 500-ns OpenMM NPT trajectories of the same bilayer, where the pressure profiles were calculated for each frame by both the binning (blue) and Fourier series (red) methods. The numbers of bins and Fourier coefficients were set to 80 and *N* = 20 (Eq. 73), respectively. Standard errors from five 100-ns blocks are shown in cyan and pink areas, respectively, which are smaller than the line thickness except near dips. (C) Pressure profiles for a 1,2-dinervonoyl-*sn*-glycero-3-phosphocholine bilayer with a model peptide of 9 monomeric units of gramicidin A in the upper leaflet at a peptide area fraction ϕ ∼ 0.40 before (red) and after P2_1_ equilibration (black). The P2_1_ PBC in CHARMM allows lipid translocation between bilayer leaflets, which reduces area stress and alters the lateral pressure profile (see Section 12.4). The bilayer midplane was set to *z* = 0. Pressure profiles from the last 300-ns trajectories of 5 replicas from OpenMM were averaged for each asymmetric bilayer, whose standard errors are shown as pink and gray areas (typically smaller than the line thickness). Pressure profiles were calculated by single-step dynamics for each frame of trajectories with 200 bins along the *z*-direction. Panel A reproduced from ref (804). Copyright [2014] Cell Press. Panel C reproduced from ref (44). Copyright [2023] Wiley.

The LOPR module supports both on-the-fly and post analysis calculation of *p*_*T*_(*z*) including the full electrostatics via the PME method. This allows efficient resampling where sparsely sampled coordinate and velocity trajectories from various programs including CHARMM, NAMD, and OpenMM (with a customized Velocity Reporter for trajectory generation)^793^ can be utilized (Figure 24A,B). In the resampling approach, multiple short CHARMM simulations for chosen frames can be run simultaneously, which can be easily realized in typical computational resources. If there are sufficient samples from long simulation times, one can also calculate the lateral pressure profile in a post analysis using a single-step dynamics for each frame with an integration time step shorter than the one used for the original simulations. The post analysis method is faster than the resampling method, and yields sufficiently accurate results from 500-ns trajectories saved at every 10 ps (Figure 24B). For an asymmetric bilayer, the pressure profile is also asymmetric (Figure 24C) from which the non-vanishing leaflet tensions are calculated from Eq. 70.

The LOPR module currently does not support LJ-PME^564^ (Section 8.5) and the polarizable Drude model^571^ which will be supported in future updates. Additionally, it is implemented only for CPU calculations without DOMDEC^25^ (Section 2.2), which results in poor scalability over multiple nodes. Thus, the current LOPR module is suitable practically for only single node-jobs, and parallelization of the code will greatly improve its performance.

### 12.4 P2_1_ Periodic Boundary Condition

A novel approach utilizing the P2_1_ PBC has been implemented to relax differential stress between the leaflets during MD simulations of lipid bilayers. The inherent difficulty in accurately estimating the number of lipids in each layer *a priori* gives rise to the differential stress. The widely used P1 PBC involves tessellating the simulation space with translated images of the box. As an atom exits the simulation box, it is replaced by its image located on the opposite face. Unlike P1, the P2_1_ PBC introduces a half-screw symmetry between the images. In this scheme, the image of the simulation box is not a mere translated copy but a 180°-rotated image translated along the same screw axis. In the CHARMM non-DOMDEC version (DOMDEC is explained in Section 2.2), the screw axis could be oriented in any direction. However, with the Extended Eighth Shell (EES) method^43^ in DOMDEC, the screw axis is constrained to the *x*-axis for enhanced performance. The P2_1_ symmetry operation is denoted as (*x* + 1/2, −*y*, −*z*), representing a half-unit cell length translation along the *x*-axis and reflection along the *y*- and *z*-axes. Reflection along two perpendicular axes is equivalent to 180° rotation along the screw axis where lipids departing from the top layer along the *x*-axis (*yz*-faces) reenter the cell in the bottom layer, and *vice versa*. However, since only the *x*-axis is allowed as the screw axis, lipids leaving the cell along the *xz*-faces reenter along the same leaflet. This can be visualized as a torus-shaped structure along the screw axis where the top layer transitions to the bottom layer in the neighboring image cell. A bilayer simulation starting with 108 lipids on the top layer and 92 lipids in the bottom equilibrates to 100 lipids in both layers using this method.^43^

The scaling performance of the EES method for P2_1_ simulations is similar to that for DOMDEC in P1 simulations. Compared to the latter, there is slightly larger import volume during the message transfer among nodes during the direct space calculations. However, by restricting the screw axis to the *x*-axis, there is a minimal impact of the larger message size on the overall performance. The reciprocal space calculations are done by distributing the charge on the full unit cell. However, forces are calculated only in the asymmetric unit, and extra bookkeeping is also performed to rotate the forces and velocities as the images are 180°-rotated along the *yz* faces.

While it may first appear that the exchange of lipids between the layers would allow only symmetric bilayer simulation, the P2_1_ PBC is specifically useful for setting up asymmetric bilayers by restraining specific lipids to their original leaflet and redistributing others between the leaflets through P2_1_ PBC. The differential stress or the difference in surface tension between the two layers is a consequence of the intrinsic bending and the asymmetric lipid packing. Methods for simulating asymmetric bilayers can be categorized as lipid-based, leaflet-based, and bilayer-based.^793^ In the lipid-based approach termed APL, surface areas of the top and bottom are matched using the area per lipid from homogeneous lipid bilayers. This is the simplest and the most commonly used approach that disregards any coupling between the bilayers and assumes ideal mixing of lipids. The leaflet-based approach termed SA minimizes the differential area strain between the leaflets. It first equilibrates symmetric bilayers corresponding to each leaflet composition and then combines one leaflet from each bilayer. While this removes the differential strain, it also disregards coupling between the two leaflets. The bilayer-based approach termed 0-DS removes the differential stress by adjusting the number of lipids. When these approaches are followed by P2_1_, agreement in mechanical properties significantly improves among APL/P2_1_, SA/P2_1_, and 0-DS/P2_1_.^793^ These findings align with a theoretical framework emphasizing the intricate interplay between bending and asymmetric lipid packing. Torque balance conditions and stress indices provide theoretical support, showcasing promising results for P2_1_ simulations in capturing lipid asymmetry observed in biological membranes.

As another example of the importance of P2_1_ for setting up bilayer simulations, the curvature induced by a peptide in the so-called “peptide-asymmetric bilayer” was studied.^44^ In these simulations, while both layers contained the same types of lipids, the asymmetry was induced by the presence of the peptide in the cis-leaflet. A series of gramicidin A (gA)-based peptides were simulated: a single monomer, a fused tetramer, a fused nonamer, nine gA monomers, and a fused tetramer of a gA mutant whose Trp residues were replaced by Gln. These assemblies were used to investigate effects of the size of chemically similar peptides spanning a single leaflet. Utilizing the APL method mentioned above, systems were created at three distinct peptide area fractions. Subsequently, equilibration employing the P2_1_ PBC was performed. The P1 simulation showed significant condensation of lipids in the trans leaflet, resulting in large differential stress and bilayer bending moment, which relaxed via the exchange of lipids between leaflets in P2_1_ simulation (Figure 24C).

### 12.5 Primary Hydration Shell (PHS) Model

While the representation of solvent surrounding macromolecules should closely approximate physical reality in MD simulations, the common use of a sizable volume of solvent with PBC is computationally expensive. A PHS consisting of 2–3 layers of explicit water molecules around a protein may be sufficient to maintain the conformational stability and dynamics of macromolecules. The initial work^631^ on the PHS model has been refined in two stages. First, the method was tested with hen egg lysozyme, where good agreement with the protein and solvent behavior was observed, including Lipari–Szabo order parameters for N–H main-chain and N–H_2_ side-chain motions on the ps–ns time scale in simulations of 25–150 ns, compared to full PBC treatments.^805^ The original PHS method has a modest half-harmonic restraint of waters to their nearest protein atom, should they become more distant than a threshold value (5.8 Å by default). As a part of this work a simpler GEO restraint has been implemented which saves computer time as it is calculated relative to three perpendicular principal axes that follow the protein frame. The GEO approach follows global conformational changes and is similarly good for the lysozyme tested.

The PHS method was subsequently refined to overcome issues when applied to larger systems.^806^ A neighbor list was implemented to efficiently track the nearest protein atoms to the water oxygen atom, to avoid calculating distances at each step. Also, an asymmetric harmonic potential instead of a half-harmonic one was used to ensure correct water density close to the boundary. In addition, pressure control was implemented, and the confining potential was scaled to keep waters near hydrophobic residues. This approach showed a 14-fold reduction in computing time for a 82-kDa protein. Future developments of the PHS model should handle situations with extensive structural changes^807^ and association between proteins where treatment of long-range force is important.^808^

### 12.6 Hydration Map

Surface hydration of proteins and nucleic acids are important for their biological function,^809^ which has long been a subject of computer simulation.^810^ For measuring location-dependent average behavior of water molecules near biomolecular surfaces, the COORdinates SMAP (Solvation MAP) command has been implemented in CHARMM.^811,812^ It divides the simulation water box into a grid of cubic cells (default size: 0.7 Å^3^, half the radius of a water molecule), and locally calculates time-averaged properties of water within each cell. To account for protein motion during simulation, coordinate frames are aligned to a reference structure so that the calculated map is relative to the surface. In the current implementation, local water density (SDENsity), translational diffusion coefficient (DIFTrans), and the average number of hydrogen bonds formed by water molecules (HBONd) can be calculated. By default, water oxygen atoms are used for calculation. Other atoms such as ions can be selected as ‘solvent’ atoms, to build the corresponding maps. This capability has been used to find the preferred location of sodium ions around a double-stranded DNA as the center of the minor groove, between the ‘double water spines.’^812^

The solvation map can be saved as a data file for further analysis, or an MRC format electron density map for visualization, e.g., by using the UCSF ChimeraX.^813^ Since coordinate frames are oriented to a reference structure, calculated water densities around flexible loops may become low. To examine hydration around a moving loop, a separate solvation map can be built by selecting only the loop as the orientational reference.

The water density map can be used to calculate the solvation free energy.^814^ For *N* cells surrounding the protein under consideration, if the water density of cell *i* during the simulation is ρ_*i*_ and denoting the bulk water density as ρ_*b*_, the free energy of water for *N* cells is
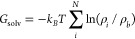
74Since the size of a cell is smaller than that of a water molecule (0.7 Å by default), a high water density at cell *i* means that the cell is visited more frequently rather than water molecules are packed more tightly. The underlying idea for Eq. 74 is that a more frequently visited cell has a lower free energy (a favorable location) compared to the bulk water. To use Eq. 74 in practice, cells corresponding to the first hydration shell are selected, for which a distance cutoff of 4.5 Å from heavy atoms of the protein, and water density cutoff of 0.034 Å^–3^ (*cf., ρ*_*b*_ = 0.0333 Å^–3^) are used.^814^ Compared to the popular grid inhomogeneous solvation theory (GIST),^815,816^ the above method does not require the protein to be constrained during simulation, where constraint on proteins drastically alters surface hydration. And the measured solvation energy values are in physically more reasonable range compared to those from GIST.^814^ Calculation of the density-based solvation free energy is being implemented in CHARMM as the COORdinates SMAP SLVE command.

### 12.7 Conformational Entropy

Conformational entropy is an essential component of the conformational free energy of a biomolecule. A widely used class of entropy calculation methods rely on quasi-harmonic approximation where frequencies of different vibrational modes are used to estimate conformational entropy.^817,818^ However, they cannot account for transitions between states. With increases in computational power, more direct evaluation of entropy from distributions of degrees of freedom (DOF), in particular, backbone and side-side chain dihedral angles has become possible. If all *N* DOFs are treated independently, the cost of entropy calculation scales linearly with *N*. However, since DOFs can be mutually correlated (e.g., by correlated motion of side chains forming contacts), higher order corrections should be made, which amounts to calculating multivariate histograms. The maximal information spanning tree (MIST) approach systematically handles higher order corrections in such a way that the estimated entropy monotonically approaches its asymptotic value.^819,820^ It should be noted that, an accurate calculation of higher order terms requires a greater number of coordinate frames since for *n*-th order correlation, the total number of bins for the histogram scales with *N*^*n*^. With a limited number of coordinate frames, bins will be sparsely populated, leading to an increased statistical uncertainty. In practice, MIST calculation up to the second order is good for most purposes since it provides statistically reliable result, fast to calculate, and correlations beyond the second order do not usually contribute significantly.

By limiting DOFs to an amino acid side chain, MIST can be used to estimate the side-chain entropy of individual residues. In this case, since *N* is small (e.g., total number of dihedral angles of a side chain), calculation up to the third order MIST can be done for higher accuracy. This method has been used to calculate changes in the side-chain entropy for binding of proline-rich ligands to an SH3 domain.^821^ In this study, “entropy hotspots” were identified where the side-chain entropy of remote residues in the SH3 domain increases upon ligand binding. This arises from the rearrangement of contacts across the protein’s surface that makes the side chains of entropy hotspots to become more mobile upon ligand binding. While initially developed as a separate code,^821^ the COORdinates MIST command is currently being implemented in CHARMM.

### 12.8 Identifying Non-Polar Contacts

In CHARMM, hydrogen bonds can be readily identified by the COORdinates HBONd command, which works either for a single structure or across coordinate frames. In comparison, identifying non-polar contacts has been less established. It is often desirable to determine non-polar contacts at the level of individual residues rather than between pairs of atoms. Measuring distances between C_α_ atoms or centers of mass between side chains of non-polar residues and applying an *ad hoc* cutoff distance does not provide an accurate picture of non-polar contacts. CHARMM now has the COORdinates DISTance RESIdue command. Without the RESIdue keyword, all pairwise distances between two groups of selected atoms are reported. With the RESIdue keyword, pairwise minimum distances between residues in the two groups (e.g., between two domains) are reported. To identify non-polar contacts, selecting atoms with the absolute value of charges less than 0.3*e* (*e* = 1.6 × 10^–19^ C) and distance cutoff of 3 Å can be used. These are based on charges of non-polar hydrogen atoms and their van der Waals radii (1.32 Å). In this way, physical contacts between non-polar residues can be identified and further processed to analyze their dynamics, i.e., occupancy, formation, and breakage.^822,823^

### 12.9 Vectorial Analysis of Long-Range Concerted Motions in MD Trajectories

Long-range concerted motions in proteins and other biomolecules is best captured with a correlation coefficient (CC) based on covariance using Euclidean distances between entries of a position-vector time series. This coefficient, DCOR, is a vector equivalent of Pearson’s CC or a generalized CC.^824^ The relative accuracy of DCOR is established by an assessment conducted using vector displacements generated with a known CC.^825^ DCOR is least sensitive to angular variation between two vectors compared to Pearson’s CC or a vector CC. Nor is DCOR as sensitive to large variations between vector components compared to the (scalar) generalized CC, which was found to give inflated CCs relative to the actual values when only one of the vector components is highly correlated.

The DCOR value between any two vector time series can be computed using the CORREL module. The vector dimensions need not be equal. For each time series, a matrix of intravector Euclidean distances between all pairs of time points in the series is used to calculate the covariance. DCOR reflects both linear and non-linear correlations,^825^ and can detect long-distance concerted motions that neither Pearson’s CCs nor the generalized CCs can reveal.^826^

### 12.10 Other Updated Preparation and Analysis Features

#### 12.10.1 System Generation

The residue sequence to be used in the generation of a segment in the PSF can be read from the ATOM and/or HETATM records in a PDB file with a specified chain or segment ID to the READ SEQU PDB command. If there are residues in the PBD file that do not exist in the RTF (Residue Topology File), or with names that differ from those used in the RTF, it is possible to either skip those residues or map the name in the PDB to the name in the RTF. The sequence can also be read from the SEQRES records, which is useful when there are missing residues in the PDB-file. These enhancements allow the generation of a PSF directly from a PDB-file without editing it.

#### 12.10.2 Trajectory Handling

CHARMM can read trajectories that are contained in multiple files, and normally applies a number of checks to ensure that the set of files constitutes a valid contiguous trajectory with no overlaps or gaps. Sometimes it is desirable to override these checks, for instance, to analyze a set of independent replicate trajectories together to obtain overall statistics. These checks can be disabled, making it possible to mix files that are not contiguous or differ in a other ways (e.g., time step or coordinate saving frequency), as long as they use the same PSF.

Binary trajectories can be read automatically, irrespective of big-endian or little-endian format of the trajectory (and of the executable). The CHARMM file OPEN command also allows endianness to be specified with a keyword.

#### 12.10.3 Time Series Analysis in the CORREL Module

Time series data can be mapped to specified interval, which is useful, e.g., to avoid spurious jumps in dihedral angles (MANTIM command). Time series of protein secondary structure content can also be extracted using CORREL.

#### 12.10.4 Similarity Analysis of Snapshots from Trajectory Files

The RMSDYN command now allows different numbers of frames in two trajectories to be compared. Two new metrics have been added: the interatomic average coordinate difference without superposition (DIFF option), and the RMS distance (DRMS option), which compares interatomic distances in one structure with the corresponding distances in another structure, obviating the need for structural superposition. The latter is also available for single coordinate sets, and for time-series analysis in the CORREL module.

## 13 Concluding Discussion

Since its first publication in 1983,^4^ CHARMM has been continuously developing as the need and demand for computational biophysics and biochemistry grew. The present review of the major developments since 2009^3^ highlight improvements as well as new capability, many of which are uniquely available in CHARMM. This review may thereby serve as a guide for exploring new methods in addition to providing a broad overview of the current state of the art.

The advances also reflect changes in the research landscape at large. Faster simulation engines are needed as the molecular systems to study are becoming larger and also as the computer hardware continues to develop. With its modularity and flexibility, CHARMM now employs a number of engines either within the program or through APIs for external engines, which include DOMDEC, BLaDE, CHARMM/OpenMM, and the newly developed apoCHARMM (Section 2). Thus, the multicore/multithread scaling and speed of CHARMM should be comparable to those of other fast simulation engines presently available, while maintaining highest accuracy.

Accessibility is another practical issue, for which CHARMM is now readily available for academic and non-profit laboratories.^827^ While the powerful CHARMM scripting language enables sophisticated tasks, a potential downside is the steep learning curve and difficulty in programming. This is being addressed through the development of pyCHARMM (Section 3.1). Its Python-based workflow also allows leveraging the capability of the Python language. Additionally, pyCHARMM is beginning to serve as a teaching platform from which the general principles and ideas of molecular modeling and biomolecular simulation can be taught. Preparation of the simulation system and the CHARMM script can also be done through CHARMM-GUI (Section 3.3).

Among other significant features of CHARMM are a wide range of docking and sampling methods (Section 4–Section 7) and various energy functions including implicit solvent and membranes, coarse graining, as well as a host of constraint capabilities (Section 8–Section 9). The QM/MM methods described in Section 11 are uniquely available in CHARMM. Likewise, CHARMM has distinct capabilities in system preparation, structure manipulation, and coordinate/trajectory analysis (Section 12). Finally, the ever-expanding CHARMM FF (Section 10) is becoming the *de facto* standard that is widely adopted in other simulation packages.

The extensive capabilities of CHARMM enable simulations and quantitative analyses of systems ranging from small molecules to large biomolecular assemblies and membrane systems at both atomistic and CG levels. Beyond studying small model systems, CHARMM is now increasingly used to tackle problems of practical importance that involve larger sizes, longer simulation times, and more extensive sampling, which will continue to grow with advances in computer hardware and methodologies. For the latter, CHARMM has been the testbed for new computational methods, thereby it stays on the forefront of biomolecular modeling and simulation with a fertile link to its developers and users. We anticipate CHARMM will continue to play an essential role for addressing current problems and also for opening new avenues of research in biomolecular systems.
